# Author Correction: Progerinin, an optimized progerin-lamin A binding inhibitor, ameliorates premature senescence phenotypes of Hutchinson-Gilford progeria syndrome

**DOI:** 10.1038/s42003-021-01843-6

**Published:** 2021-03-02

**Authors:** So-mi Kang, Min-Ho Yoon, Jinsook Ahn, Ji-Eun Kim, So Young Kim, Seock Yong Kang, Jeongmin Joo, Soyoung Park, Jung-Hyun Cho, Tae-Gyun Woo, Ah-Young Oh, Kyu Jin Chung, So Yon An, Tae Sung Hwang, Soo Yong Lee, Jeong-Su Kim, Nam-Chul Ha, Gyu-Yong Song, Bum-Joon Park

**Affiliations:** 1 Department of Molecular Biology, College of Natural Science, Pusan National University, Busan, Korea; 2 Department of Food Science, College of Agricultural Science, Seoul National University, Seoul, Korea; 3 Department of Pharmacy, College of Pharmacy, Chungnam National University, Daejeon, Korea; 4 New Drug Development Center, Daegu-Gyeongbuk Medical Innovation Foundation, Daegu, Korea; 5 Institute of Animal Medicine, College of Veterinary Medicine, Gyeongsang National University, Jinju, Korea; 6 Cardiovascular Center, Research Institute for Convergence of Biomedical Science and Technology, Pusan National University Yangsan Hospital, Yangsan, Korea

**Keywords:** Senescence, Drug discovery

Correction to: *Communications Biology* 10.1038/s42003-020-01540-w, published online 04 January 2021.

The source for lonafarnib was left out in the original version of the Article. The amended “Animal experiments” section may be found below, with the updated text shown in bold.

**Animal experiments**. Animal experiments were performed in a facility certified by the Association for Assessment and Accreditation of Laboratory Animal Care in compliance with animal policies approved by Pusan National University. The mouse work was performed under the study protocol PNU-2019-2181, as approved by the Institutional Animal Care and Use Committee. *Lmna*^*G609G/609G*^ mice were generated by timed mating of heterozygous *Lmna*^*G609G/+*^ provided by Carlos López-Otín (Universidad de Oviedo, Asturias, Oviedo, Spain). SLC-D011 was mixed with dimethyl sulfoxide (DMSO) and phosphate-buffered saline (PBS). It was then intraperitoneally injected into mice (20 mg/kg twice per week from 5-week-old). SLC-D011 and lonafarnib were orally administrated to mice daily at a concentration of 10 mg/ml in monoolein-based solution. **Lonafarnib was generously provided by Merck, the Progeria Research Foundation (PRF) and the PRF Lonafarnib Pre-clinical Drug Supply Program**. Control mice were treated with monoolein-based solution alone in the same way. *Lmna*^*G609G/609G*^ mice were treated with clear chemical solution throughout the life span, starting from 5 weeks of age. *Lmna*^*G609G/+*^ mice were treated via intraperitoneal and oral administrations, starting from 32 weeks of age.

