# Author Correction: Progerinin, an optimized progerin-lamin A binding inhibitor, ameliorates premature senescence phenotypes of Hutchinson-Gilford progeria syndrome

**DOI:** 10.1038/s42003-026-10449-9

**Published:** 2026-06-27

**Authors:** So-mi Kang, Min-Ho Yoon, Jinsook Ahn, Ji-Eun Kim, So Young Kim, Seock Yong Kang, Jeongmin Joo, Soyoung Park, Jung-Hyun Cho, Tae-Gyun Woo, Ah-Young Oh, Kyu Jin Chung, So Yon An, Tae Sung Hwang, Soo Yong Lee, Jeong-Su Kim, Nam-Chul Ha, Gyu-Yong Song, Bum-Joon Park

**Affiliations:** 1 Department of Molecular Biology, College of Natural Science, Pusan National University, Busan, Korea; 2 Department of Food Science, College of Agricultural Science, Seoul National University, Seoul, Korea; 3 Department of Pharmacy, College of Pharmacy, Chungnam National University, Daejeon, Korea; 4 New Drug Development Center, Daegu-Gyeongbuk Medical Innovation Foundation, Daegu, Korea; 5 Institute of Animal Medicine, College of Veterinary Medicine, Gyeongsang National University, Jinju, Korea; 6 Cardiovascular Center, Research Institute for Convergence of Biomedical Science and Technology, Pusan National University Yangsan Hospital, Yangsan, Korea

Correction to: *Communications Biology* 10.1038/s42003-020-01540-w, published online 04 January 2021

In the version of the article initially published, due to a figure assembly mistake, the Kaplan-Meier survival cure shown as Fig. 4c was an inadvertent duplicate of Fig. 3c. The figure is now updated in the HTML and PDF versions of the article.

