# Supplementary material for: Progerinin, an optimized progerin-lamin A binding inhibitor, ameliorates premature senescence phenotypes of Hutchinson-Gilford progeria syndrome
Source: Commun Biol. 2021 Jan 4;4:5. doi: 10.1038/s42003-020-01540-w (PMC7782499; doi:10.1038/s42003-020-01540-w)

Figure S1

A

CSIM→ASIM in Lamin A

```
seq1  399 ------------------------------------------------------------
LA1   453 GGGGTGGCAGCTTCGGGGACAATCTGGTCACCCGCTCCTACCTCCTGGGCAACTCCAGCC

seq1  399 --------CAGAGCCCCCAGAACAGCAGCATCATGTAATCTGGGACCTGCCAGGCAGGGG
LA1   513 CCCGAACCCAGAGCCCCCAGAACTGCAGCATCATGTAATCTGGGACCTGCCAGGCAGGGG

seq1  451 TGGGGGTGGAGGCTTCCTGCGTCCTCCTCACCTCATGCCCACCCCCTGCCCTGCACGTCA
LA1   573 TGGGGGTGGAGGCTTCCTGCGTCCTCCTCACCTCATGCCCACCCCCTGCCCTGCACGTCA

seq1  511 TGGGAGGGGGCTTGAAGCCAAAGAAAAATAACCCTTTGGTTTTTTTCTTCTGTATTTTTT
LA1   633 TGGGAGGGGGCTTGAAGCCAAAGAAAAATAACCCTTTGGTTTTTTTCTTCTGTATTTTTT
```

B

CSIM→ASIM in Progerin

```
Seq1  361 AGCCTGCCGACAAGGCATCTGCCAGCGGCTCAGGAGCCCAGAGCCCCCAGAACAGCAGCA
PG2   333 AGCCTGCCGACAAGGCATCTGCCAGCGGCTCAGGAGCCCAGAGCCCCCAGAACTGCAGCA

Seq1  421 TCATGTAATCTGGGACCTGCCAGGCAGGGGTGGGGGTGGAGGCTTCCTGCGTCCTCCTCA
PG2   393 TCATGTAATCTGGGACCTGCCAGGCAGGGGTGGGGGTGGAGGCTTCCTGCGTCCTCCTCA

Seq1  481 CCTCATGCCCACCCCCTGCCCTGCACGTCATGGGAGGGGGCTTGAAGCCAAAGAAAAATA
PG2   453 CCTCATGCCCACCCCCTGCCCTGCACGTCATGGGAGGGGGCTTGAAGCCAAAGAAAAATA

Seq1  541 ACCCTTTGGTTTTTTTCTTCTGTATTTTTTTTTCTAAGAGAAGTTATTTTCTACAGTGGT
PG2   513 ACCCTTTGGTTTTTTTCTTCTGTATTTTTTTTTCTAAGAGAAGTTATTTTCTACAGTGGT

Seq1  601 TTTATACTGAAGGAAAAACACAAGCAAAAAAAAAAAAAAAAAAAAAAAAAAAAAAAAAAA
PG2   573 TTTATACTGAAGGAAAAACACAAGCAAAAAAAAAAAAAAAAAAAAAAAAAAAAAAAAAAA
```

C

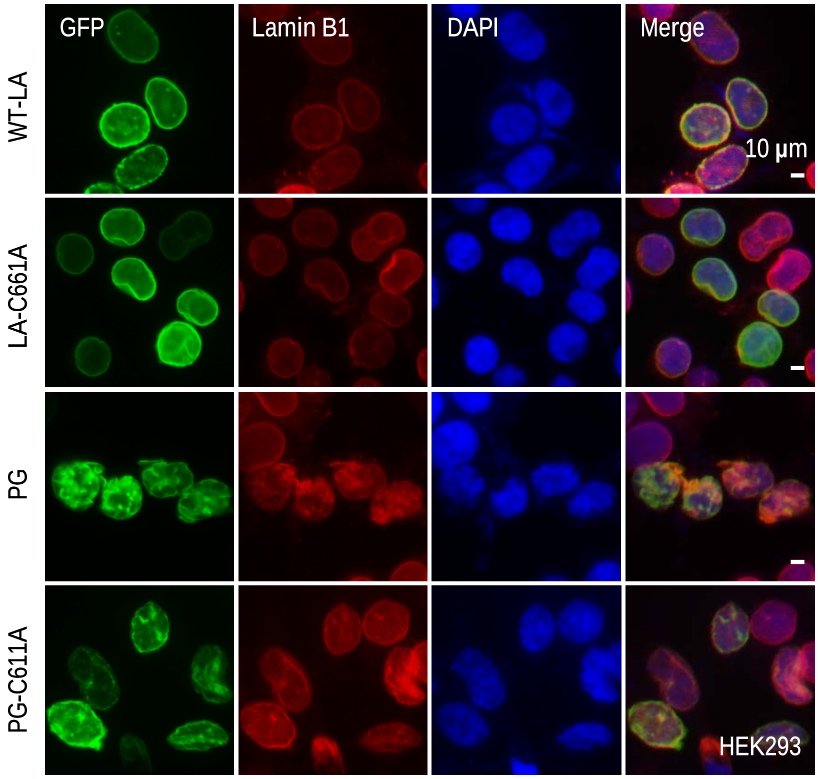

D

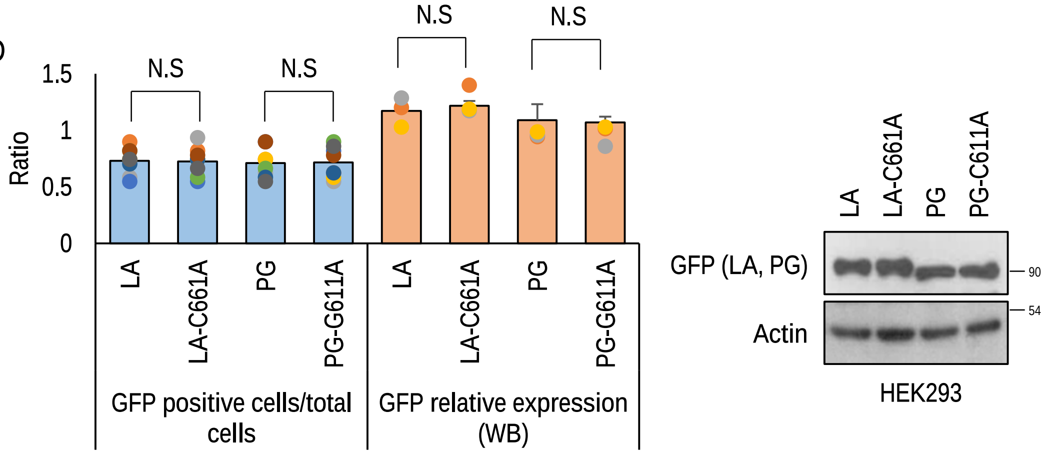

Figure S1 (continue)

E

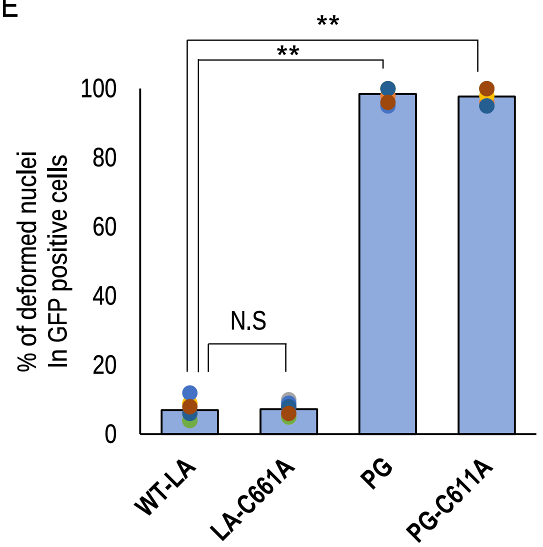

F

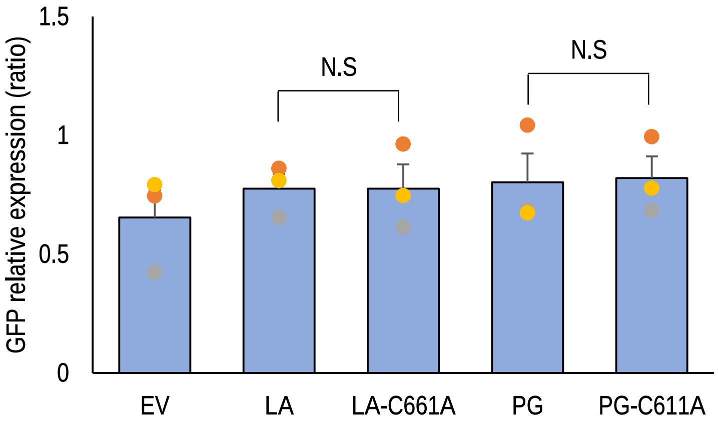

G

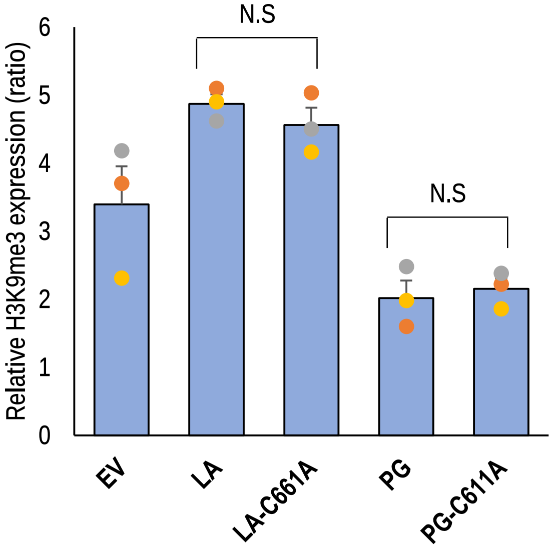

H

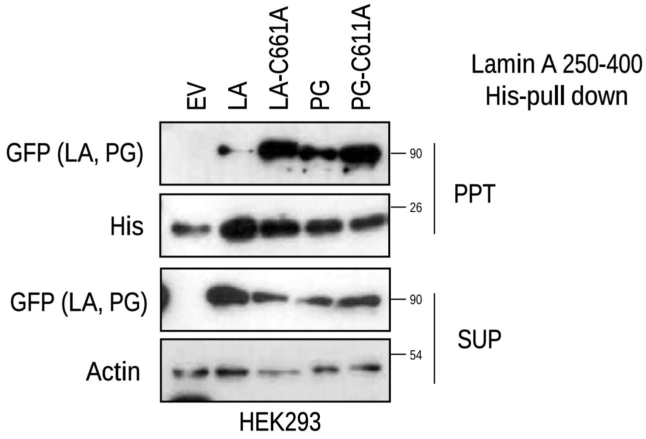

Fig. S1. Generation of point mutation in CaaX motif of lamin A and progerin for blocking farnesylation. **A**. Sequence of expression vector encoding LA-C661A was created by single point mutation in CaaX motif. WT-LA with CSIM sequence was mutated to ASIM. **B**. Sequence of expression vector encoding progerin-C611A with a single point mutation in CaaX motif. Progerin with CSIM sequence was mutated to ASIM. **C**. Transient transfection of HEK293 cells with LA-C661A or PG-C611A seems to be indistinguishable from that of authentic WT-LA, or progerin. **D**. Measurement of proteins (GFP-LA, GFP-LA-C661A, GFP-PG, and GFP-PG-C611A) expression by counting of GFP-positive cells and western blot analysis. The blue bar graph shows the ratio of GFP expressing cells in the total number of cells. For cell counting, eight fluorescence images were randomly selected and the number of GFP expressing cells were calculated. The orange bar graph shows the relative expression of GFP-tagged proteins in western blot (n = 3 independent experiment; two-tailed Student's t-test), N.S: not significant. Quantification of western blot by Image J software (National Institute of Health, NIH). **E**. Quantification of nuclear deformation in GFP positive HEK293 cells. At least 100 cells/sample/experiment were counted by two independent researchers. The statistical significance was analyzed by Student's t-test. **$p < 0.001$, *$p < 0.05$, N.S: not significant. **F**. Quantification of GFP-tagged expression vector encoding empty (EV), wild-type lamin A (WT-LA), lamin A-C661A (LA-C661A), progerin, or progerin-C611A. Blots of GFP expression were quantified using Image J software. (n = 3 independent experiment; two-tailed Student's t-test), N.S: not significant. **G**. Quantification of H3K9me3 expression. Blots of H3K9me3 expression were quantified using Image J software. **H**. His pull-down assay using lysates from HEK293 cells, transiently transfected with WT-LA, LA-C661A, progerin, or progerin-C611A, after incubation with the middle region sequence of lamin A (250-400 aa; His-lamin A 250-400) for 30 min at RT.

Figure S2

A

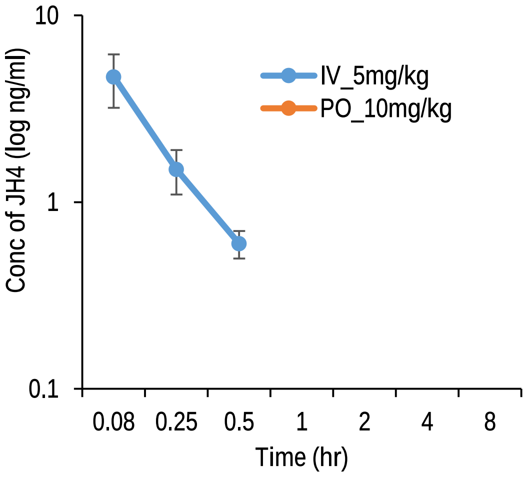

B

Plasma concentration after intravenous injection at a dose of 5mg/kg (n=3)

Time(hr)	Subject1	Subject2	Subject3	Mean(ng/ml)	SD
0.08	3.3	4.6	6.3	4.7	1.5
0.25	1.7	1.1	1.8	1.5	0.4
0.5	0.5	0.7	0.5	0.6	0.1
1	BQL	BQL	BQL	BQL	BQL
2	BQL	BQL	BQL	BQL	BQL
4	BQL	BQL	BQL	BQL	BQL
8	BQL	BQL	BQL	BQL	BQL
24	BQL	BQL	BQL	BQL	BQL

Plasma concentration after oral administration at a dose of 10mg/kg (n=3)

Time(hr)	Subject1	Subject2	Subject3	Mean(ng/ml)	SD
0.08	BQL	BQL	BQL	BQL	BQL
0.25	BQL	BQL	BQL	BQL	BQL
0.5	BQL	BQL	BQL	BQL	BQL
1	BQL	BQL	BQL	BQL	BQL
2	BQL	BQL	BQL	BQL	BQL
4	BQL	BQL	BQL	BQL	BQL
8	BQL	BQL	BQL	BQL	BQL
24	BQL	BQL	BQL	BQL	BQL

BQL : Below Quantifiable Limit (< 1ng/ml)

C

PK Parameter after intravenous injection at a dose of 5mg/kg (n=3)

Subject	T1/2 (hr)	AUCall (hr*ng/ml)	AUCINF_obs (hr*ng/ml)	%AUC Extrap	Cl_obs (ml/min/kg)	MRTINF_obs (hr)	Vss_obs (L/kg)
1	0.2	1	1.1	10.8	73261.3	0.2	939.4
2	0.2	1.2	1.4	11.7	58977.3	0.2	675.9
3	0.1	1.7	1.8	5.4	46951.3	0.1	406.4
Mean	0.1	1.3	1.4	9.3	59730	0.2	673.9
SD	0	0.3	0.3	3.4	13171.1	0	266.5

Fig. S2. In vivo analysis of JH4. **A.** In vivo PK analysis of JH4. Although JH4 could obviously suppress premature aging phenotypes in *Lmna*^{G609G} progeria model mice via *i.p* injection, it rapidly disappeared after PO treatment (undetectable level). **B**. Time-dependent JH4 chemical concentration in blood after intravenous injection at a dose of 5 mg/kg or oral administration at a dose of 10 mg/kg. **C.** PK parameters after intravenous injection at a dose of 5 mg/kg.

Figure S3

A

Chemical No.	Binding affinity	Expression
Con	+++	+++
SLC-D011	+	+
JH010	+	+
EJDe01	+++	+++
EJDE02	+++	+++
EJDe03	+++	+++
EJDe04	+++	Non
EJDe05	+++	Non
EJDe06	+++	Non
EJDe07	+++	Non
EJDe08	+++	Non
EJDe09	++++	Non
EJDe10	++++	Non
EJDe11	++++	Non
EJDe12	+++	Non
SKY-006-066	+	+++
SKY-006-067	+	+++
SYK-006-069	++	+++
SYK-006-070	++	+++
SYK-006-072	+	+++

Chemical No.	Binding affinity	Expression
SYK-007-008	++	+++
SYK-007-011	++	+++
SYK-007-022	+++	+++
SYK-007-023	+++	+++
SYK-007-025	+++	+++
SYK-007-026	+++	+++
SYK-007-031	+++	+++
SYK-007-019	++	+++
SYK-007-029	++	+++
SYK-007-033	++	++++
SYK-007-034	++	++++
SYK-007-036	++	+++
SYK-007-037	++	+++
SYK-007-053	++	+++
SYK-007-054	+++	+++
SYK-007-059	++	Cell death
SYK-007-060	++	Cell death
SYK-007-066	++	++++
SYK-007-067	++	+++

Chemical No.	Binding affinity	Expression
KSY-010-011	+++	+++
KSY-010-006	++	+++
KSY-010-007	++	+++
KSY-010-008	++	+++
KSY-010-009	++	+++
KSY-010-025	+++	+++
KSY-010-026	+++	+++
KSY-010-027	+++	+++
KSY-010-028	+++	+++
KSY-010-030	+++	+++
KSY-010-036	+++	+++
KSY-010-037	++	+++
KSY-010-038	+++	+++
KSY-010-039	+++	+++
KSY-010-040	++	+++
KSY-010-061	+++	+++
KSY-010-063	+++	+++
KSY-010-069	+++	+++

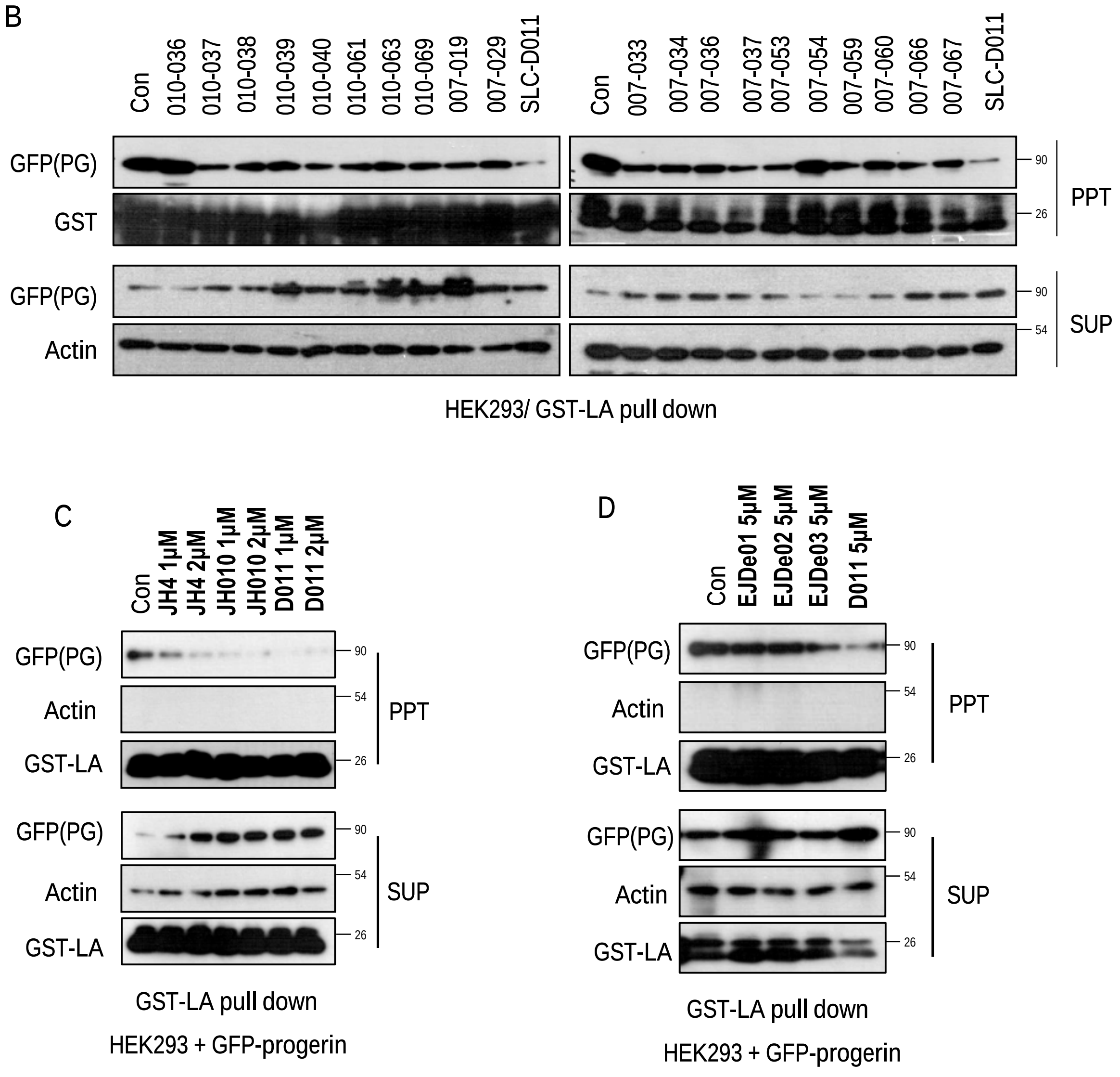

Figure S3 (continue)

E

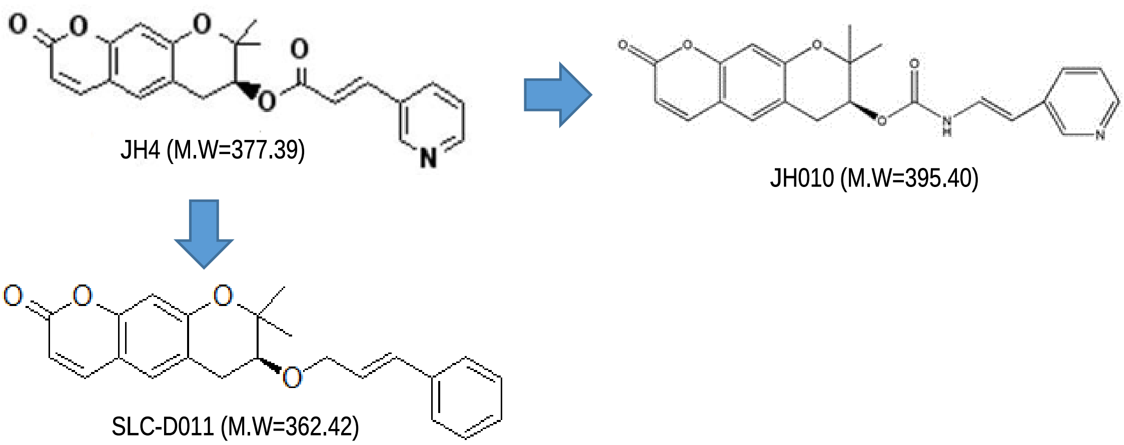

F

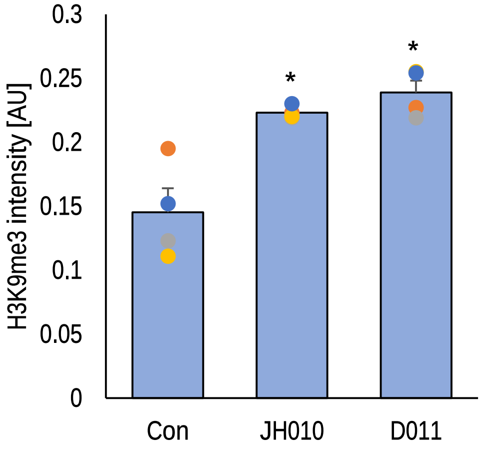

G

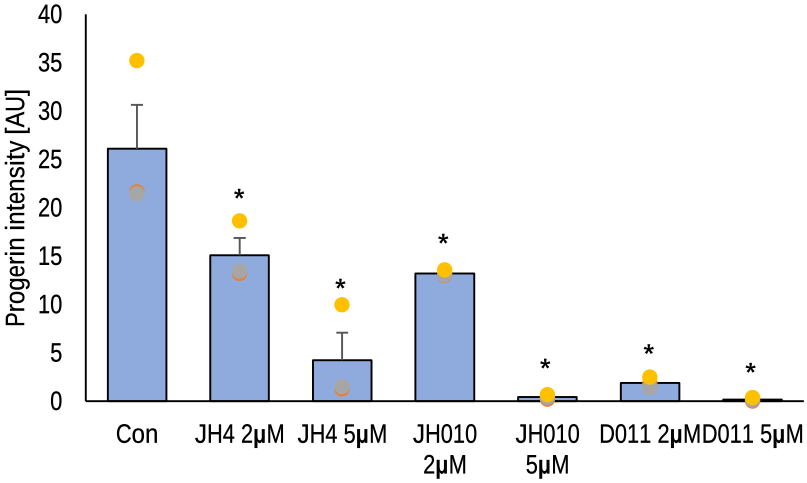

H

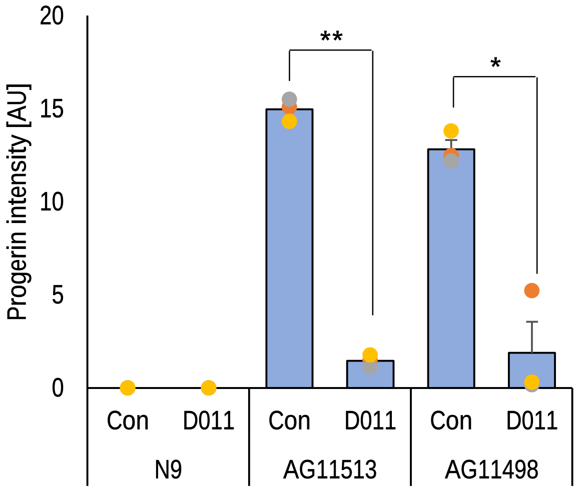

I

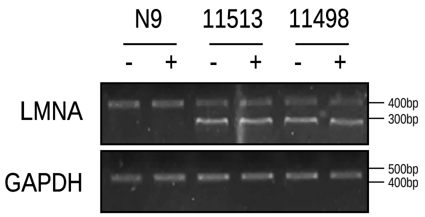

J

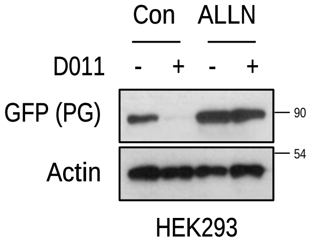

K

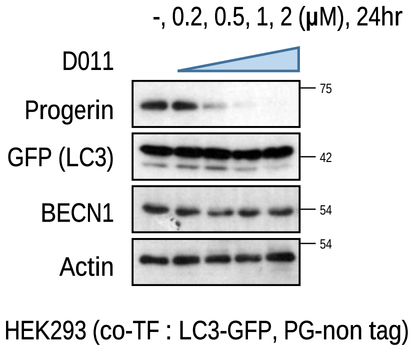

L

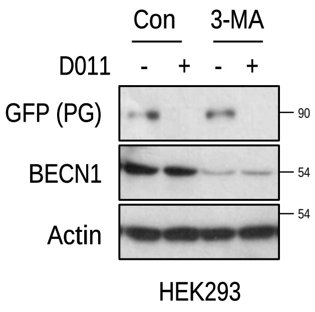

Figure S3 (continue)

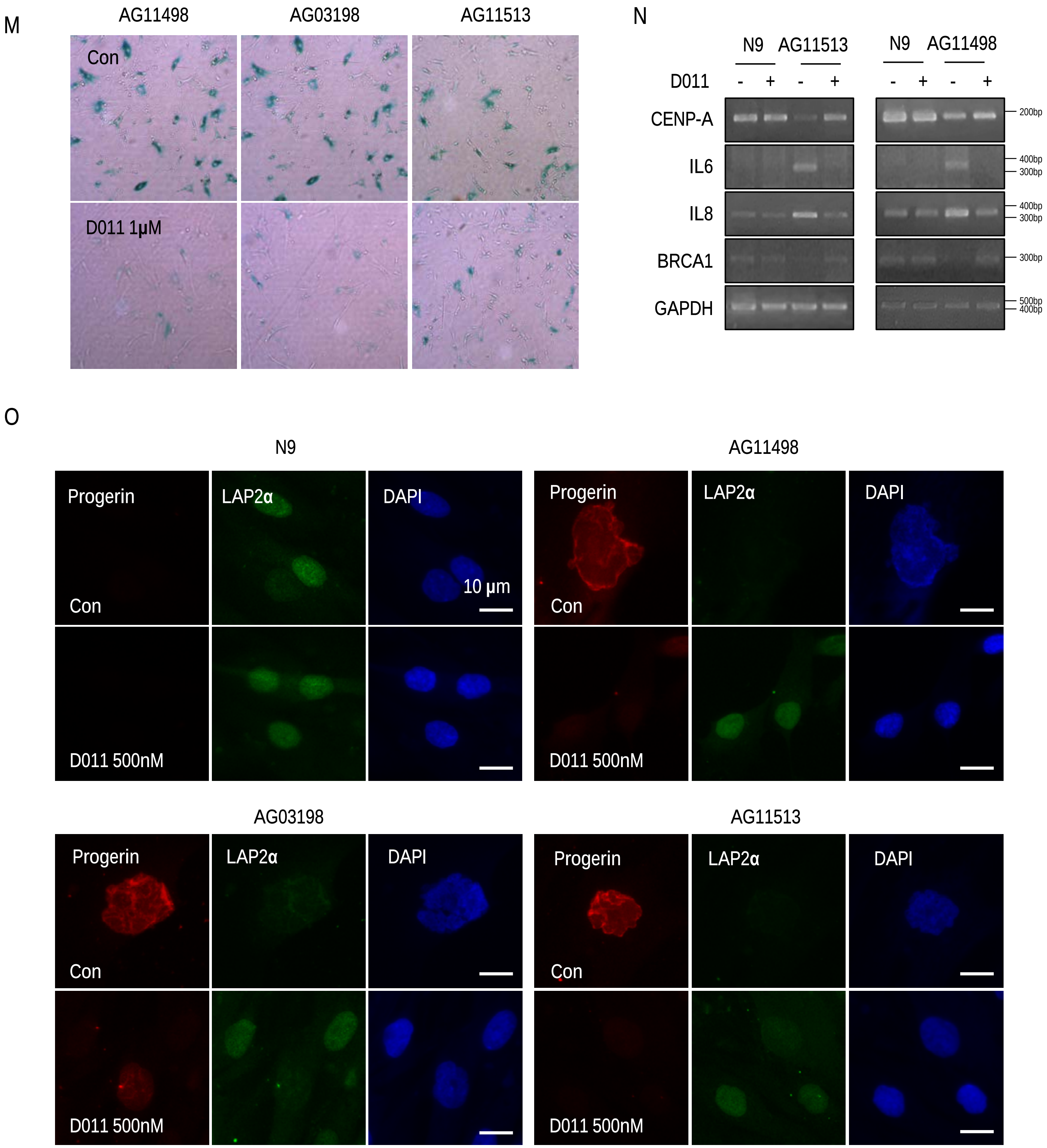

Fig. S3. Chemical screening with JH4 derivatives (a total of 53 chemicals except JH010 and SLC-D011 were used). **A.** Summarized tables for effects of JH4 derivatives. '+' means strength of binding affinity or expression level after treatment of chemicals. Middle columns of tables show binding affinity between progerin and lamin A. Third columns of tables show level of progerin expression. Some chemicals showed severe cytotoxicity (cell death or none). **B, C, and D**. Examples of screening based on pull-down assay. All chemicals were tested using GST pull-down assay. JH010 and SLC-D011 (D011) showed similar or better inhibition activity than JH4. **E**. Generation of JH4 derivatives. To prevent rapid digestion *in vivo*, side chain was replaced by amide bond (JH010) or ether bond (SLC-D011). **F.** The bar graph shows the intensity of H3K9me3 levels after treatment with JH010 and D011 in HGPS cells (n = 3 independent experiments; two-tailed Student's t-test), $*p < 0.05$. **G.** The bar graph shows the intensity of progerin levels after treatment with JH4, JH010, and SLC-D011 in HGPS cells (n = 3 independent experiments; two-tailed Student's t-test), $*p < 0.05$. **H.** The bar graph shows the intensity of progerin levels after treatment with SLC-D011 in fibroblasts from normal person and patients with HGPS (n = 3 independent experiments; two-tailed Student's t-test), $**p < 0.001$, $*p < 0.05$. **I.** Transcriptional levels of progerin are not affected by treatment with SLC-D011. Total RNA was extracted after treatment with SLC-D011 for 7 days. The PCR products correspond to wild-type prelamin A and progerin transcripts. The upper fragment near 400bp observed in both healthy control and the patient-derived samples indicates prelamin A. The lower fragment with an approximate length of 290bp was identified in the HGPS cell line but not in the control sample. **J.** ALLN, a proteasome inhibitor, block the downregulation of progerin by SLC-D011. **K.** HEK293 cells were transiently transfected with GFP-conjugated expression vector encoding LC3 and treated with SLC-D011 in a dose-dependent manner for 24h. **L.** SLC-D011 and 3-MA were co-treated for 24h after transient transfection of HEK293 cells with GFP-conjugated expression vector encoding progerin. **M.** SLC-D011 suppressed expression of SA-β-gal in HGPS cells. **N.** Reduced expressions of CENP1 and BRCA1 in HGPS cells were restored by SLC-D011 (1 μM). Conversely, increased expressions of IL6 and IL8 were suppressed by treatment with SLC-D011 in HGPS cells. **O.** Treatment with SLC-D011 (1 μM) for 7 days

induced the expression of Lap2α in HGPS cells. AU, arbitrary units.

Figure S4

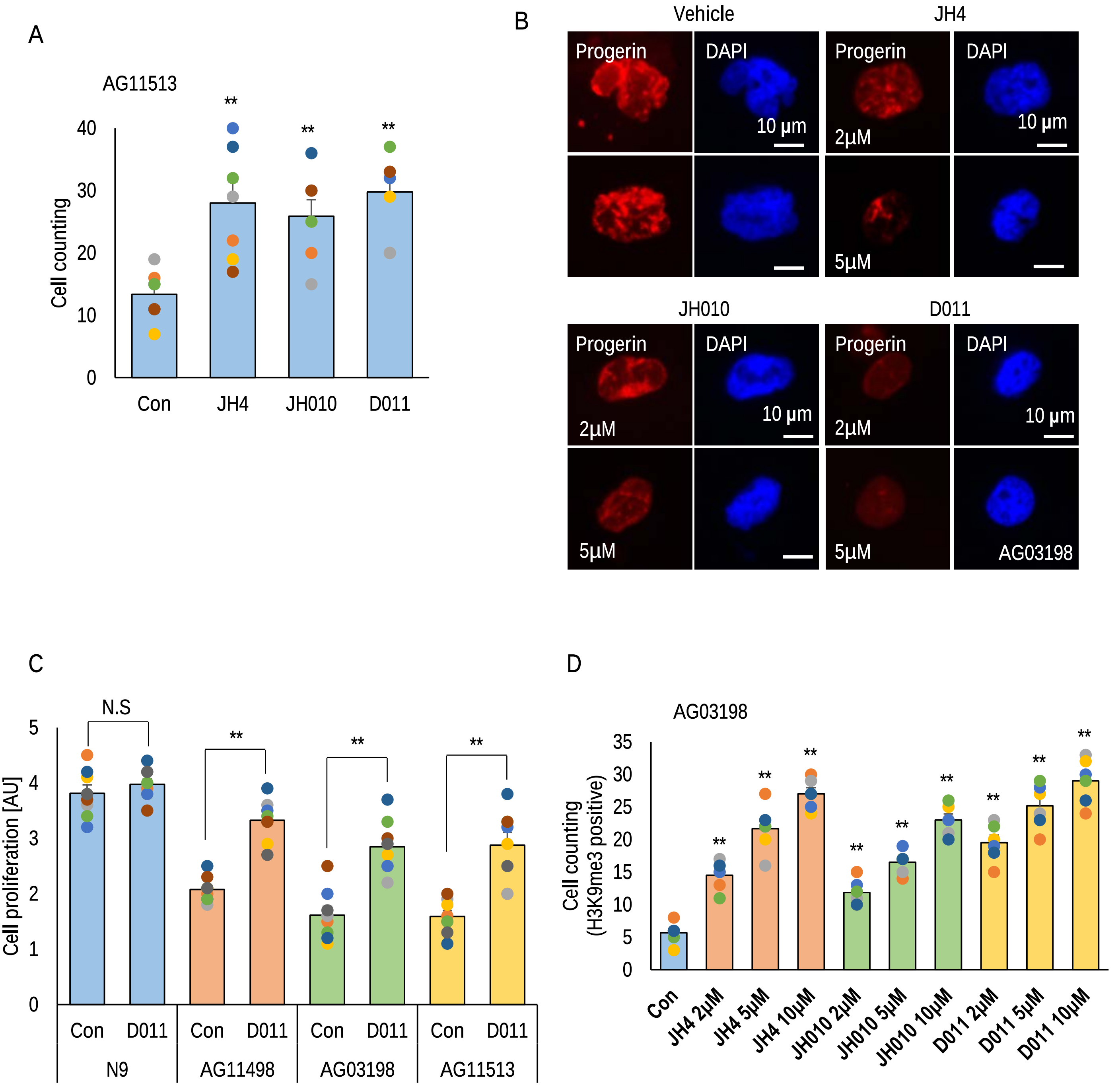

A
AG11513
Cell counting
40
30
20
10
0
**
**
**
Con
JH4
JH010
D011
B
Vehicle
JH4
JH010
D011
Progerin
DAPI
10 μm
2μM
5μM
AG03198
C
N.S
**
Cell proliferation [AU]
5
4
3
2
1
0
Con
D011
N9
AG11498
AG03198
AG11513
D
AG03198
Cell counting
(H3K9me3 positive)
35
30
25
20
15
10
5
0
Con
JH4 2μM
JH4 5μM
JH4 10μM
JH010 2μM
JH010 5μM
JH010 10μM
D011 2μM
D011 5μM
D011 10μM

Figure S4 (continue)

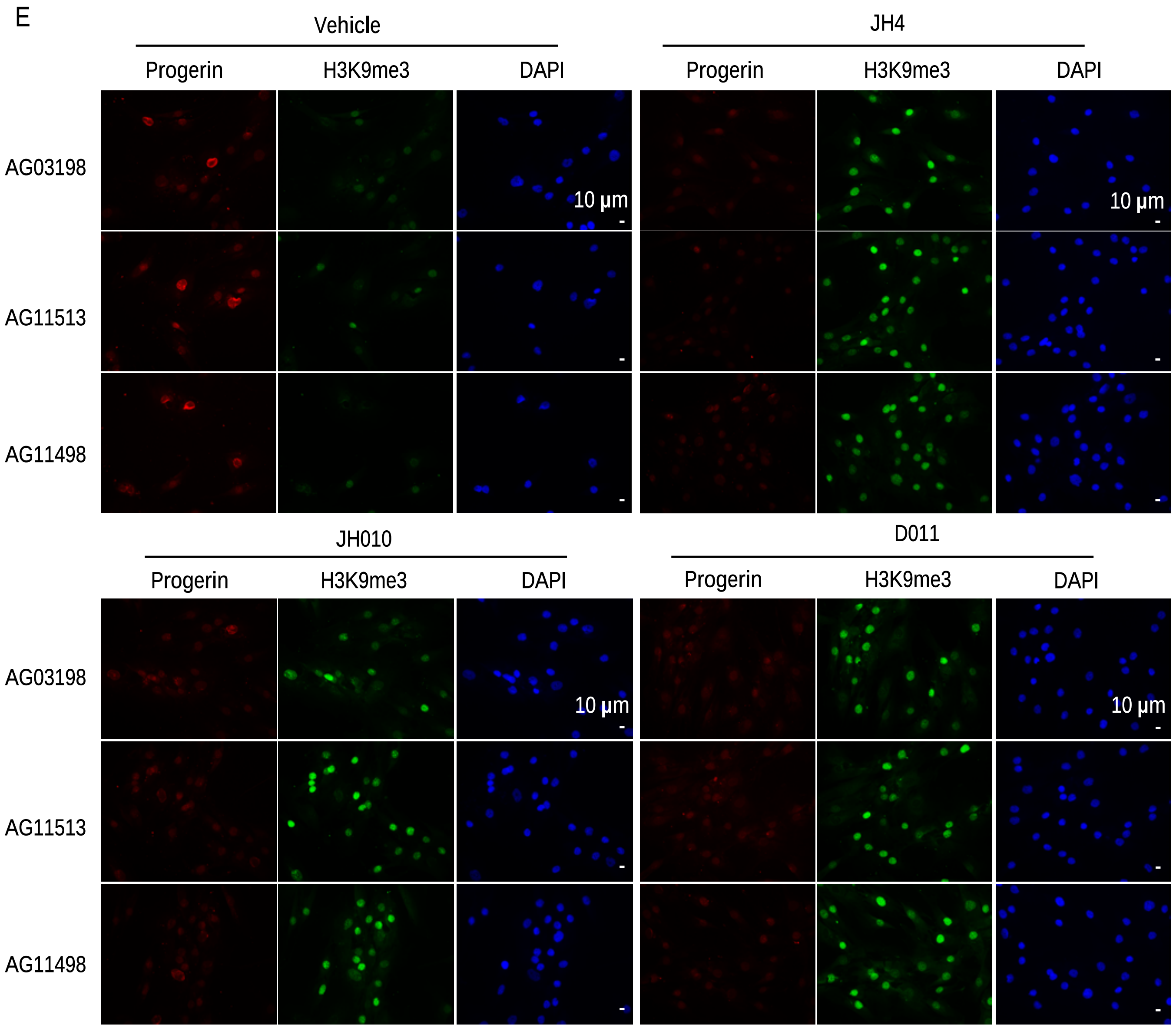

Fig. S4. Effects of JH4, JH010, and SLC-D011 in ameliorating nuclear deformation of HGPS fibroblasts. **A.** JH4, JH010, and SLC-D011 increase population of HGPS cells (n = 3 independent experiment; two-tailed Student's t-test), $^{**}p<0.001$. **B.** SLC-D011 ameliorates nuclear morphology and reduces progerin expression. To confirm the effect of each chemical, HGPS cells (AG03198) were incubated with each chemical for 7 days and stained with anti-progerin (red). Nuclear aberrations were ameliorated by chemicals, particularly SLC-D011. DAPI indicated DNA. **C.** Treatment with SLC-D011 (1 μM) for 7 days increased cell proliferation in three different HGPS cells but not in normal fibroblasts (n = 3 independent experiments; two-tailed Student's t-test), $^{**}p < 0.001$, N.S: not significant. AU, arbitrary units. **D.** JH4, JH010, and SLC-D011 increase H3K9me3-positive cells dose dependently (n = 3 independent experiment; two-tailed Student's t-test), $^{**}p<0.001$. **E.** JH4, JH010, and SLC-D011 show similar activities in ameliorating nuclear deformation. After incubation with chemicals for five days, cells were stained with progerin and H3K9me3 antibodies. All values are reported as mean ± SD.

Figure S5

A

PK Parameter after oral administration at a dose of 10mg/kg (n=5)

Subject	$T_{1/2}$ (hr)	Tmax (hr)	Cmax (ng/ml)	AUCall (hr*ng/ml)	AUCINF_obs (hr*ng/ml)	%AUC Extrap	MRTINF_obs (hr)
1	1.1	0.5	4872.3	7072.6	7166.3	1.3	2.1
2	1	0.5	8891.7	12910.4	13049.6	1.1	2.5
3	1.2	0.5	9039.5	11312.7	11484.4	1.5	2.1
4	2	0.5	6805.8	12751.8	13535.7	5.8	2.5
5	3.4	0.5	6835.8	12306.2	14627.6	15.9	4.2
Mean	1.7	0.5	7289	11270.7	11972.7	5.1	2.7
SD	1	0	1725.7	2428	2915.1	6.3	0.9
Bioavailability (BA): 69.9							

B

Plasma protein binding

Compound	Human (% Bound)	Rat (% Bound)
JH010	98.4	97.6
Dexamethasone (Reference)	65.5	84.4
Warfarin (Reference)	99.1	99

C

Liver microsomal stability (% remaining during 30min)

Compound	Human (%)	Rat (%)	Mouse (%)
JH010	41.8	16.1	25
Verapamil (Reference)	9		

D

Plasma stability (% remaining)

Compound	Human		Rat	
	30min	120min	30min	120min
JH010	> 100	> 100	> 100	97.4

E

CYP inhibition

	CYP1A2	CYP2C9	CYP2C19	CYP2D6	CYP3A4
JH010	89.4	92.6	100	24.1	26.5
Ketoconazole (Reference)	99.1	100	99.5	> 100	27.3

Fig. S5. In vitro ADME results of JH010. **A.** Pharmacokinetic parameters of JH010. **B.** Analysis of plasma protein binding assay. **C.** Liver microsomal stability test. **D.** Plasma stability. **E.** CYP inhibition. Based on in vitro ADME test results, JH010 was ruled out from final drug candidates.

Figure S6

A

PK Parameter after oral administration at a dose of 5mg/kg (n=3)

Subject	$T_{1/2}$ (hr)	T_{max} (hr)	C_{max} (ng/ml)	AUCall (hr*ng/ml)	AUC_{inf}_obs (hr*ng/ml)	%AUC Extrap	MRT_{inf}_obs (hr)
1	missing	2	33.1	137.4	missing	missing	missing
2	missing	2	28.6	147.2	missing	missing	missing
3	missing	4	27.5	147.7	missing	missing	missing
Mean		2.7	29.7	144.1			
SD		1.2	3	6.8			
Bioavailability (BA): 65.9%							

B

Plasma protein binding

Compound	Human (% Bound)	Rat (% Bound)
SLC-D011	99.9	99.8
Dexamethasone (Reference)	62.1	78.8
Warfarin (Reference)	98.9	98.2

C

Liver microsomal stability (% remaining during 30min)

Compound	Human (%)	Rat (%)	Mouse (%)
SLC-D011	21.5	17.4	59.7
Verapamil (Reference)	16.2		

D

Plasma stability (% remaining)

Compound	Human		Rat	
	30min	120min	30min	120min
SLC-D011	> 100	> 100	83.6	75.1

E

CYP inhibition

	CYP1A2	CYP2C9	CYP2C19	CYP2D6	CYP3A4
SLC-D011	81.6	78.4	28.8	91.7	92.5
Ketoconazole (Reference)	97.3	95.4	> 100	98.4	29.9

Fig. S6. In vitro ADME results of SLC-D011. **A.** PK parameters of SLC-D011. **B.** Plasma protein binding. **C.** Liver microsomal stability. **D.** Plasma stability. **E.** CYP inhibition.

Figure S7

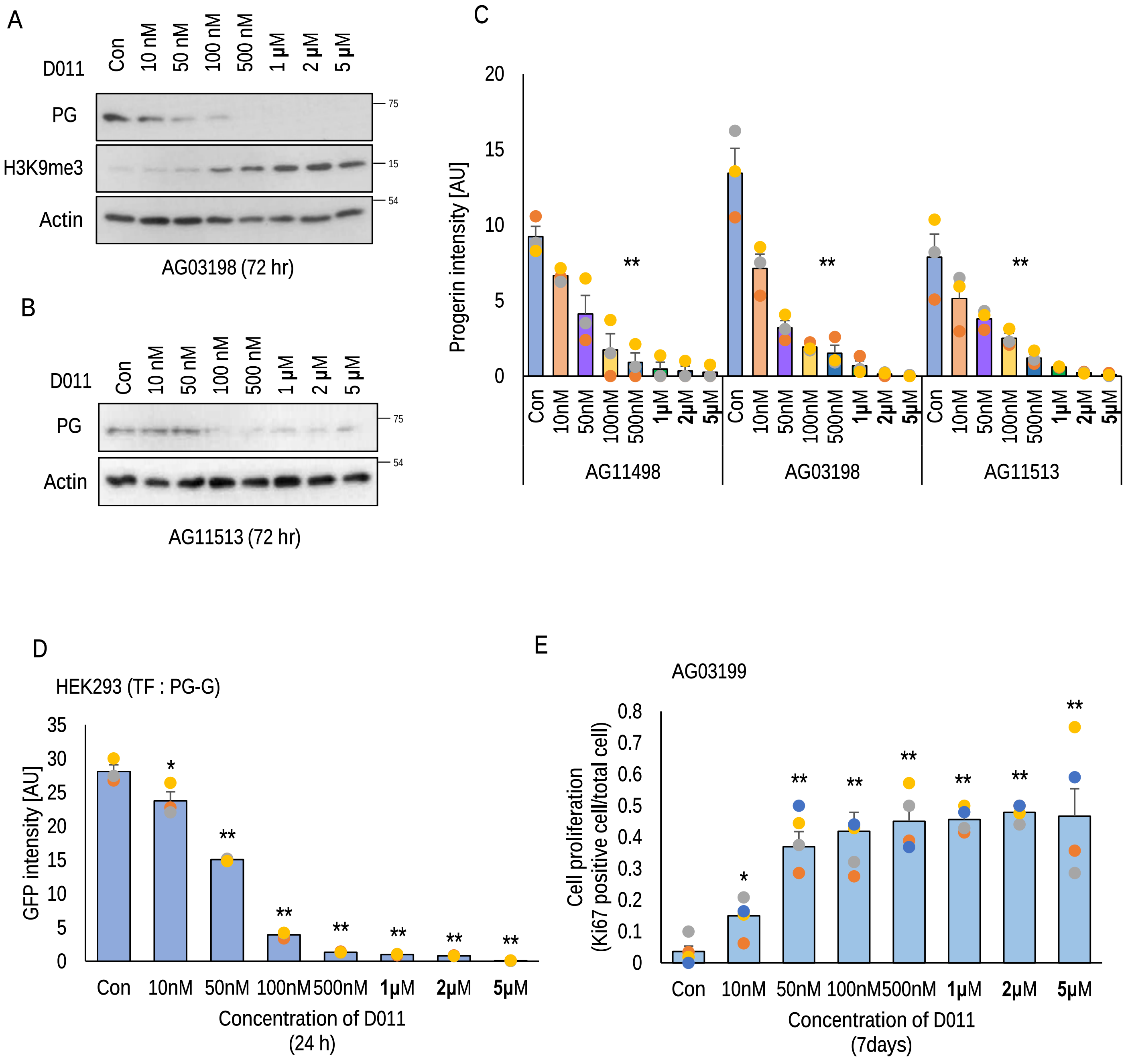

A
D011
Con
10 nM
50 nM
100 nM
500 nM
1 μM
2 μM
5 μM
PG
H3K9me3
Actin
75
15
54
AG03198 (72 hr)
B
D011
PG
Actin
AG11513 (72 hr)
C
Progerin intensity [AU]
20
15
10
5
0
**
Con
10nM
50nM
100nM
500nM
1μM
2μM
5μM
AG11498
AG03198
AG11513
D
HEK293 (TF : PG-G)
GFP intensity [AU]
35
30
25
*
Concentration of D011
(24 h)
E
AG03199
Cell proliferation
(Ki67 positive cell/total cell)
0.8
0.7
0.6
0.5
0.4
0.3
0.2
0.1
Concentration of D011
(7days)

Figure S7 (continue)

F

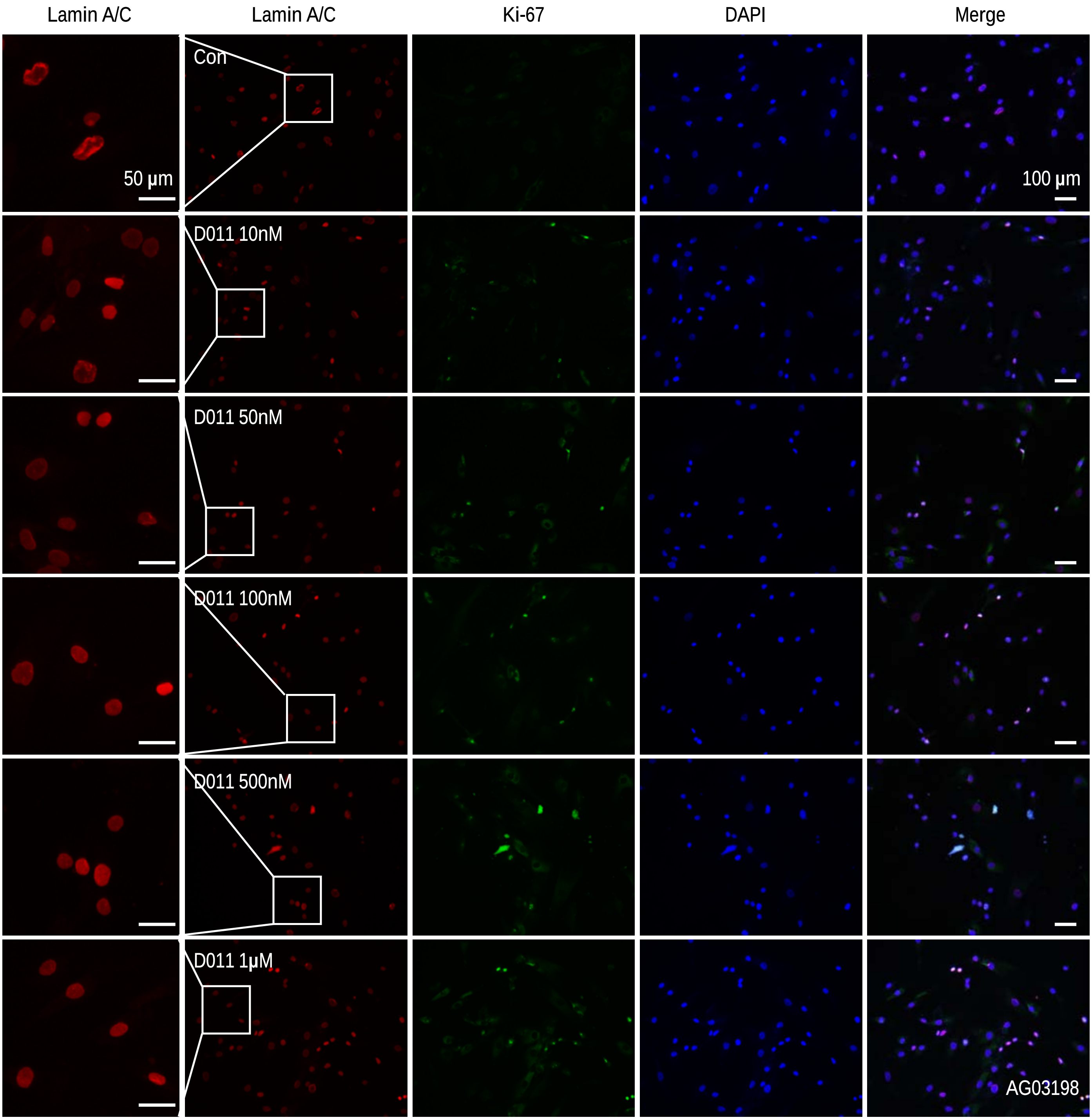

Figure S7 (continue)

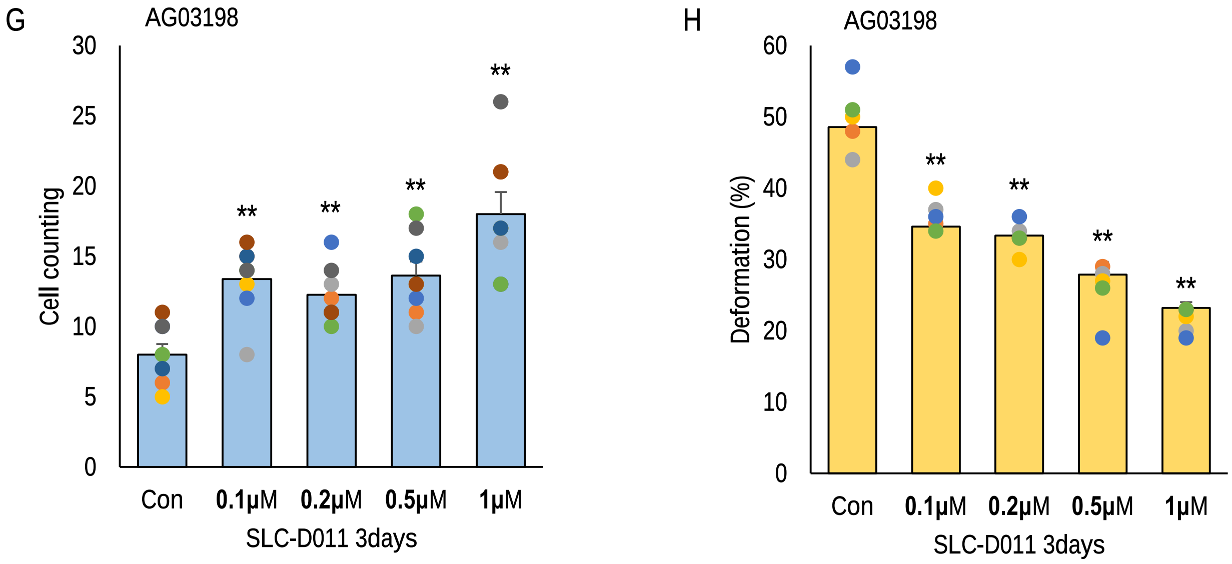

Fig. S7. Therapeutic effect of SLC-D011 at a low concentration. **A and B.** SLC-D011 suppresses progerin expression and induces H3K9me3 expression in HGPS patient-derived fibroblasts (AG03198; **A** and AG11513; **B**) dose-dependently. HGPS cells were treated with SLC-D011 at a low concentration (from 10 nM to 5μM) for 3 days. **C.** The bar graph shows the intensity of progerin levels in HGPS cells after treatment with SLC-D011 in a dose-dependent manner (n = 3 independent experiment; two-tailed Student's *t*-test), $^{**}p<0.001$. **D.** The bar graph shows the intensity of GFP-conjugated progerin levels in HEK293 cells after treatment with SLC-D011 dose-dependently. HEK293 cells were transiently transfected with GFP-conjugated expression vector encoding progerin and treated with SLC-D011 for 24 h, (n = 3 independent experiments; two-tailed Student's *t*-test), $^{**}p < 0.001$, $^{*}p < 0.05$. **E.** SLC-D011 increases Ki67-positive cells dose-dependently (n = 3 independent experiments; two-tailed Student's *t*-test), $^{**}p < 0.001$, $^{*}p < 0.05$. **F.** SLC-D011 increases cell proliferation but reduces progerin expression in HGPS cells. To confirm these effects, HGPS cells (AG03198) were incubated with SLC-D011 for 7 days and stained with anti-progerin (red) and anti-Ki67 (green) antibodies. DAPI indicated DNA. **G.** SLC-D011 increases population of HGPS cells (AG03198) dose dependently (n = 3 independent experiments; two-tailed Student's *t*-test), $^{**}p < 0.001$. **H**. Nuclear aberrations were ameliorated by SLC-D011 dose-dependently (n = 3 independent experiment; two-tailed Student's *t*-test), $^{**}p < 0.001$. AU, arbitrary units.

Figure S8

Injection scheme

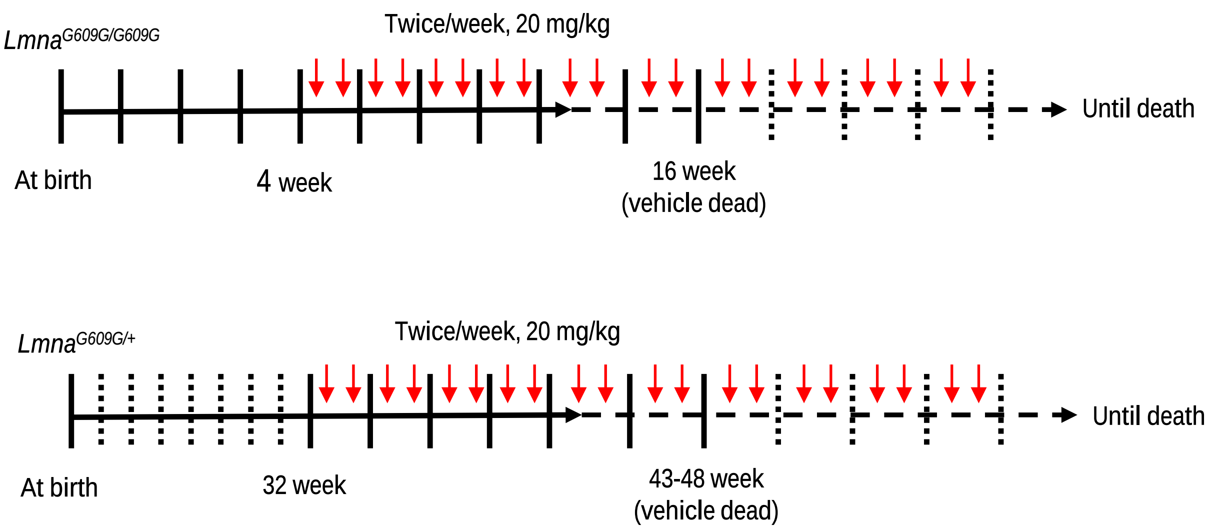

Fig. S8. Injection schedules for *Lmna*^{G609G} progerin model mice. *Lmna*$^{G609G/G609G}$ mice were injected with progerinin twice a week at a concentration of 20 mg/kg, starting at 5 weeks of age. *Lmna*$^{G609G/+}$ mice were injected with progerinin twice a week, starting at 32 weeks of age.

Figure S9

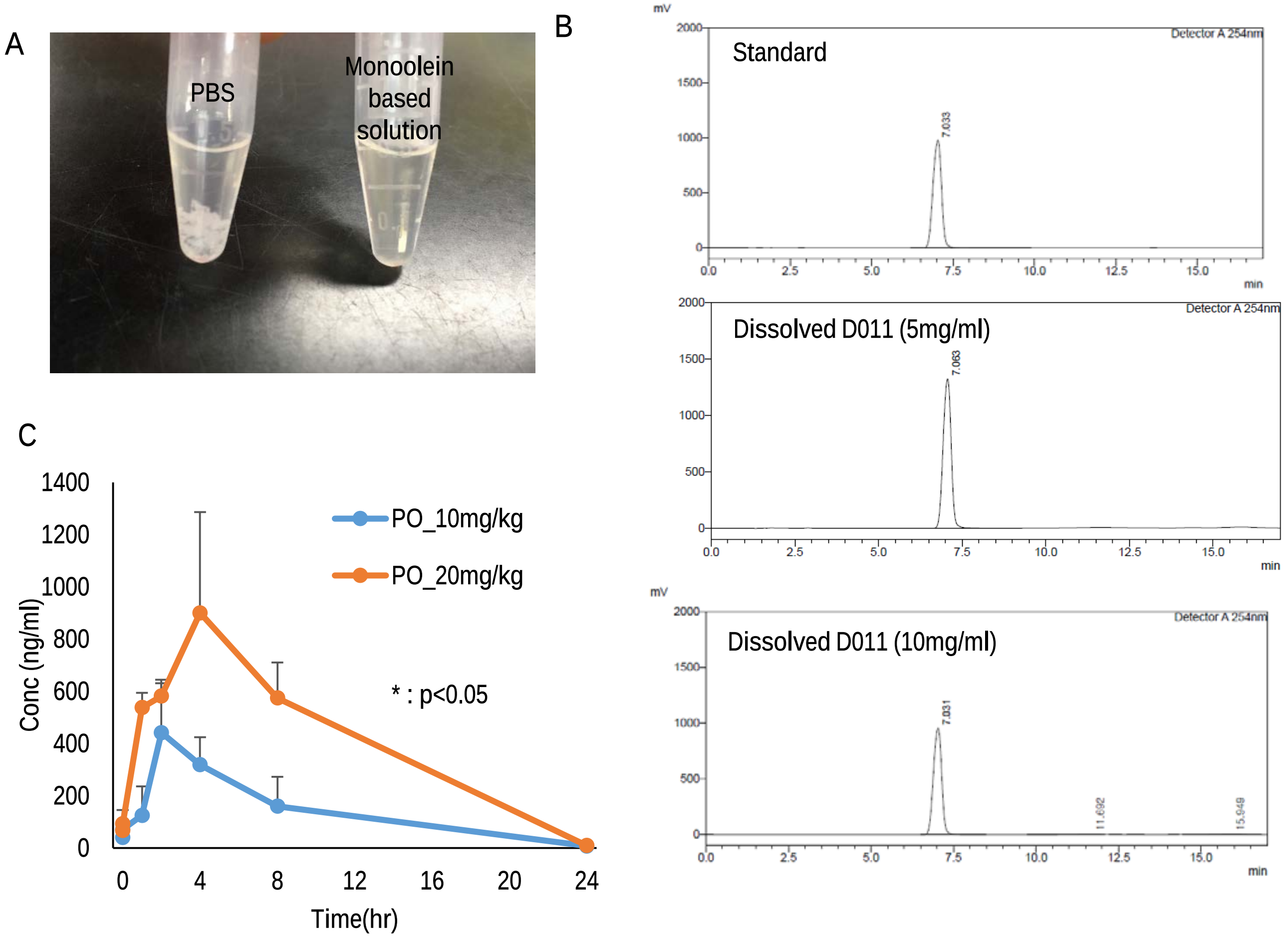

D

Plasma concentration after oral administration at a dose of 10mg/kg (n=3)

Time (hr)	Subject 1	Subject 2	Subject 3	Mean	SD
0.25	33.1	36.3	53.7	41	11.1
0.5	51.9	103.7	57.3	71	28.5
1	18	239.9	116.4	124.8	111.2
2	230.7	500	594.9	441.9	188.9
4	208	330.8	417.5	318.8	105.3
8	107	289.7	83.5	160.1	112.9
24	21.1	0.9	1	7.7	11.6

Plasma concentration after oral administration at a dose of 20mg/kg (n=3)

Time (hr)	Subject 1	Subject 2	Subject 3	Mean	SD
0.25	94.8	59.2	48	67.3	24.4
0.5	49	80	150.4	93.1	51.9
1	499.6	510.5	602.3	537.5	56.4
2	589.1	516	639.9	581.7	62.3
4	732.2	625.9	1341.7	899.9	386.2
8	529.4	727.4	465.3	574	136.6
24	13.6	14.1	1.4	9.7	7.2

Fig. S9. In vitro ADME results of SLC-D011 via oral administration. **A.** Formulation for complete dissolution. Due to its extremely hydrophobic property, SLC-D011 was not dissolved in water solution (left). To overcome this problem, several edible solutions were tested and monoolein-based solution was selected as a dissolving solution (right). **B.** SLC-D011 was very stable in solution. For complete dissolution, heating at 80℃ and sonication step were required. However, SLC-D011 was not broken despite of heating and sonication. The original SLC-D011 and dissolved one showed the same pattern in LC-MS. **C** and **D.** Pharmacokinetic analysis of SLC-D011 dissolved in monoolein-based solution.

Figure S10

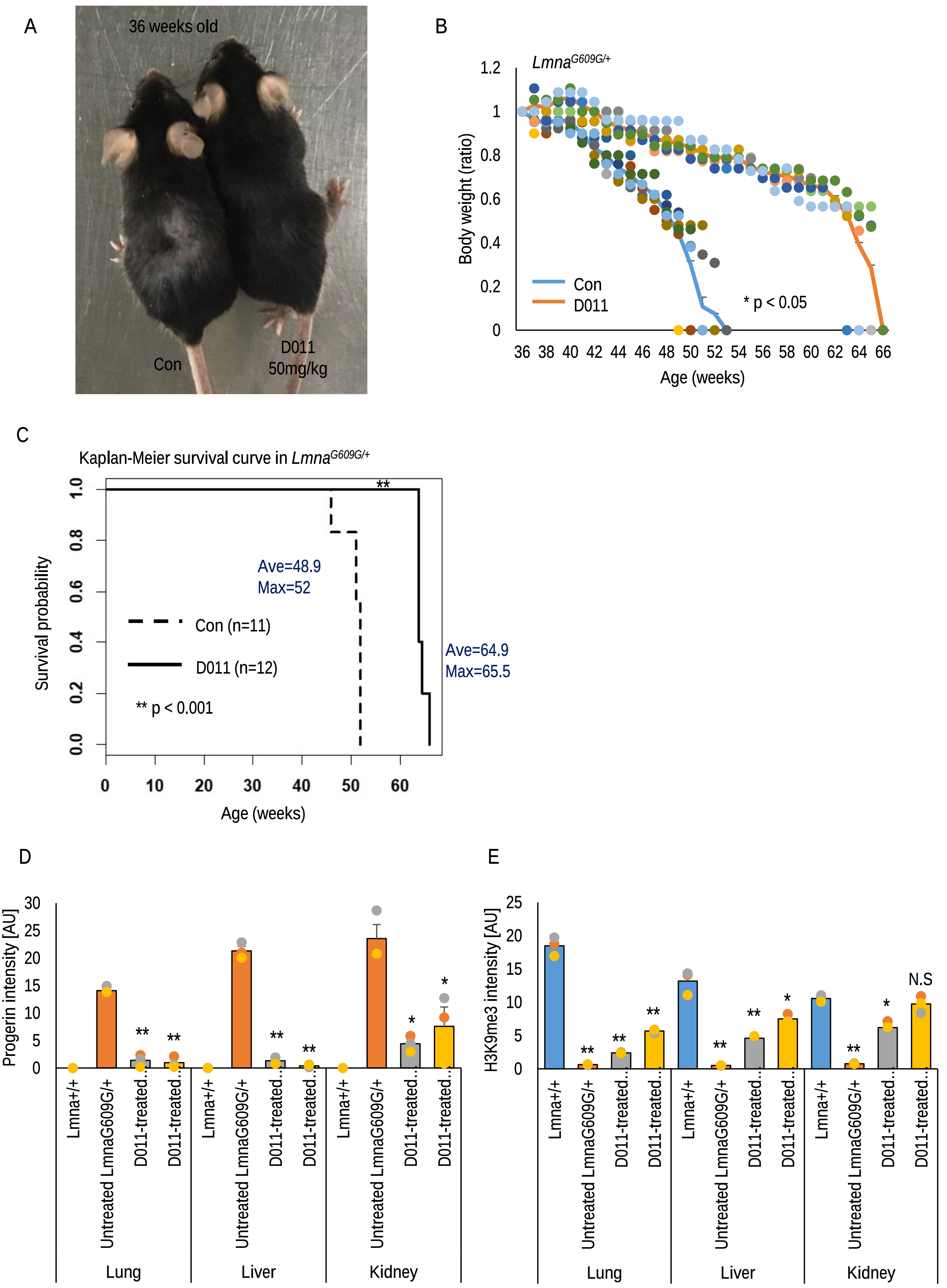

A
36 weeks old
Con
D011
50mg/kg
B
LmnaG609G/+
Body weight (ratio)
1.2
1
0.8
0.6
0.4
0.2
0
Con
D011
* p < 0.05
36 38 40 42 44 46 48 50 52 54 56 58 60 62 64 66
Age (weeks)
C
Kaplan-Meier survival curve in LmnaG609G/+
Survival probability
1.0
0.8
0.6
0.4
0.2
0.0
**
Ave=48.9
Max=52
Con (n=11)
D011 (n=12)
** p < 0.001
Ave=64.9
Max=65.5
0 10 20 30 40 50 60
Age (weeks)
D
Progerin intensity [AU]
30
25
20
15
10
5
0
Lmna+/+
Untreated LmnaG609G/+
D011-treated…
D011-treated…
Lung
Liver
Kidney
E
H3K9me3 intensity [AU]
25
20
15
10
5
0
N.S
Lmna+/+
Untreated LmnaG609G/+
D011-treated…
D011-treated…
Lung
Liver
Kidney

Fig. S10. Oral administration of SLC-D011 suppresses premature aging features of *Lmna*$^{G609G/+}$ mice. **A**. Gross morphology of SLC-D011 treated *Lmna*$^{G609G/+}$ mice at 36 weeks old. **B**. Oral administration of SLC-D011 increases body weights of *Lmna*$^{G609G/+}$ mice (untreated: n=11; SLC-D011: n=12), $*p<0.05$. **C.** Favorable effect of SLC-D011 on life span of *Lmna*$^{G609G/+}$ mice. Comparing to vehicle control (ave = 48.9 weeks and max = 52 weeks), SLC-D011 treatment could obviously extend the average life span to 64.9 weeks (max = 65.5 weeks). $**p < 0.005$. **D.** The bar graph shows the intensity of progerin levels in lung, liver, and kidney tissues of age-matched *Lmna*$^{+/+}$ mouse, untreated *Lamn*$^{G609G/+}$ mouse and two progerinin-treated *Lmna*$^{G609G/+}$ mice (n = 2 independent experiments; one Student's *t*-test). **E.** The bar graph shows the intensity of H3K9me3 levels in lung, liver, and kidney tissues of age-matched *Lmna*$^{+/+}$ mouse, untreated *Lamn*$^{G609G/+}$ mouse and two progerinin-treated *Lmna*$^{G609G/+}$ mice (n = 2 independent experiments; one Student's *t*-test), $**p < 0.001$, $*p < 0.05$, N.S: not significant. AU, arbitrary units.

Figure S11

A

Untreated *Lmna*$^{+/+}$ | Untreated *Lmna*$^{G609G/+}$ | D011 *Lmna*$^{G609G/+}$

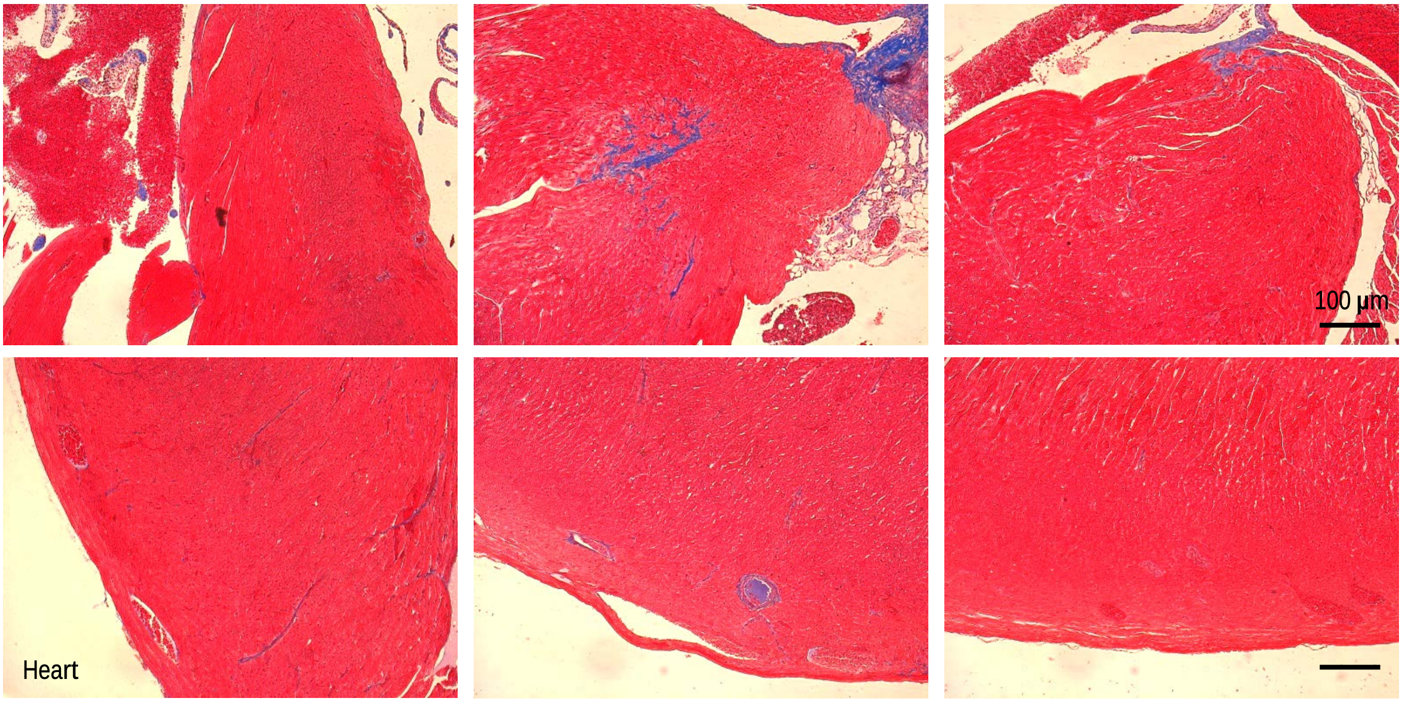

B

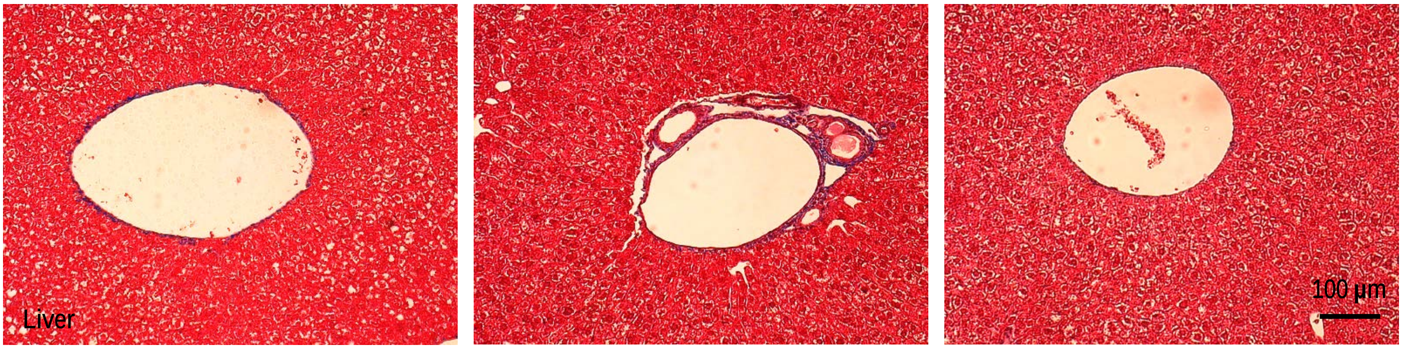

C

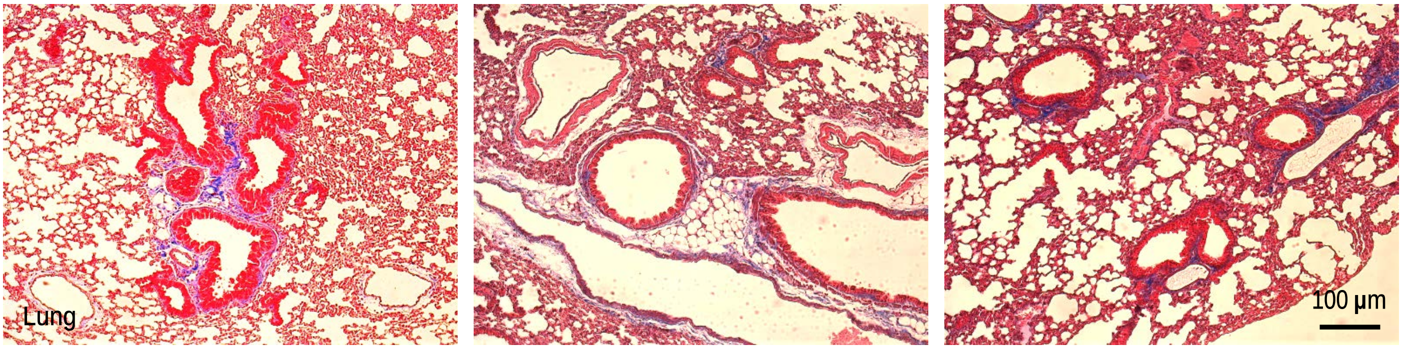

Figure S11 (continue)

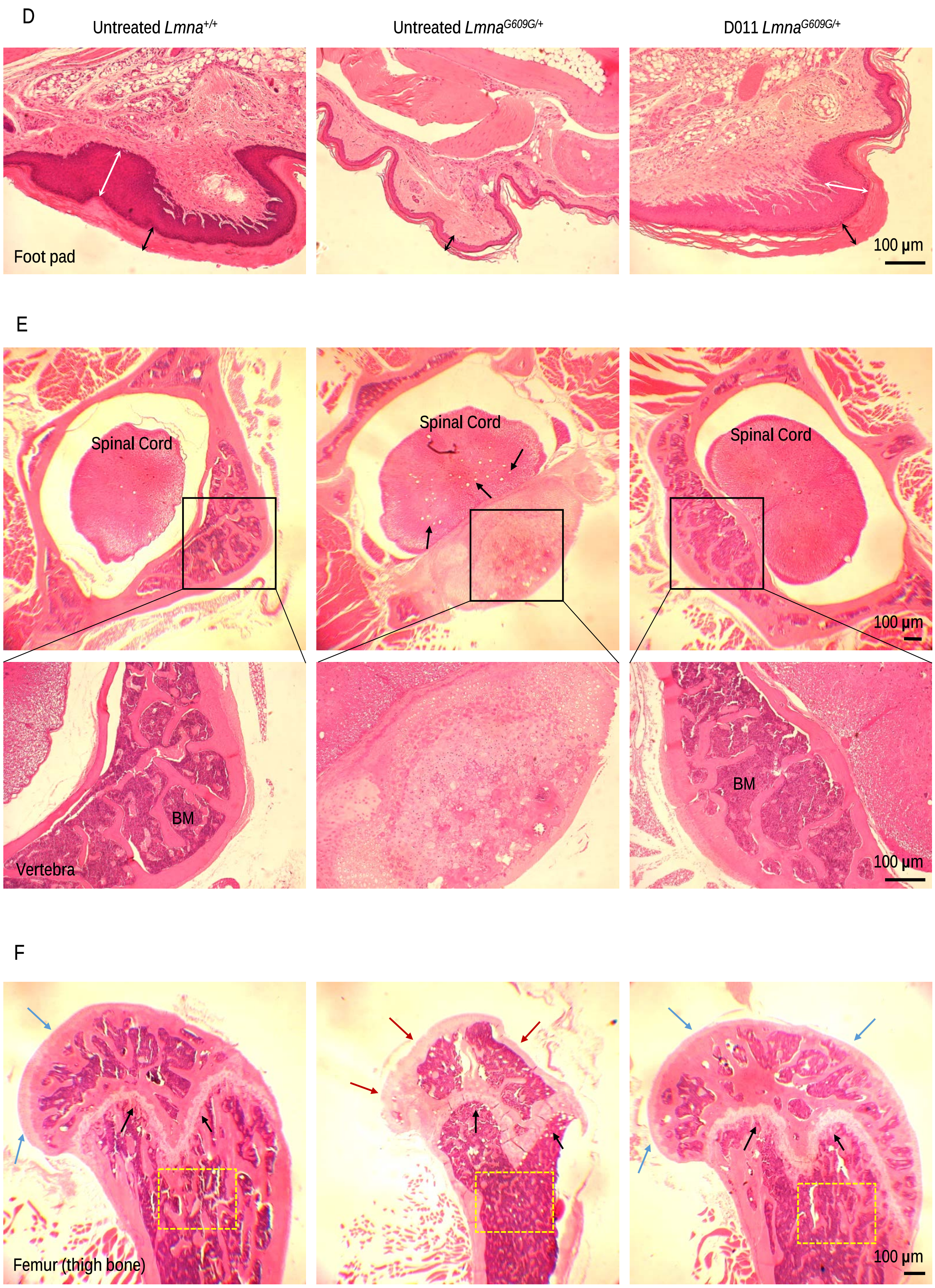

Figure S11 (continue)

G

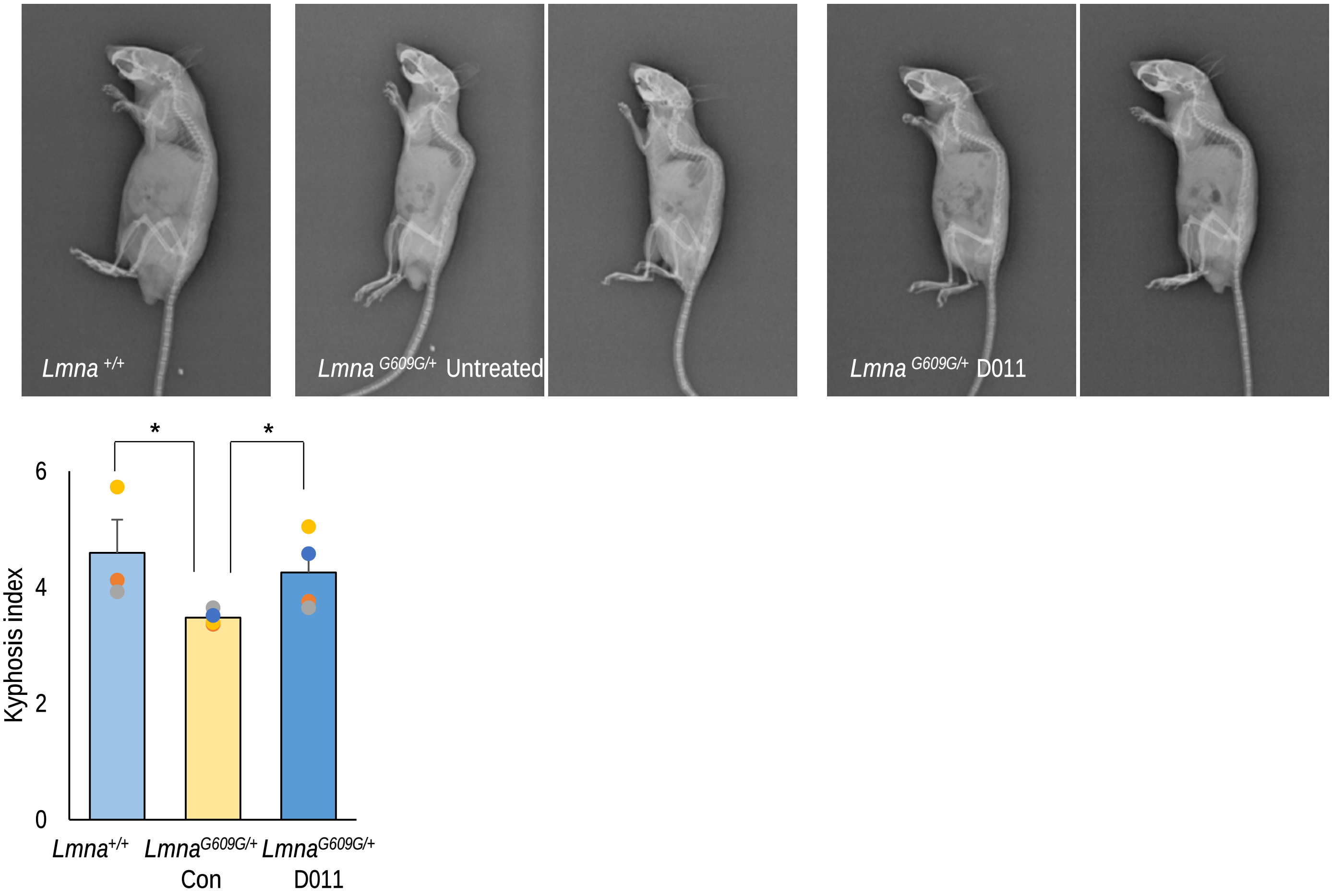

H

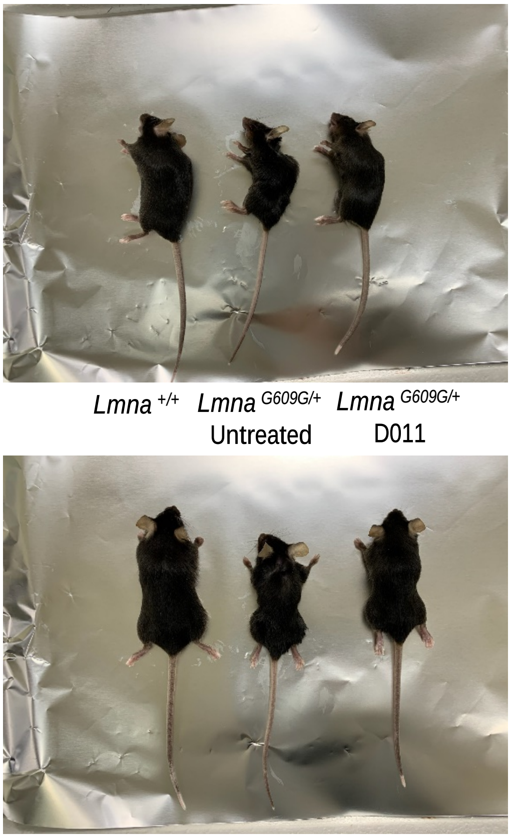

Fig. S11. Histological analysis of age-matched *Lmna*$^{+/+}$ mice (left), untreated *Lamn*$^{G609G/+}$ mice (middle) and progerinin-treated *Lmna*$^{G609G/+}$ mice (right, *Lmna*$^{+/+}$: n=4; untreated *Lmna*$^{G609G/+}$: n=6; progerin-treated *Lmna*$^{G609G/+}$: n=6). **A-C**. Masson Trichrome staining for fibrosis analysis was performed after 8-week treatment. **A.** Reduction of fibrosis in heart after administration of progeirnin in *Lmna*^{G609G} transgenic mice. The collagen-rich fibrotic area (blue) is widely distributed in untreated *Lmna*$^{G609G/+}$ mice compared to *Lmna*$^{+/+}$ and progerin-treated *Lmna*$^{G609G/+}$ mice. **B.** Fibrosis in liver of *Lmna*^{G609G} transgenic mice was reduced by progerinin. **C.** Untreated *Lmna*$^{G609G/+}$ mice show a relatively loose connection between blood vessel walls and tissues in lungs. **D-E.** Hematoxylin and Eosin (H&E) staining was performed to compare skin and bones of *Lmna*$^{+/+}$ and *Lmna*^{G609G} mice. Conditions of foot pad skin (**D**), vertebra (**E**), and femur (**F**) of *Lmna*$^{+/+}$ and progerin-treated *Lmna*$^{G609G/+}$ mice were better than those of untreated *Lmna*$^{G609G/+}$ mice. **D**. Cuticle layers and epidermis of foot pad skin were improved after treatment with progerinin. Black double-sided arrow indicates cuticle layers of food pad skin. White double-sided arrow indicates thickness of epidermis. **E**. Defects in bone marrow and spinal cord (black arrows indicate loss of spinal cord cells) of vertebrae of untreated *Lmna*$^{G609G/+}$ mice. Progerinin rescues the defects of vertebrae of *Lmna*^{G609G} transgenic mice. The figures below are enlarged square parts of the figures above. BM, bone marrow. **F**. Bone density and cell proliferation of femur were reduced in untreated *Lmna*$^{G609G/+}$ mice compare to *Lmna*$^{+/+}$ and progerin-treated *Lmna*$^{G609G/+}$ mice. Hyaline Cartilage of femur was worn out in untreated *Lmna*$^{G609G/+}$ mice (red arrows) compared to *Lmna*$^{+/+}$ and progerin-treated *Lmna*$^{G609G/+}$ mice (blue arrows). The growth plates (black arrows) harden into solid bone in untreated *Lmna*$^{G609G/+}$ mice. Loss of trabecular bone (yellow dotted square parts) loss was restored by treatment with progerinin. **G**. X-ray lateral projection of *Lmna*$^{+/+}$ and *Lmna*^{G609G} transgenic mice at 41 weeks of age. Abundant adipose tissues are present in abdomens of *Lmna*$^{+/+}$ and progerin-treated *Lmna*$^{G609G/+}$ mice compared to untreated *Lmna*$^{G609G/+}$ mice. Kyphosis Index (KI) index was accessed after 8-week treatment. The bar graph shows KI assessment (ref. 30) for *Lmna*$^{+/+}$, untreated *Lmna*$^{G609G/+}$, and D011-treated *Lmna*$^{G609G/+}$ mice at 41 weeks of age (unpaired *t*-test), *$p < 0.05$. Severity of kyphosis depends on genotype and treatment of SLC-D011. **H.** Gross morphology of age-

matched *Lmna*$^{+/+}$ mouse, untreated *Lamn*$^{G609G/+}$ mouse, and progerinin-treated *Lmna*$^{G609G/+}$ mice.

Figure S12

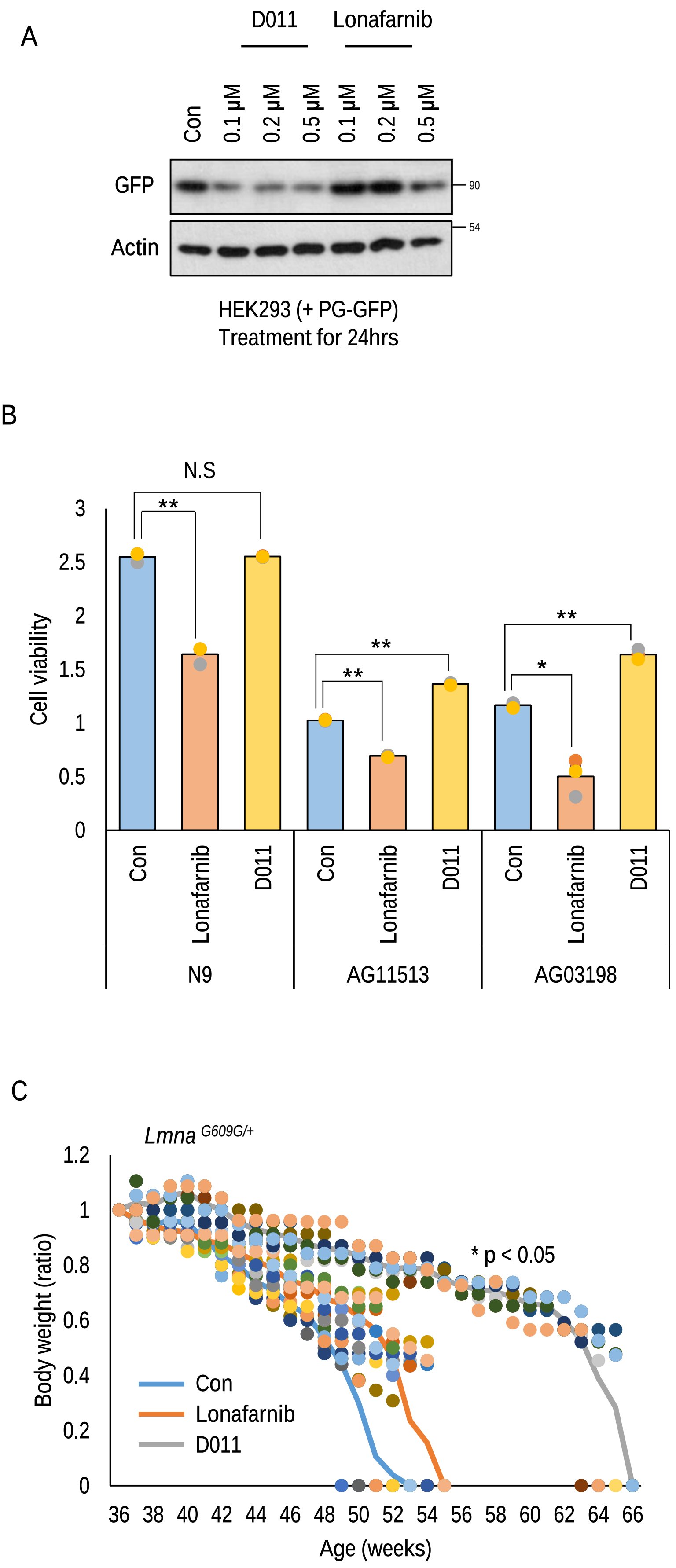
A
D011
Lonafarnib
Con
0.1 µM
0.2 µM
0.5 µM
0.1 µM
0.2 µM
0.5 µM
GFP
Actin
90
54
HEK293 (+ PG-GFP)
Treatment for 24hrs
B
N.S
**
**
**
**
*
Cell viability
3
2.5
2
1.5
1
0.5
0
Con
Lonafarnib
D011
Con
Lonafarnib
D011
Con
Lonafarnib
D011
N9
AG11513
AG03198
C
Lmna G609G/+
1.2
1
0.8
0.6
0.4
0.2
0
Body weight (ratio)
* p < 0.05
Con
Lonafarnib
D011
36 38 40 42 44 46 48 50 52 54 56 58 60 62 64 66
Age (weeks)

Figure S12 (continue)

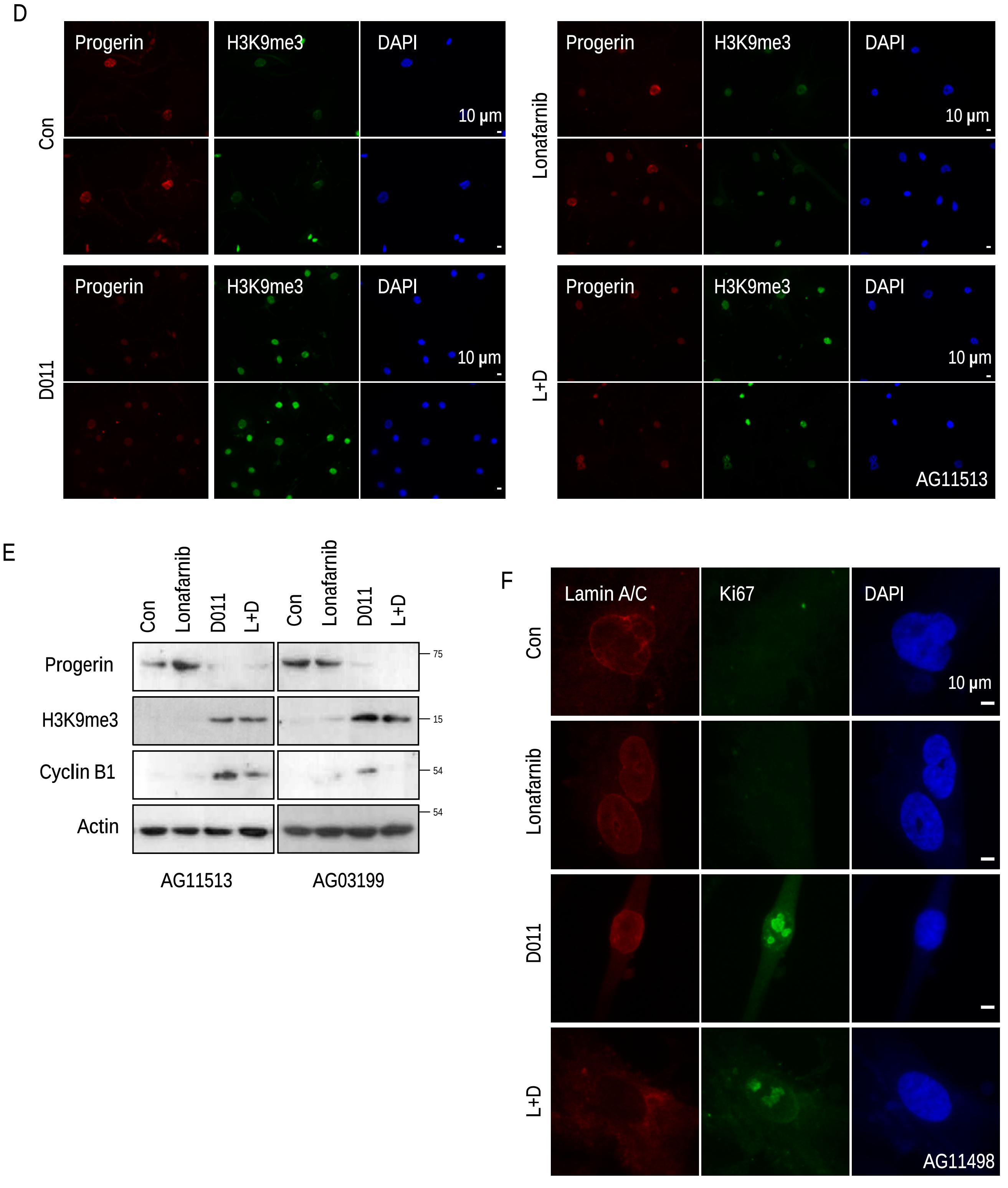

Fig. S12. Comparative study of lonafarnib and SLC-D011. **A**. SLC-D011, compared to lonafarnib, reduces exogenous progerin expression in HEK293 cells. **B**. Long-term treatment (treated with 2μM every other day for 2 weeks) with lonafarnib or SLC-D011 was performed for normal fibroblasts and HGPS cells. Long-term treatment induced cell death in both normal fibroblasts and HGPS cells. MTT assay was used to determine the cellular viability after treatment with lonafarnib or SLC-D011 (n = 3 independent experiment; two-tailed Student's t-test), $^{**}p < 0.001$, $^{*}p < 0.05$, N.S: not significant. Data are presented as mean ± SD. **C**. Oral administration of SLC-D011 maintains body weights of $Lmna^{G609G/+}$ mice much longer than that of lonafarnib by daily oral administration at a concentration of 50 mg/kg. $Lmna^{G609G/+}$ mice were daily administrated with chemical-mixture diets, starting at 32 weeks of age (untreated: n=11; SLC-D011: n=12; lonafarnib: n=10). **D.** Single treatment of SLC-D011 and combination treatment of SLC-D011 and lonafarnib for 3 days induce the expression of H3K9me3 in HGPS cells (AG11513) compared to lonafarnib single-treatment or control. **E.** Single treatment and combination treatment of SLC-D011 (500 nM) and lonafarnib (500 nM) were performed in HGPS cells for 2 weeks. Lonafarnib interrupted the induction of cyclin B1 expression by SLC-D011 after long-term combination treatment. **F.** SLC-D011 induces the expression of Ki67 and ameliorates nuclear deformation in HGPS cells (AG11498) at 7 days after treatment. Combination treatment of SLC-D011 and lonafarnib also increased Ki67-positive nuclei, but less than single-treatment with SLC-D011. There was no significant difference in the number of Ki67-positive nuclei between single-treatment with lonafarnib and control. Lonafarnib-treated HGPS cells showed donut-shaped nuclei.

Fig. 1b

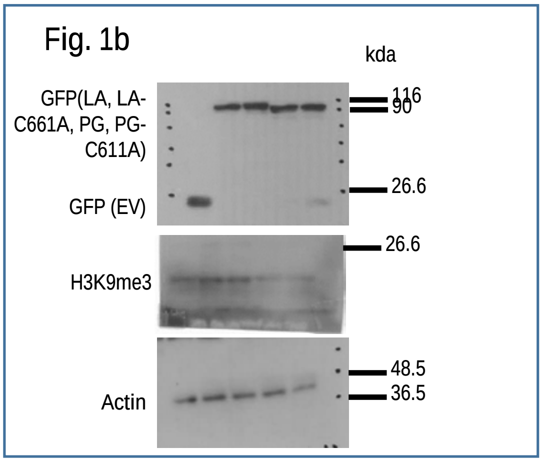

Fig. 1c

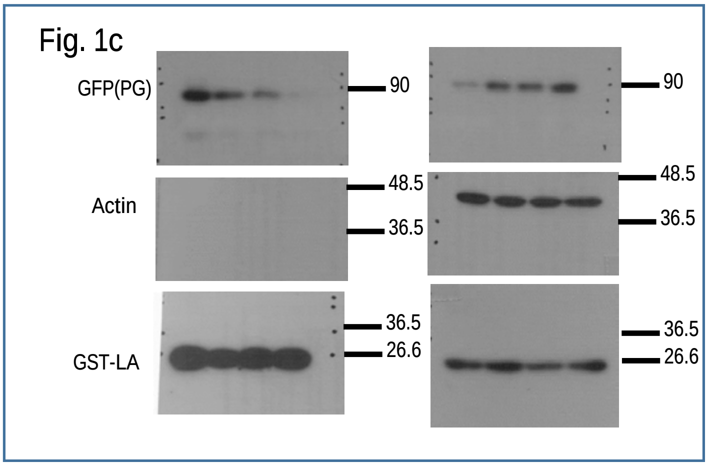

Fig. 1e

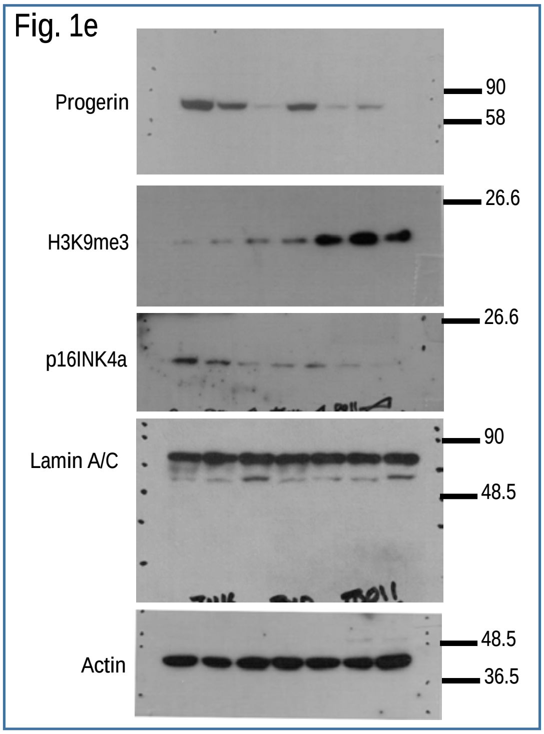

Fig. 1f

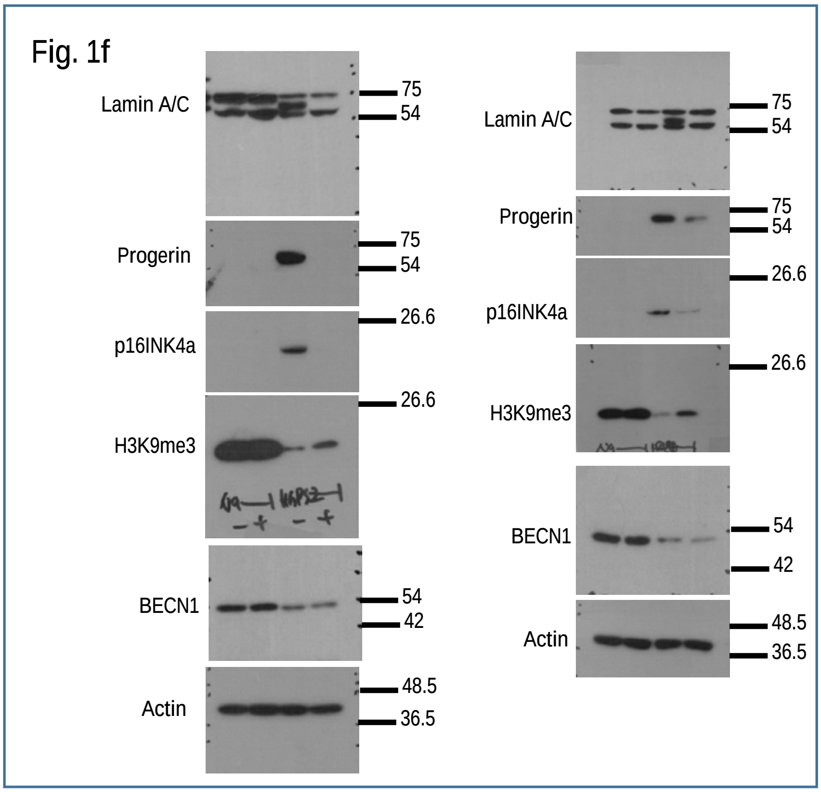

Fig. 2d

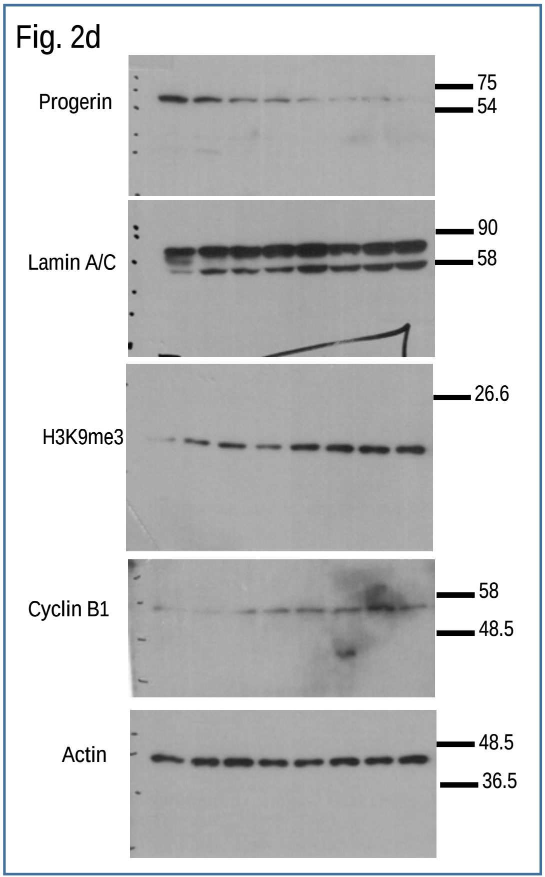

Fig. 2e

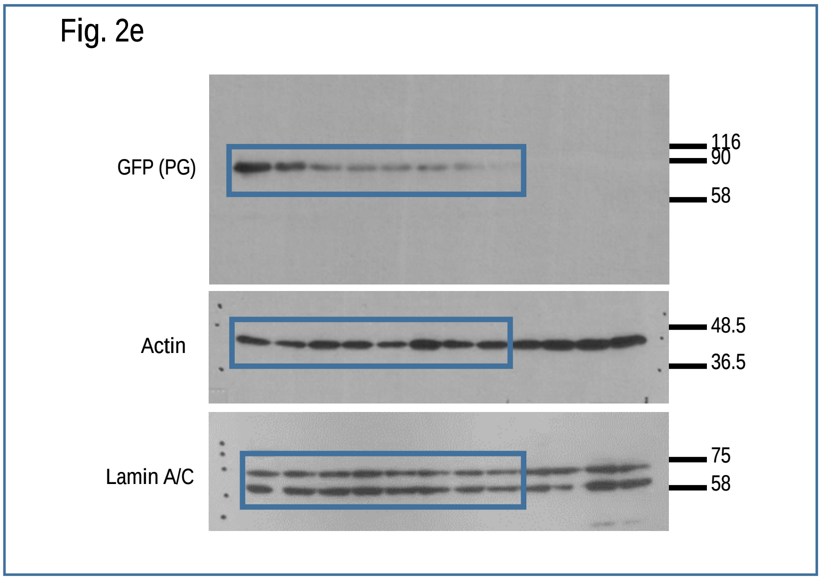

Fig. 4d

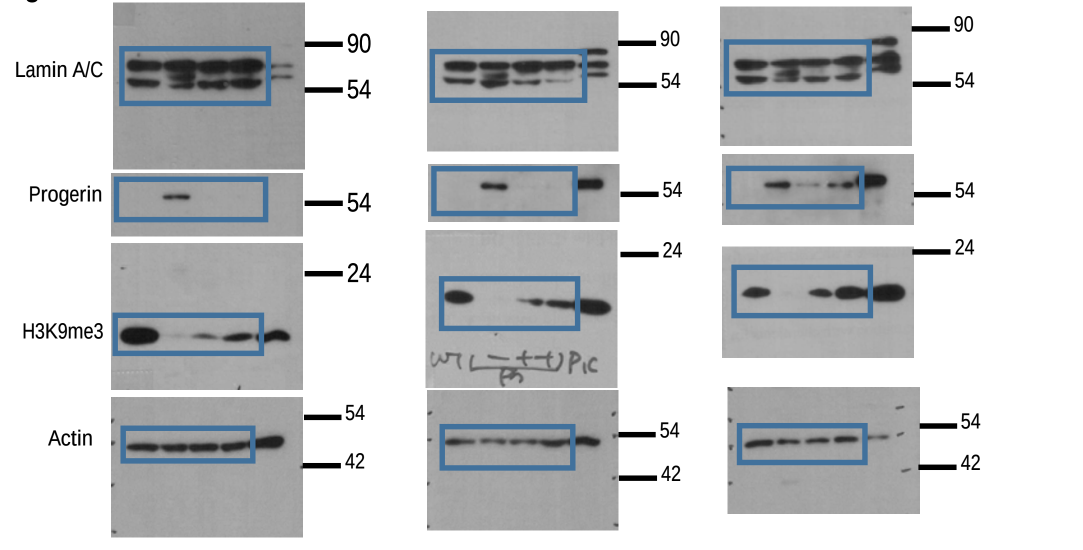

Fig. 4i

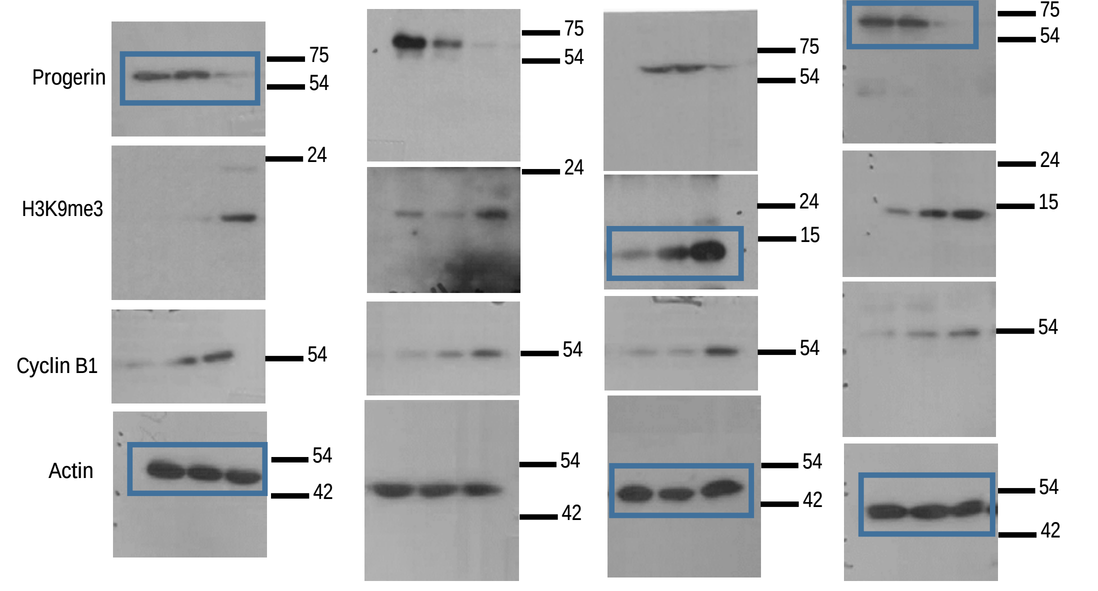

Fig. S1d

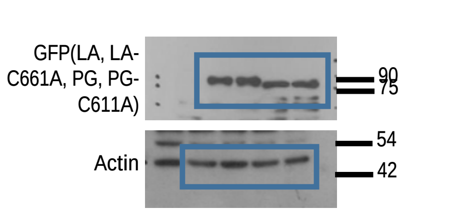

Fig. S1g

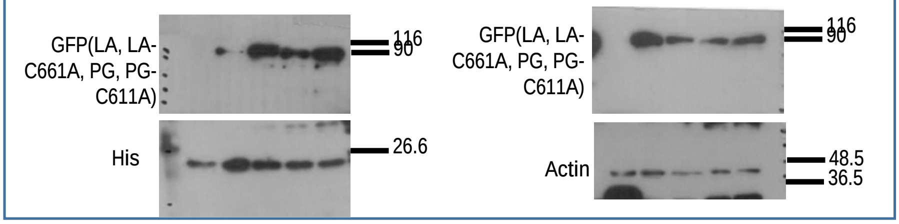

Fig. S3b

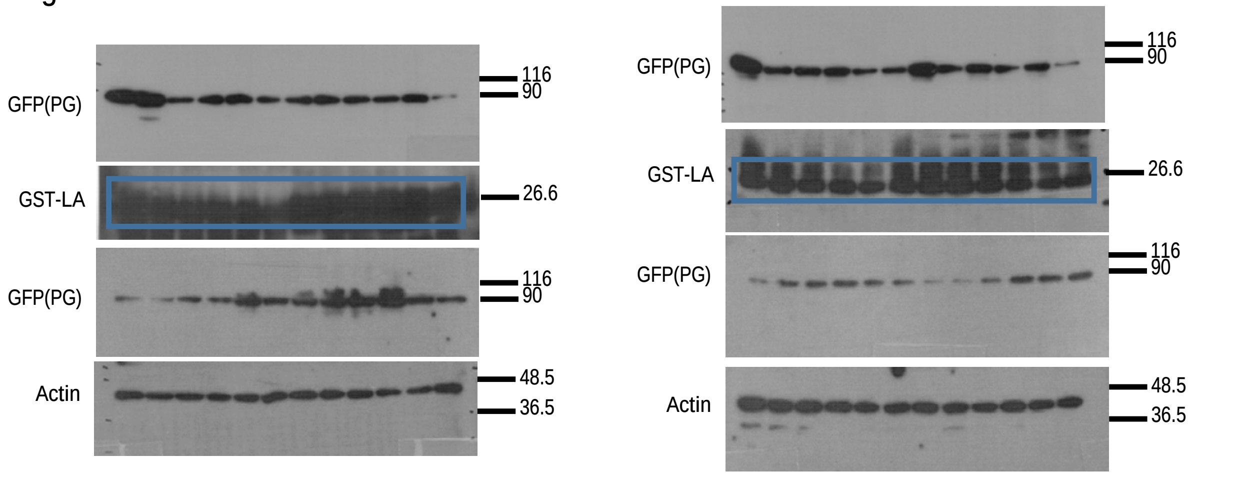

Fig. S3c

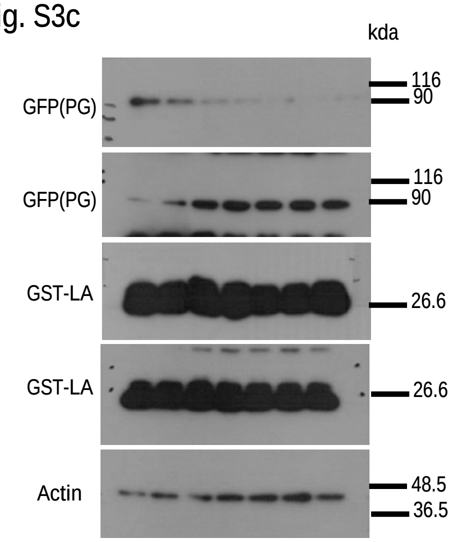

Fig. S3d

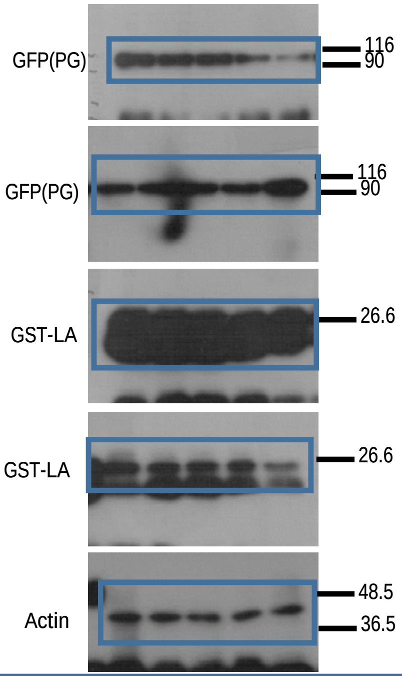

Fig. S3j

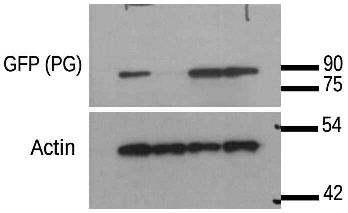

Fig. S3k

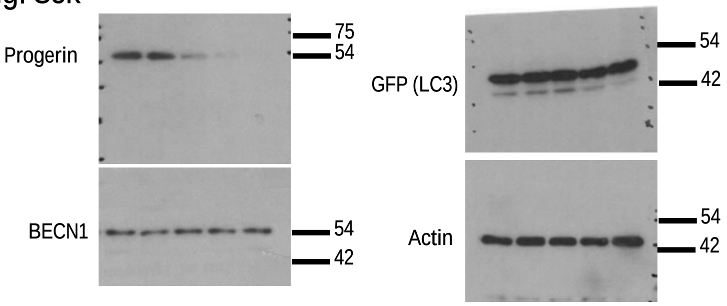

Fig. S3l

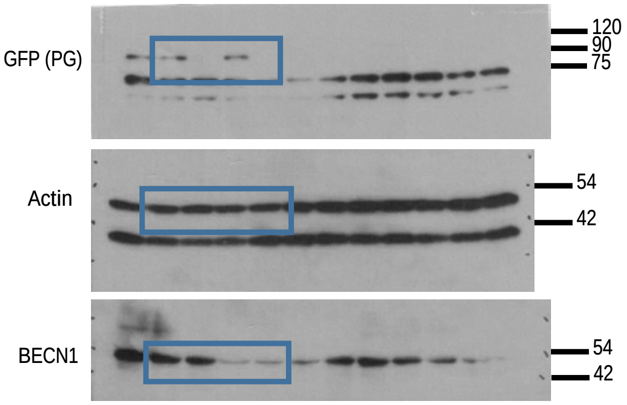

Fig. S7a

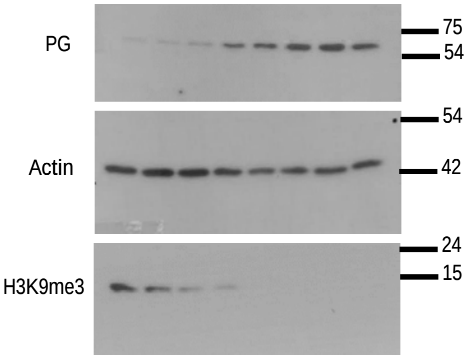

Fig. S7b

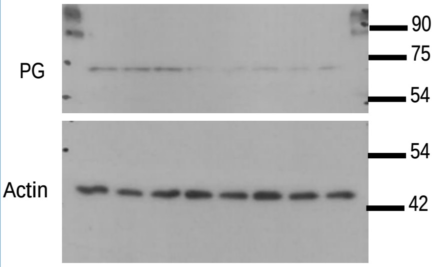

Fig. S12a

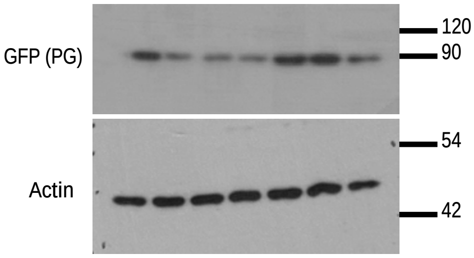

Fig. S12d

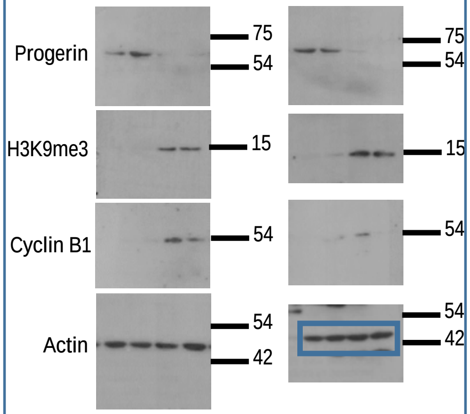

Supplement: Supplementary file 2 — Supplementary Information [file 42003_2020_1540_MOESM2_ESM.pdf]
