# Supplementary material for: Progerinin, an optimized progerin-lamin A binding inhibitor, ameliorates premature senescence phenotypes of Hutchinson-Gilford progeria syndrome
Source: Commun Biol. 2021 Jan 4;4:5. doi: 10.1038/s42003-020-01540-w (PMC7782499; doi:10.1038/s42003-020-01540-w)
Supplement: Supplementary file 3 — Description of Additional Supplementary Files [file 42003_2020_1540_MOESM3_ESM.docx]

**Description of Additional Supplementary Files**

File Name: Supplementary Data1

Description:

Toxicity analysis of SLC-D011 in rats and dogs

Page 1-49. A single oral gavage dose toxicity study in SD rats

Page 50-72. A maximum tolerated dose study in beagle dogs following escalated-dose oral administration

File Name: Supplementary Data2

Description:

Characterization data of SLC-D011

Fig1. Synthetic scheme of SLC-D011

Tab1. ^1^H-NMR chemical shift and assignment of SLC-D011

Fig2. ^1^H-NMR spectrum of SLC-D011

Tab2. ^13^C-NMR chemical shift and assignment of SLC-D011

Fig3. ^13^C-NMR spectrum of SLC-D011

Fig4. Mass spectrum of SLC-D011

Fig5. X-ray Diffractometry

File Name: Supplementary Data3

Description: Source data underlying plots shown in figures.

Fig1G. Rate of cell proliferation after treatment with chemicals in AG03198

Fig1H. Rate of nuclear deformation after treatment with chemicals in AG03198

Fig2F. Rate of Ki67-positive cells after treatment with SLC-D011 in AG11498

Fig3B. Changes of body weight after IP injection with SLC-D011 in *Lmna^G609G/G609G^* mice

Fig3E. Changes of body weight after IP injection with SLC-D011 in *Lmna^G609G/+^* mice

Fig4B. Changes of body weight after oral administration with SLC-D011 in *Lmna^G609G/G609G^* mice

Fig4E. Grip strength (Force, %)

Fig4F. Heart rate (bmp)

FigS1D. Population of GFP-positive cells and relative expression of GFP

FigS1E. Percentage of deformed nuclei in GFP-positive cells

FigS1F. Rate of GFP expression

FigS1G. Rate of H3K9me3 expression

FigS3F. Intensity of H3K9me3 after treatment with chemicals in AG03198

FigS3G. Intensity of progerin after treatment with chemicals in AG03198

FigS3H. Intensity of progerin after treatment with SLC-D011

FigS4A. Counts of cell population after treatment with chemicals in AG11513

FigS4C. Cell proliferation after treatment with SLC-D011

FigS4D. H3K9me3-positive cells after treatment with chemicals in AG03198

FigS7C. Intensity of progerin after treatment with SLC-D011

FigS7D. Intensity of GFP after treatment with SLC-D011

FigS7E. Rate of Ki67-positive cells after treatment with SLC-D011 in AG03199

FigS7G. Counts of cell population after treatment with SLC-011 in AG03198

FigS7H. Percentage of nuclear deformation after treatment with SLC-D011 in AG03198

FigS10B. Changes of body weight after oral administration with SLC-D011 in *Lmna^G609G/+^* mice

FigS10D. Intensity of progerin after treatment with SLC-D011 in *Lmna^G609G/+^* mice

FigS10E. Intensity of H3K9me3 after treatment with SLC-D011 in *Lmna^G609G/+^* mice

FisS11G. Kyphosis index

FigS12B. Rate of cell viability after treatment with lonafarnib or SLC-D011

FigS12C. Changes of body weight after oral administration with lonafarnib or SLC-D011 in *Lmna^G609G/+^* mice
