# Supplementary material for: Progerinin, an optimized progerin-lamin A binding inhibitor, ameliorates premature senescence phenotypes of Hutchinson-Gilford progeria syndrome
Source: Commun Biol. 2021 Jan 4;4:5. doi: 10.1038/s42003-020-01540-w (PMC7782499; doi:10.1038/s42003-020-01540-w)
Supplement: Supplementary file 4 — Supplementary Data 1 [file 42003_2020_1540_MOESM4_ESM.pdf]

FINAL REPORT

SLC-D011:
A SINGLE ORAL GAVAGE DOSE TOXICITY STUDY
IN SD RATS

STUDY NUMBER: QT18122

NONCLNICAL RESEARCH CENTER
QUBEST BIO CO., LTD.

www.QuBEST.co.kr TEL 031-706-2995 FAX 031-706-2996 E-mail info@qubest.co.kr
The BEST Partner for Your Successful Drug Development

STUDY DIRECTOR STATEMENT

STUDY TITLE: SLC-D011: A SINGLE ORAL GAVAGE DOSE TOXICITY STUDY IN SD RATS

STUDY NUMBER: QT18122

I, the undersigned, hereby declare that the work described in this report was performed under my supervision, as a Study Director, and that the report provides a true and accurate record of the results obtained.

The study was performed in accordance with Standard Operation Procedures for nonclinical studies. The test article information such as identity, strength, purity, composition or other characteristics is the responsibility of the Sponsor.

TESTING FACILITY Room 1304, U-TOWER, 120 Heungdeokjungang-ro, Giheung-gu, Yongin-si Gyeonggi-do, 16950, Korea

TEL +82-31-706-2995, FAX +82-31-706-2996, Homepage: www.qubest.co.kr

Nonclinical Research Center, QuBEST BIO Co., Ltd.

#301, Daewoo Frontier Valley I, 16-25, Dongbaekjungang-ro 16beon-gil, Giheung-gu, Yongin-si, Gyeonggi-do, 17015, Republic of Korea

STUDY DIRECTOR SANGBUM WON, MS ______ Nov, 10, 2018

MANAGEMENT SOOHYEON KIM, MS ______ NOV / 10 / 2018

TABLE OF CONTENTS

STUDY DIRECTOR STATEMENT 2
TABLE OF CONTENTS 3
1. SUMMARY 6
2. STUDY INTRODUCTION 7
2.1 STUDY TITLE 7
2.2 STUDY OBJECTIVE 7
2.3 REGULATORY GUIDELINES 7
2.4 SPONSOR REPRESENTATIVE 7
2.5 STUDY SCHEDULE 7
3. TEST ARTICLE AND VEHICLE 7
3.1 TEST ARTICLE 7
3.2 VEHICLE 8
4. ANIMALS AND HUSBANDRY 8
5. EXPERIMENTAL PROCEDURES 9
5.1 PREPARATION OF DOSE FORMULATIONS 9
5.2 ANALYSIS OF DOSE FORMULATIONS 9
5.3 JUSTIFICATION OF ADMINISTRATION ROUTE 10
5.4 GROUP ASSIGNMENT AND DOSE LEVEL 10
5.5 METHOD OF ADMINISTRATION 10
6. OBSERVATIONS 10
6.1 MORTALITY 10
6.2 CLINICAL OBSERVATION 10
6.3 BODY WEIGHTS 10
6.4 GROSS NECROPSY 11
7. STATISTICAL ANALYSES 11

8. RESULTS AND DISCUSSION 11
8.1 MORTALITY AND CLINICAL OBSERVATION 11
8.2 BODY WEIGHTS 11
8.3 GROSS NECROPSY 11
9. CONCLUSION 11
10. FIGURES 12
FIGURE 1. BODY WEIGHT IN MALE RATS (GROUP SUMMARY) 13
FIGURE 2. BODY WEIGHT IN FEMALE RATS (GROUP SUMMARY) 13
FIGURE 3. BODY WEIGHT GAIN IN MALE RATS (GROUP SUMMARY) 14
FIGURE 4. BODY WEIGHT GAIN IN FEMALE RATS (GROUP SUMMARY) 14
11. TABLES 15
TABLE 1. MORTALITY IN MALE RATS (SUMMARY) 16
TABLE 2. MORTALITY IN FEMALE RATS (GROUP SUMMARY) 17
TABLE 3. CLINICAL OBSERVATION IN RATS (GROUP SUMMARY) 18
TABLE 4. BODY WEIGHT AND BODY WEIGHT GAIN IN MALE RATS (GROUP SUMMARY) 19
TABLE 5. BODY WEIGHT AND BODY WEIGHT GAIN IN FEMALE RATS (GROUP SUMMARY) 20
TABLE 6. GROSS NECROPSY FINDING IN RATS (GROUP SUMMARY) 21
12. APPENDICES 22
APPENDIX 1. CLINICAL OBSERVATION IN MALE RATS (INDIVIDUAL) 23
APPENDIX 2. CLINICAL OBSERVATION IN FEMALE RATS (INDIVIDUAL) 24
APPENDIX 3. BODY WEIGHT AND BODY WEIGHT GAIN IN MALE RATS (INDIVIDUAL) 25
APPENDIX 4. BODY WEIGHT AND BODY WEIGHT GAIN IN FEMALE RATS (INDIVIDUAL) 26
APPENDIX 5. GROSS NECROPSY FINDING IN MALE RATS (INDIVIDUAL) 27
APPENDIX 6. GROSS NECROPSY FINDINGS IN FEMALE RATS (INDIVIDUAL) 28
APPENDIX 7. PROTOCOL 29
13. ANNEXES 39
ANNEX 1. CERTIFICATE OF ANALYSIS (COA) 40
ANNEX 2. ANIMAL HEALTH MONITORING REPORT 41
ANNEX 3. CERTIFICATE OF BEDDING GAMMA IRRADIATION 44
ANNEX 4. BEDDING ANALYSIS REPORT 45

ANNEX 5. CERTIFICATE OF FEED GAMMA IRRADIATION 47

ANNEX 6. FEED ANALYSIS RESULT 48

ANNEX 7. TAP WATER ANALYSIS RESULT 49

1. SUMMARY

Objective

The objective of this study was to investigate the potential acute toxicity or approximate lethal dose of SLC-D011 following a single oral gavage administration to the SD rats.

Study Group

The study group is detailed in Text Table 1.

Text Table 1. Study Group

Group	Treatment	Dose Level (mg/kg)	Dose Conc. (mg/mL)	No. of Animals	
				Toxicity	
				Males	Females
G1	Vehicle	0	0	3	3
G2	SLC-D011	500	50	3	3
G3	SLC-D011	2,000	200	3	3

Parameter Evaluated:

The following parameters were evaluated: mortality (twice daily), clinical signs (1, 2, and 4 hours post-dose on dosing day, and once daily during 7-day period), body weights (on Days 1, 4 and 7), and gross necropsy on Day 8.

Mortality and Clinical Observation

All animals survived to the scheduled necropsy. There were no clinical abnormalities in all study animals.

Body Weights

There were no test article-related effects on body weight in all test article-treated groups compared to the vehicle control group.

Gross Necropsy

There were no toxicologically significant gross lesions in all test article-treated groups. Internal gross necropsy findings such as small thymus and enlarged adrenal glands were observed in only one male of 500 mg/kg group (animal No. M7). However, the toxicological signigicance of this change was not determined due to the lack of histologic examination.

Based on these results, Sprague-Dawley rats given single oral gavage administration of SLC-D011 at 500 and 2,000 mg/kg were well tolerated. Consequently, approximate lethal dose (ALD) of SLC-D011 is considered to be > 2,000 mg/kg of both sexes.

2. STUDY INTRODUCTION

2.1 STUDY TITLE

SLC-D011: A SINGLE ORAL GAVAGE DOSE TOXICITY STUDY IN SD RATS

2.2 STUDY OBJECTIVE

The objective of this study was to investigate the potential acute toxicity or approximate lethal dose of SLC-D011 following a single oral gavage administration to the SD rats.

2.3 REGULATORY GUIDELINES

No specific regulatory guidelines. This study was conducted according to study-specific protocol after consultation with the Sponsor.

2.4 SPONSOR REPRESENTATIVE

PRG S&T Co., Ltd. / Minju Kim, PhD / Phone: 051-510-7562 / E-mail: rlaals09@prgst.com / Room 306, Hyowon Industry-Cooperation Building, Pusan National University, 2, Busandaehak-ro 63beon-gil, Geumjeong-gu, Busan, 46241, Republic of Korea

2.5 STUDY SCHEDULE

Animal receipt:	Aug 20, 2018
Administration:	Aug 26, 2018
Necropsy:	Sep 2, 2018
Final report:	Nov 10, 2018

3. TEST ARTICLE AND VEHICLE

3.1 TEST ARTICLE

Identity:	SLC-D011
Batch/Lot Number:	A05064-013S2
Appearance:	White powder
Purity:	99.2% (by HPLC)
Storage conditions:	Keep container tightly closed to avoid light in deep freezer (-70℃)
Handling precautions:	Routine protection procedures with gloves, goggles etc. and avoid contact with skin, eye and inhalation of vapor/mist

The test article was provided by the Sponsor (Manufacturer: Pharmaron (Ningbo), Inc. under non-GLP/non-GMP condition). Additional information related to the test articles involved was provided by the Sponsor. The remaining test article was retained at test facility for future related studies.

3.2 VEHICLE

Identity:	Oil based solution (Monoolein : Tricaprylin = 2 : 1)
Supplier:	Nonclinical Research Center, QuBEST BIO Co., Ltd.
Appearance:	Clear colorless liquid
Storage conditions:	Room temperature

1) **VEHICLE COMPONENT** 1

Identity:	Monoolein
Batch/Lot number:	RRNFJAT
Supplier:	TCI (Tokyo Chemical Industry Co., LTD.)
Appearance:	Clear colorless liquid
Storage conditions:	Room temperature

2) **VEHICLE COMPONENT** 2

Identity:	Tricaprylin
Batch/Lot number:	KEWXE-AL
Supplier:	TCI (Tokyo Chemical Industry Co., LTD.)
Appearance:	Clear colorless liquid
Storage conditions:	Room temperature

4. ANIMALS AND HUSBANDRY

Eleven (11/sex) specific pathogen-free Sprague-Dawley rats (approximately 6 weeks old) were obtained from SAMTAKO Ltd. (Osan, Korea) and nine (9/sex) rats were used for the study.

Immediately after receipt, animals were identified by temporary numbers by tail marking. Individual body weight was measured and their general health condition was examined by authorized study personnel. The rats were acclimated to the laboratory conditions for 6 days in Animal Room #104. During the acclimation period, rats were observed for general condition at least once daily and housed and cared in a same procedure as in study period. Prior to the start of the study, the animals with apparent good health were released for the study use and randomly assigned to 3 study groups based on the body weights. Remaining animals were kept in the study rooms and transferred to stock colony after the completion of in-life phase.

The animals were individually housed in wire bottom cages [220W x 300D x 180H (mm)] in Animal Room #104 of Nonclinical Research Center, QuBEST BIO Co., Ltd. All animals were housed throughout the acclimation period and during the study in an environmentally controlled room. The room temperature and humidity controls were set to maintain environmental conditions of 22±3℃ and 50±20%, respectively. Fluorescent lighting provided illumination for a 12-hour light (06:00 hours to

18:00 hours)/12-hour dark photoperiod. The 12-hour light/12-hour dark photoperiod was interrupted as necessary to allow the performance of protocol-specified activities. Air control units were set to provide a minimum of 10 fresh air changes per hour. During the study, animal room environment conditions were controlled within the target ranges.

Paper under the wire cages was changed every day and water bottles were changed twice a week. A certified and irradiated rodent diet (Purina Rodent Chow 38057, Cargill Inc., Repbulic of Korea) and tap water via polycarbonate bottles were provided *ad libitum*, except during designated procedures. Maximum allowable concentrations of contaminants in the diet were controlled and routinely analyzed by the manufacturers. The results of the analysis are retained at the testing facility. Periodic analysis of the water was subcontracted to management authorized analytical laboratories (Waters life-environment laboratory., Ltd. 17, Gosan-ro 148 beon-gil, Gunpo-si, Gyeonggi-do, Repbulic of Korea) and the analytical results are retained in the archives of the testing facility. It was considered that there were no known contaminants in the dietary materials that could have interfered with the objectives of the study.

During the study, each animal was identified by tail marking method and a cage label card displaying the study number, group, and animal number.

5. EXPERIMENTAL PROCEDURES

5.1 Preparation of Dose Formulations

The dose formulations were freshly prepared in clean bench prior to dosing according to Sponsor's provided mixing procedure.

Monoolein based solution (Monoolein : Tricaprylin = 2 : 1) for PO

Pre-heated the monoolein based solution to a temperature above 80℃. Added pre-heating the monoolein based solution and performed sonication and vortexing until becomes a well suspension. And then warmed in hot water bath (80 ~ 100℃) until becomes a well suspension.

No purity correction was applied. During the dosing, formulations were handled at room temperature, the remaining was discarded.

5.2 Analysis of Dose Formulations

Analysis of dose formulations such as stability, homogeneity and concentration verification was not performed in the test facility.

5.3 Justification of Administration Route

The oral gavage route was selected since it is the intended route of administration in humans.

5.4 Group Assignment and Dose Level

The study groups and dose level were assigned as below after consultation with the Sponsor.

Group	Treatment	Dose Level (mg/kg)	Dose Conc. (mg/mL)	No. of Animals	
				Toxicity	
				Males	Females
G1	Vehicle	0	0	3	3
G2	SLC-D011	500	50	3	3
G3	SLC-D011	2,000	200	3	3

5.5 Method of Administration

After overnight fasting (approximately 16 hours, food but not water should be withheld overnight), test article formulations were dosed using a plastic disposable feeding needle attached to a plastic disposable syringe. Food was withheld for a further 3~4 hours after dosing. Each dose was based on the most recent body weight of each animal and the dose volume was 10 mL/kg. The dosing day was designated as study Day 1.

6. OBSERVATIONS

6.1 Mortality

All animals were observed twice daily [once (am) on weekend and holiday] for mortality and moribundity during the study.

6.2 Clinical Observation

A clinical observation was performed for all animals at the time of dosing and approximately 1, 2 and 4 hours post-dose on dosing day, and once daily during 7-day observation period. Observations were included, but are not limited to, changes in the skin, fur, eyes and mucous membranes; respiratory, circulatory, autonomic and central nervous systems function; somatomotor activity and behavior patterns.

6.3 Body Weights

Individual body weights were measured for all animals on the day of animal receipt, randomization, prior to dosing start (Day 1) and study period (Days 4 and 7).

6.4 Gross Necropsy

Those animals survived on completion of the 7-day observation period were weighed body weight prior to necropsy and CO_2 inhalation anesthesia and exsanguinated from the abdominal aorta. In order to avoid autolytic change, a complete gross pathology examination of the carcass was performed as soon as possible after euthanasia of all animals. Necropsy was consisted of an external examination, including identification of all clinically recorded lesions, as well as a detailed internal examination.

7. STATISTICAL ANALYSES

The body weight data during the conduct of the study was subjected to calculation of group means and standard deviations. The statistical analysis was performed using GraphPad PRISM® Version 5.0 (GraphPad Software, USA).

8. RESULTS AND DISCUSSION

8.1 Mortality and Clinical Observation

All animals survived to the scheduled necropsy. There were no clinical abnormalities in all study animals.

8.2 Body Weights

There were no test article-related effects on body weight in all test article-treated groups compared to the vehicle control group.

8.3 Gross Necropsy

There were no toxicologically significant gross lesions in all test article-treated groups. Internal gross necropsy findings such as small thymus and enlarged adrenal glands were observed in only one male of 500 mg/kg group (animal No. M7). However, the toxicological signigicance of this change was not determined due to the lack of histologic examination.

9. CONCLUSION

Sprague-Dawley rats given single oral gavage administration of SLC-D011 at 500 and 2,000 mg/kg were well tolerated. Consequently, approximate lethal dose (ALD) of SLC-D011 is considered to be > 2,000 mg/kg of both sexes.

10. FIGURES

(GROUP SUMMARY)

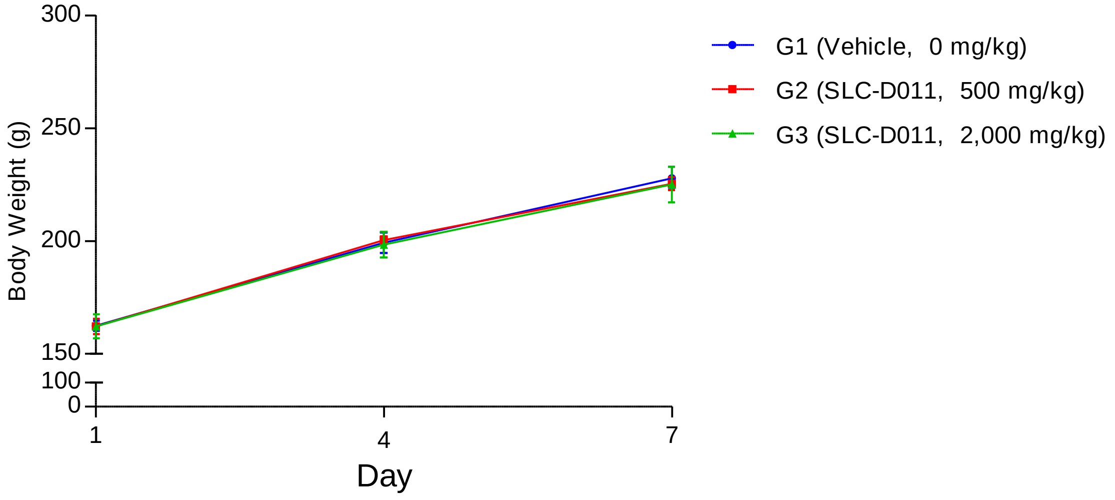

Figure 1. Body Weight in Male Rats (Group Summary)

(END)

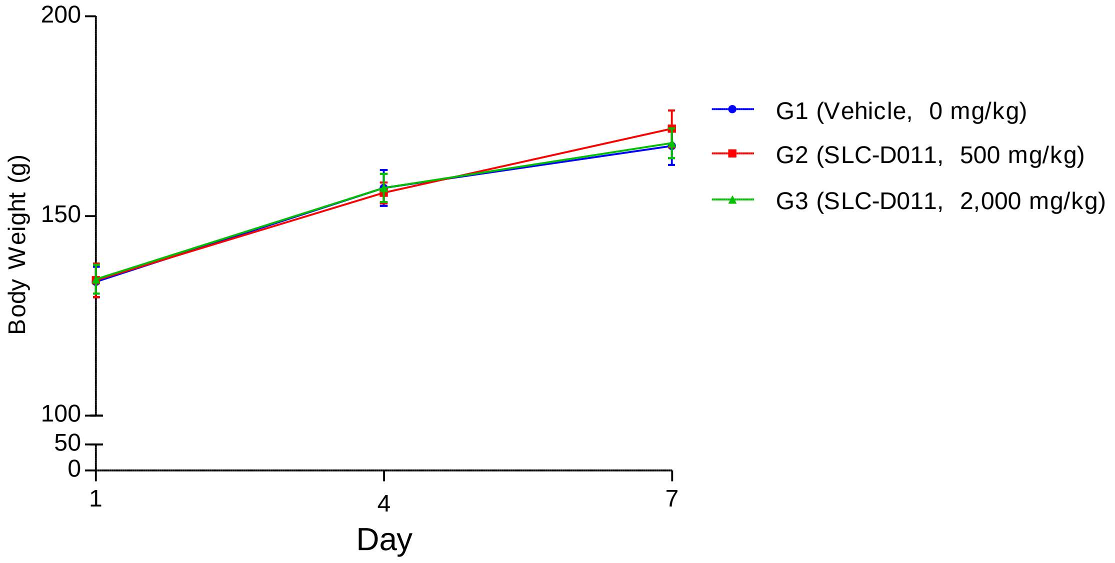

Figure 2. Body Weight in Female Rats (Group Summary)

(END)

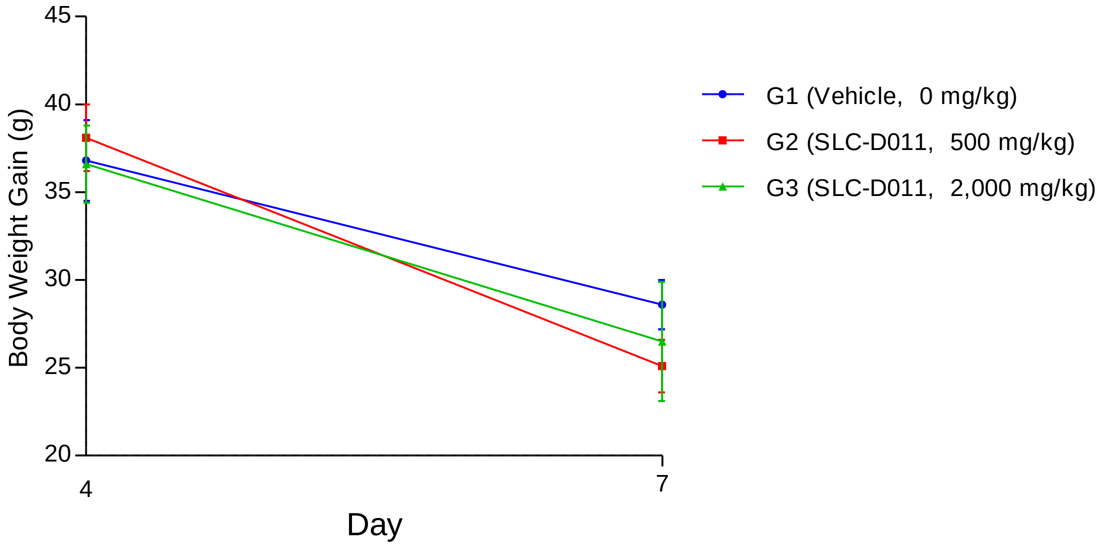

Figure 3. Body Weight Gain in Male Rats (Group Summary)

(END)

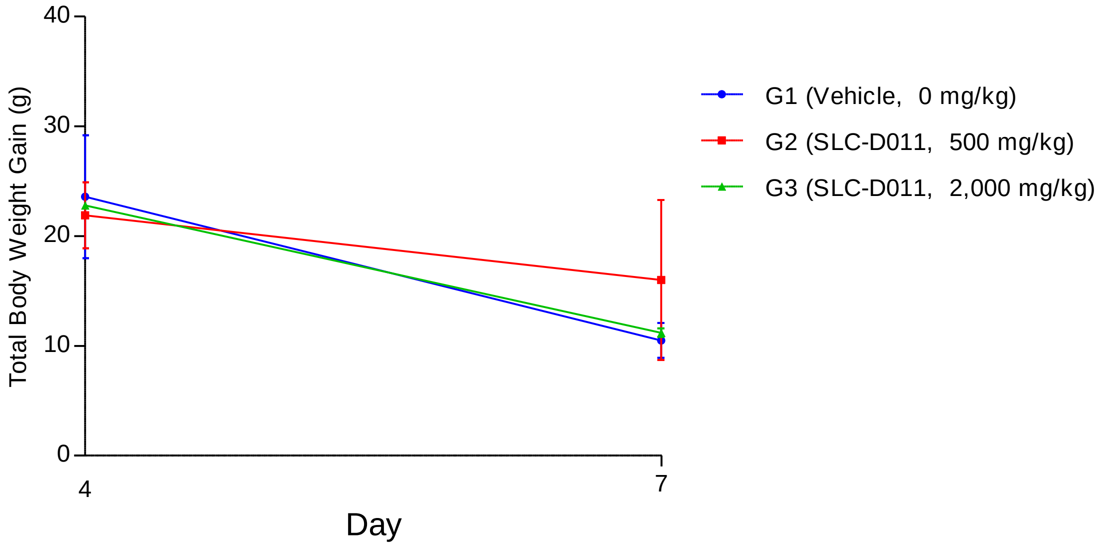

Figure 4. Body Weight Gain in Female Rats (Group Summary)

(END)

11. TABLES

(Group Summary)

TABLE 1. MORTALITY IN MALE RATS (SUMMARY)

Group/ Treatment	Dose Level (mg/kg)	No. of Animals	Mortality							Mortality (%)
			Number of Death							
			Day 1	Day 2	Day 3	Day 4	Day 5	Day 6	Day 7	
G1 Vehicle	0	3	0	0	0	0	0	0	0	0/3 (0)
G2 SLC-D011	500	3	0	0	0	0	0	0	0	0/3 (0)
G3 SLC-D011	2,000	3	0	0	0	0	0	0	0	0/3 (0)

(END)

TABLE 2. MORTALITY IN FEMALE RATS (GROUP SUMMARY)

Group/ Treatment	Dose Level (mg/kg)	No. of Animals	Mortality							Mortality (%)
			Number of Death							
			Day 1	Day 2	Day 3	Day 4	Day 5	Day 6	Day 7	
G1 Vehicle	0	3	0	0	0	0	0	0	0	0/3 (0)
G2 SLC-D011	500	3	0	0	0	0	0	0	0	0/3 (0)
G3 SLC-D011	2,000	3	0	0	0	0	0	0	0	0/3 (0)

(END)

TABLE 3. CLINICAL OBSERVATION IN RATS (GROUP SUMMARY)

Group/ Treatment	Dose Level (mg/kg)	No. of Animals	Clinical Observation	
			Male	Female
G1 Vehicle	0	3	Appears Normal	Appears Normal
G2 SLC-D011	500	3	Appears Normal	Appears Normal
G3 SLC-D011	2,000	3	Appears Normal	Appears Normal

(END)

TABLE 4. BODY WEIGHT AND BODY WEIGHT GAIN IN MALE RATS (GROUP SUMMARY)

Group/ Treatment	Dose Level (mg/kg)		Body Weight (g)			Body Weight Gain (g)		Total Gain
			Day 1	Day 4	Day 7	Day 4 - 1	Day 7 - 4	
G1 Vehicle	0	Mean	162.4	199.2	227.8	36.8	28.6	65.4
		SD	2.3	4.5	5.1	2.3	1.4	3.4
		N	3	3	3	3	3	3
G2 SLC-D011	500	Mean	162.2	200.3	225.4	38.1	25.1	63.2
		SD	3.4	1.7	2.9	1.9	1.5	0.6
		N	3	3	3	3	3	3
G3 SLC-D011	2,000	Mean	161.9	198.4	225.0	36.6	26.5	63.1
		SD	5.3	5.7	7.9	2.2	3.4	2.6
		N	3	3	3	3	3	3

N: Number of animals; SD: Standard deviation

(END)

TABLE 5. BODY WEIGHT AND BODY WEIGHT GAIN IN FEMALE RATS (GROUP SUMMARY)

Group/ Treatment	Dose Level (mg/kg)		Body Weight (g)			Body Weight Gain (g)		Total Gain
			Day 1	Day 4	Day 7	Day 4 - 1	Day 7 - 4	
G1 Vehicle	0	Mean	133.5	157.0	167.5	28.3	12.3	40.7
		SD	3.8	4.5	4.7	17.5	9.8	27.3
		N	3	3	3	24.9	9.3	34.2
G2 SLC-D011	500	Mean	133.9	155.8	171.8	23.5	23.5	47.0
		SD	4.2	2.6	4.6	23.8	9.0	32.8
		N	3	3	3	18.5	15.4	33.9
G3 SLC-D011	2,000	Mean	134.2	157.0	168.2	22.8	10.7	33.5
		SD	3.6	3.5	3.7	23.0	11.5	34.5
		N	3	3	3	22.7	11.3	34.0

N: Number of animals; SD: Standard deviation

(END)

TABLE 6. GROSS NECROPSY FINDING IN RATS (GROUP SUMMARY)

Group / Treatment<br>Dose Level (mg/kg)	Male			Female		
	G1<br>Vehicle	G2<br>SLC-D011	G3<br>SLC-D011	G1<br>Vehicle	G2<br>SLC-D011	G3<br>SLC-D011
	0	500	2,000	0	500	2,000
Number of animals						
Examined at terminal kill	3	3	3	3	3	3
External Gross Observation						
No gross findings	3	3	3	3	3	3
Internal Gross Observation						
Thymus						
small			1			
Adrenal glands						
enlarged			1			
No gross findings	3	3	2	3	3	3

(END)

12. APPENDICES

(Individual)

APPENDIX 1. CLINICAL OBSERVATION IN MALE RATS (INDIVIDUAL)

Group/ Treatment	Dose Level (mg/kg)	Animal No.	Clinical Observation						
			Day 1	Day 2	Day 3	Day 4	Day 5	Day 6	Day 7
G1 Vehicle	0	M1	N	N	N	N	N	N	N
		M2	N	N	N	N	N	N	N
		M3	N	N	N	N	N	N	N
G2 SLC-D011	500	M4	N	N	N	N	N	N	N
		M5	N	N	N	N	N	N	N
		M6	N	N	N	N	N	N	N
G3 SLC-D011	2,000	M7	N	N	N	N	N	N	N
		M8	N	N	N	N	N	N	N
		M9	N	N	N	N	N	N	N

N: Appears Normal

(END)

APPENDIX 2. CLINICAL OBSERVATION IN FEMALE RATS (INDIVIDUAL)

Group/ Treatment	Dose Level (mg/kg)	Animal No.	Clinical Observation						
			Day 1	Day 2	Day 3	Day 4	Day 5	Day 6	Day 7
G1 Vehicle	0	F10	N	N	N	N	N	N	N
		F11	N	N	N	N	N	N	N
		F12	N	N	N	N	N	N	N
G2 SLC-D011	500	F13	N	N	N	N	N	N	N
		F14	N	N	N	N	N	N	N
		F15	N	N	N	N	N	N	N
G3 SLC-D011	2,000	F16	N	N	N	N	N	N	N
		F17	N	N	N	N	N	N	N
		F18	N	N	N	N	N	N	N

N: Appears Normal

(END)

APPENDIX 3. BODY WEIGHT AND BODY WEIGHT GAIN IN MALE RATS (INDIVIDUAL)

Group/ Treatment	Dose Level (mg/kg)	Animal No.	Body weight (g)			Body weight Gain (g)		Total Gain (g)
			Day 1	Day 4	Day 7	Day 4 - 1	Day 7 - 4	
G1 Vehicle	0	M1	159.7	194.1	222.2	34.3	28.1	62.5
		M2	163.3	202.2	232.3	38.9	30.1	69.1
		M3	164.1	201.4	228.8	37.3	27.5	64.7
G2 SLC-D011	500	M4	158.5	198.8	222.1	40.3	23.3	63.6
		M5	162.9	200.0	226.3	37.2	26.2	63.4
		M6	165.3	202.1	227.8	36.9	25.6	62.5
G3 SLC-D011	2,000	M7	156.6	192.0	217.1	35.4	25.1	60.5
		M8	161.8	200.9	225.0	39.1	24.1	63.2
		M9	167.1	202.5	232.8	35.3	30.4	65.7

(END)

APPENDIX 4. BODY WEIGHT AND BODY WEIGHT GAIN IN FEMALE RATS (INDIVIDUAL)

Group/ Treatment	Dose Level (mg/kg)	Animal No.	Body weight (g)			Body weight Gain (g)		Total Gain (g)
			Day 1	Day 4	Day 7	Day 4 - 1	Day 7 - 4	
G1 Vehicle	0	F10	129.2	157.5	169.8	28.3	12.3	40.7
		F11	134.9	152.3	162.1	17.5	9.8	27.3
		F12	136.4	161.2	170.5	24.9	9.3	34.2
G2 SLC-D011	500	F13	129.5	152.9	176.4	23.5	23.5	47.0
		F14	134.3	158.1	167.1	23.8	9.0	32.8
		F15	137.9	156.4	171.9	18.5	15.4	33.9
G3 SLC-D011	2,000	F16	131.5	154.3	165.0	22.8	10.7	33.5
		F17	132.8	155.8	167.3	23.0	11.5	34.5
		F18	138.3	161.0	172.3	22.7	11.3	34.0

(END)

APPENDIX 5. GROSS NECROPSY FINDING IN MALE RATS (INDIVIDUAL)

Group/ Treatment	Dose Level (mg/kg)	Animal No.	Day of Necropsy	Gross Necropsy Finding
G1 Vehicle	0	M1	Day 8 Scheduled Euthanasia	External gross findings: No gross finding
				Internal gross findings: No gross finding
		M2	Day 8 Scheduled Euthanasia	External gross findings: No gross finding
				Internal gross findings: No gross finding
		M3	Day 8 Scheduled Euthanasia	External gross findings: No gross finding
				Internal gross findings: No gross finding
G2 SLC-D011	500	M4	Day 8 Scheduled Euthanasia	External gross findings: No gross finding
				Internal gross findings: No gross finding
		M5	Day 8 Scheduled Euthanasia	External gross findings: No gross finding
				Internal gross findings: No gross finding
		M6	Day 8 Scheduled Euthanasia	External gross findings: No gross finding
				Internal gross findings: No gross finding
G3 SLC-D011	2,000	M7	Day 8 Scheduled Euthanasia	External gross findings: No gross finding
				Internal gross findings: Thymus, small (slight); Adrenal glands, enlarged (slight)
		M8	Day 8 Scheduled Euthanasia	External gross findings: No gross finding
				Internal gross findings: No gross finding
		M9	Day 8 Scheduled Euthanasia	External gross findings: No gross finding
				Internal gross findings: No gross finding

(END)

APPENDIX 6. GROSS NECROPSY FINDINGS IN FEMALE RATS (INDIVIDUAL)

Group/ Treatment	Dose Level (mg/kg)	Animal No.	Day of Necropsy	Gross Necropsy Finding
G1 Vehicle	0	F10	Day 8 Scheduled Euthanasia	External gross findings: No gross finding
				Internal gross findings: No gross finding
		F11	Day 8 Scheduled Euthanasia	External gross findings: No gross finding
				Internal gross findings: No gross finding
		F12	Day 8 Scheduled Euthanasia	External gross findings: No gross finding
				Internal gross findings: No gross finding
G2 SLC-D011	500	F13	Day 8 Scheduled Euthanasia	External gross findings: No gross finding
				Internal gross findings: No gross finding
		F14	Day 8 Scheduled Euthanasia	External gross findings: No gross finding
				Internal gross findings: No gross finding
		F15	Day 8 Scheduled Euthanasia	External gross findings: No gross finding
				Internal gross findings: No gross finding
G3 SLC-D011	2,000	F16	Day 8 Scheduled Euthanasia	External gross findings: No gross finding
				Internal gross findings: No gross finding
		F17	Day 8 Scheduled Euthanasia	External gross findings: No gross finding
				Internal gross findings: No gross finding
		F18	Day 8 Scheduled Euthanasia	External gross findings: No gross finding
				Internal gross findings: No gross finding

(END)

APPENDIX 7. PROTOCOL

Study No. : QT18122

PROTOCOL

SLC-D011:
A SINGLE ORAL GAVAGE DOSE TOXICITY STUDY IN SD RATS

STUDY NUMBER: QT18122

DRUG EVALUATION LABORATORY
QUBEST BIO CO., LTD.

www.QuBEST.co.kr TEL 031-706-2995 FAX 031-706-2996 E-mail info@qubest.co.kr
The BEST Partner for Your Successful Drug Development

(CONTINUED)

APPENDIX 7. PROTOCOL

Study No. : QT18122

PROTOCOL APPROVAL

STUDY TITLE: SLC-D011: A SINGLE ORAL GAVAGE DOSE TOXICITY STUDY IN SD RATS

STUDY NUMBER: QT18122

STUDY DIRECTOR JINYOUNG KIM, MS 24 / Aug / 2018

Drug Evaluation Laboratory/QuBEST BIO Co., Ltd.
Lab. Animal Center, School of Dentistry, Seoul National University
1 Gwanak-ro, Gwanak-gu, Seoul, 08826, Korea
TEL: 02-3296-2995, FAX: 02-949-2996
E-mail: amajones@qubest.co.kr

MANAGEMENT SOOHYEON KIM, MS 24 / AUG / 2018

QuBEST BIO Co., Ltd.
#1304, U-TOWER, 120 Heungdeokjungang-ro, Giheung-gu, Yongin-si
Gyeonggi-do, 16950, Korea
TEL: 031-706-2995, FAX: 031-706-2996
E-mail: shkim@qubest.co.kr

SPONSOR MINJU KIM, MS 김 민주 24 / Aug / 2018

PRG S&T Co., Ltd.
Room 306, Busandaehak-ro 63-beongil 2, Geumjeong-gu, Busan
Republic of Korea
TEL: 051-510-7562, FAX: 051-510-7563
E-mail: rlaals@naver.com

(CONTINUED)

APPENDIX 7. PROTOCOL

Study No. : QT18122

TABLE OF CONTENTS

PROTOCOL APPROVAL 2
TABLE OF CONTENTS 3
1. STUDY SUMMARY 5
1.1 STUDY TITLE 5
1.2 STUDY OBJECTIVE 5
1.3 REGULATORY TEST GUIDELINE 5
1.4 PROPOSED STUDY SCHEDULE 5
2. TEST ARTICLE AND VEHICLE INFORMATION 5
2.1 TEST ARTICLE 5
2.2 VEHICLE 5
3. TEST SYSTEM 6
3.1 ANIMALS AND RECEIPT 6
3.2 HUSBANDRY AND ENVIRONMENTAL CONDITIONS 6
3.3 FEED AND DRINKING WATER 7
4. STUDY METHOD 7
4.1 ANIMAL RECEIVING AND ACCLIMATION 7
4.2 RANDOMIZATION 7
4.3 ADMINISTRATION ROUTE AND JUSTIFICATION 7
4.4 STUDY GROUPS AND DOSE LEVELS 8
4.5 ADMINISTRATION 8
5. DOSE FORMULATION PREPARATION AND ANALYSIS 8
5.1 METHOD AND FREQUENCY OF DOSE FORMULATION PREPARATION 8
5.2 CHEMICAL ANALYSIS OF DOSE FORMULATION 8
6. OBSERVATIONS 9
6.1 MORTALITY AND CLINICAL OBSERVATIONS 9
6.2 BODY WEIGHT 9

3

(CONTINUED)

APPENDIX 7. PROTOCOL

Study No. : QT18122

6.3 GROSS NECROPSY 9

7. STATISTICAL ANALYSIS 9

8. FINAL REPORT AND DATA RETENTION 9

9. PROTOCOL AMENDMENT 10

10. IACUC APPROVAL 10

APPENDIX 7. PROTOCOL

1. STUDY SUMMARY

1.1 STUDY TITLE

SLC-D011: A SINGLE ORAL GAVAGE DOSE TOXICITY STUDY IN SD RATS

1.2 STUDY OBJECTIVE

The objective of this study is to investigate the potential acute toxicity or approximate lethal dose of SLC-D011 following a single oral gavage administration to the SD rats.

1.3 REGULATORY TEST GUIDELINE

No specific regulatory guidelines. This study is conducted according to study-specific protocol after consultation with the Sponsor.

1.4 PROPOSED STUDY SCHEDULE

Animal receipt:	Aug 20, 2018
Administration:	Aug 26, 2018
Necropsy:	Sep 2, 2018
Draft report:	Oct 20, 2018

2. TEST ARTICLE AND VEHICLE INFORMATION

2.1 TEST ARTICLE

Identity:	SLC-D011
Batch/Lot number:	A05064-013S2
Appearance:	White powder
Purity (Activity assay):	99.2% (by HPLC)
Storage conditions:	Keep container tightly closed to avoid light in deep freezer (-70 ℃)
Handling precautions:	Routine protection procedures with gloves, goggles etc. and avoid contact with skin, eye and inhalation of vapor/mist

The test article is manufactured according to non-GLP/non-GMP regulations. The appropriate details on test article will be included in the final report. The test article remaining at study completion will be returned to the Sponsor.

2.2 VEHICLE

Identity:	Oil based solution (Monoolein : Tricaprylin = 2 : 1)
Supplier:	Drug Evaluation Laboratory, QuBEST BIO Co., Ltd.
Appearance:	Clear colorless liquid

(CONTINUED)

APPENDIX 7. PROTOCOL

Storage conditions:	Room temperature

1) **VEHICLE COMPONENT** 1

Identity:	Monoolein
Batch/Lot number:	RRNFJAT
Supplier:	TCI (Tokyo Chemical Industry Co., LTD.)
Appearance:	Clear colorless liquid
Storage conditions:	Room temperature

2) **VEHICLE COMPONENT** 2

Identity:	Tricaprylin
Batch/Lot number:	KEWXE-AL
Supplier:	TCI (Tokyo Chemical Industry Co., LTD.)
Appearance:	Clear colorless liquid
Storage conditions:	Room temperature

3. TEST SYSTEM

3.1 ANIMALS AND RECEIPT

Species:	Rat (*Rattus norvegicus*)
Strain:	Sprague-Dawley Rat (Specific pathogen free)
Source:	SAMTAKO (Osan, Korea)
Age at receipt:	Approximately 5 weeks
Age and weight range at treatment start:	Approximately 6 weeks, mean body weight (g) ± 10%
Number of animals:	Twenty two rats (11 males and 11 females) will be ordered and eighteen rats (9 males and 9 females) will be assigned to the study.
Justification for species:	The Sprague-Dawley rats are selected because it is a standard species for use in toxicity study. The number of animals used in the study will be considered by the Study Director to be the minimum number to obtain the meaningful scientific results.

3.2 HUSBANDRY AND ENVIRONMENTAL CONDITIONS

Study animals will be single housed in wire grid bottom cage [220 W x 300 D x 180 H (mm)] with polycarbonate water bottles. Animal room and each cage/bottle will be cleaned at regular intervals per SOP. Each cage will be clearly labeled with a color-coded cage card indicating study number, group, animal numbers and sex and animals will be uniquely identified using tail marking method (black).

(CONTINUED)

APPENDIX 7. PROTOCOL

The targeted conditions for animal room environment and photoperiod will be as follows:

HVAC conditions:	100% HEPA-filtered air, at least 10 air changes/hr
Temperature and humidity:	22 ± 3℃, 50 ± 20% (relative humidity)
Light cycle and intensity:	12 hours light and 12 hours dark (on: 08:00~20:00, except during designated procedures in the protocol), intensity 150~300 Lux

3.3 FEED AND DRINKING WATER

All animals will have free access to standard irradiated pelleted commercial laboratory diet (Purina Rodent Chow 38057, Korea) and water except during designated procedures in the protocol.

Each batch of diet is delivered with an accompanying certificate of analysis detailing nutritional composition and levels of specified contaminants. Maximum allowable levels of contaminants in diet are controlled and routinely analyzed by the manufacturers. Periodic analysis of municipal tap water for microbiological purity and levels of contaminants is subcontracted to management authorized analytical laboratories and the results are reviewed by the testing facility per "Quality Standard for Drinking Water and Water Quality Criteria". The analytical results of the diet and water are retained in the archives of testing facility.

4. STUDY METHOD

4.1 ANIMAL RECEIVING AND ACCLIMATION

Following arrival, each animal will be given a general physical examination by a qualified study personnel and/or study director to assess health status. And then each animal will be weighed, temporarily identified using tail marking method (red) and acclimated to the study room at least 5 days. During the acclimation, animals will be weighed, observed once daily and cared as same as study period.

4.2 RANDOMIZATION

On the last day of acclimation period, all animals will be weighed, evaluated for general health and suitability of testing and those considered suitable for the study will be released to the study. And then animals will be randomly assigned to study groups based on the body weight. Prior to the initiation of dosing and/or immediately after dosing, any assigned animals considered unsuitable for use and/or accidental events will be replaced by spare animals with appropriate documentation.

4.3 ADMINISTRATION ROUTE AND JUSTIFICATION

The route of administration will be selected as oral gavage route by the Sponsor's request.

(CONTINUED)

APPENDIX 7. PROTOCOL

Study No. : QT18122

4.4 STUDY GROUPS AND DOSE LEVELS

The study groups are assigned as below after consultation with the Sponsor.

Group	Treatment	Dose Level (mg/kg)	Dose Conc.† (mg/mL)	No. of Animals	
				Toxicity	
				Males	Females
G1	SLC-D011	Vehicle	0	3	3
G2		500	50	3	3
G3		2,000	200	3	3

† Dosing volume: 10 mL/kg (Dosing volume will be changed considering the properties of the preparation)

4.5 ADMINISTRATION

After overnight fasting (approximately 16 hours, food but not water should be withheld overnight), the test article formulation will be dosed once using a plastic disposable feeding needle attached to a plastic disposable syringe. Food is withheld for a further 3~4 hours after dosing. Individual doses are calculated based on the most recent body weight to provide the proper dose.

5. DOSE FORMULATION PREPARATION AND ANALYSIS

5.1 METHOD AND FREQUENCY OF DOSE FORMULATION PREPARATION

The dose formulations will be freshly prepared in clean bench prior to dosing according to Sponsor's provided mixing procedure.

Monoolein based solution (Monoolein : Tricaprylin = 2 : 1) for PO

Pre-heat the monoolein based solution to a temperature above 80℃. Add pre-heating the monoolein based solution and perform sonication and vortexing until becomes a well suspension. And then warmed in hot water bath (80 ~ 100℃) until becomes a well suspension.

No purity correction will be applied. During the dosing, formulations will be handled at room temperature, the remaining will be discarded.

5.2 CHEMICAL ANALYSIS OF DOSE FORMULATION

Analysis of dose formulations such as stability, homogeneity and concentration verification will not be performed in the testing facility.

(CONTINUED)

APPENDIX 7. PROTOCOL

6. OBSERVATIONS

6.1 MORTALITY AND CLINICAL OBSERVATIONS

All animals will be observed twice daily [once (am) on weekend and holiday] for mortality and moribundity during the study.

A clinical observation will be performed for all animals at the time of dosing and approximately 1, 2 and 4 hours post-dose on dosing day, and once daily during 7-day observation period. Observations is included, but are not limited to, changes in the skin, fur, eyes and mucous membranes; respiratory, circulatory, autonomic and central nervous systems function; somatomotor activity and behavior patterns.

6.2 BODY WEIGHT

Individual body weights will be measured for all animals on the day of animal receipt, randomization, prior to dosing start (Day 1) and study Days (Single Tox phase: on Days 4 and 7).

6.3 GROSS NECROPSY

The animals found dead during the study will not be subjected to necropsy. Those animals survived on completion of the 7-day observation period will be subjected to necropsy on Day 8. After ether inhalation anesthesia, major blood vessels are severed to exsanguinate the animal. And then a complete gross pathology examination of the carcass is performed as soon as possible after euthanasia. Necropsy consisted of an external examination, including identification of all clinically recorded lesions, as well as a detailed internal examination.

7. STATISTICAL ANALYSIS

The body weight data during the conduct of the study will be subjected to calculation of group means and standard deviations. The statistical analysis will be performed using GraphPad PRISM® Version 5.0 (GraphPad Software, USA).

8. FINAL REPORT AND DATA RETENTION

All study related records and raw data will be filed as Study File and the study director will prepare draft report based on raw data and send to the Sponsor for review and comments before the report finalization. The original final report (one unbound) will be supplied to the Sponsor. The original signed protocol, amendments and all raw data relating to this study will be stored in the testing facility for a period of 6 months.

(CONTINUED)

APPENDIX 7. PROTOCOL

9. PROTOCOL AMENDMENT

As necessary during the study, the study director will take appropriate action, in consultation with the management and the Sponsor (if possible). Untoward changes or events occurring during the study will be communicated to the Sponsor by telephone, telefax or e-mail. Agreed upon changes in study conduct will be filed and/or documented in the Study File.

10. IACUC APPROVAL

The protocol and procedures involving the care and use of animals in this study will be reviewed and approved by IACUC of QuBEST BIO prior to conduct (Approval No.: QBSIACUC-A18122). During the study, the care and use of animals will be conducted in accordance with all applicable guidelines of Animal Welfare Act (revised January 20, 2015).

(END)

13. ANNEXES

ANNEX 1. CERTIFICATE OF ANALYSIS (COA)

康龙化成
PHARMARON

Report of Analysis

Project ID	PH-QBC-SLC-D011	Version	V01
Batch Number	A05064-013S2	Client ID	SLC-D011
Manufacture Date	8Aug2018	Batch Size	23.5 g
Test Date	13Aug2018	Retest Date	12Aug2019
Storage Condition	Stored in well closed container at ambient temperature		
Note	All tests were performed in non-GMP analytical laboratories.		

Tested Items	Method	Specification	Result
Appearance	Visual	Report	White powder
^{1}H-NMR	NMR	Consistent with structure	Consistent with structure
LCMS	LCMS	Consistent with exact mass	Consistent with exact mass
Purity (area%)	HPLC	≥ 97.5%	99.2%
Chiral Purity (area%)	Chiral HPLC	≥99%	100%

Conclusion: All results meet the acceptance criteria in the specification

Revision Log:

Author	Description	Effective Date
Quanyin Guan	Original Issue	17Aug2018

Prepared by/Date : Quanyin Guan 17Aug2018

Reviewed by/Date : Xiaoting Guo 17Aug2018

Address: Pharmaron (Ningbo), Inc. No.800 Bin-Hai 4th Road, Hangzhou Bay New Zone, Ningbo, China315336. Tel: 0574-23450698

(END)

ANNEX 2. ANIMAL HEALTH MONITORING REPORT

IDEXX BioResearch

FINAL REPORT OF LABORATORY EXAMINATION

4011 Discovery Drive, Columbia, MO 65201
1-800-669-0825 1-573-499-5700
idexxbioresearch@idexx.com www.idexxbioresearch.com

IDEXX BioResearch Case # 1555-2018

Received: 7/16/2018
Completed: 7/23/2018

Submitted By

Jaeyoung Choi
Samtako Bio Korea
105, Seorang-ro
OSan-Si Gyeonggi-do 18100
South Korea

Phone: +82-31-372-3902
Fax: +82-31-372-3654
Email: bio@samtako.com

Specimen Description

Species: rat

Number of Specimens/Animals: 10

Client ID	Room #	Strain	Sex	Age
No. 1	202	Sprague Dawley	M	8w
No. 2	202	Sprague Dawley	M	8w
No. 3	202	Sprague Dawley	M	8w
No. 4	202	Sprague Dawley	M	8w
No. 5	202	Sprague Dawley	M	8w
No. 6	202	Sprague Dawley	M	11w
No. 7	202	Sprague Dawley	M	11w
No. 8	202	Sprague Dawley	M	11w
No. 9	202	Sprague Dawley	M	11w
No. 10	202	Sprague Dawley	M	11w

Services/Tests Performed: Comprehensive Necropsy; Micro Panel 4; Helicobacter

Serologic evaluation for antibodies to: *Clostridium piliforme, Mycoplasma pulmonis,* CAR bacillus, H1, Hantaan, KRV, LCMV, MAV1, MAV2, PVM, RCV/SDAV, REO3, RMV, RPV, RTV, Sendai

PCR evaluation for: *Helicobacter bilis, Helicobacter ganmani, Helicobacter hepaticus, Helicobacter mastomyrinus, Helicobacter rodentium, Helicobacter* spp., *Helicobacter typhlonius, Mycoplasma pulmonis, Streptobacillus moniliformis*

Parasitologic evaluation for: Fur mites, mesostigmatid mites, lice, *Spironucleus muris, Giardia muris,* large intestinal flagellates, and amoeba, pinworms and tapeworms

Microbiologic evaluation for: *Bordetella bronchiseptica, Corynebacterium kutscheri, Klebsiella pneumoniae, Pasteurella multocida, Pasteurella pneumotropica, Pasteurella pneumotropica* biotype Heyl,*Pasteurella pneumotropica* biotype Jawetz, *Pseudomonas aeruginosa, Salmonella enterica, Streptococcus pneumoniae*

Summary: All test results were negative.

Cindy Besch-Williford
Cynthia Besch-Williford, DVM, PhD

If you have questions, please call our toll free number at 1-800-669-0825 or e-mail us at idexx-radil@idexx.com.

Page 1 of 3 IDEXX BioResearch Case # 1555-2018

(CONTINUED)

ANNEX 2. ANIMAL HEALTH MONITORING REPORT

NECROPSY

No gross lesions were detected in any of the body systems and organs examined.

SEROLOGY SUMMARY

	No. 1	No. 2	No. 3	No. 4	No. 5	No. 6	No. 7	No. 8	No. 9	No. 10
CAR bacillus	-	-	-	-	-	-	-	-	-	-
Hantaan	-	-	-	-	-	-	-	-	-	-
LCMV	-	-	-	-	-	-	-	-	-	-
Mycoplasma pulmonis	-	-	-	-	-	-	-	-	-	-
MAV1	-	-	-	-	-	-	-	-	-	-
MAV2	-	-	-	-	-	-	-	-	-	-
RPV	-	-	-	-	-	-	-	-	-	-
RMV	-	-	-	-	-	-	-	-	-	-
KRV	-	-	-	-	-	-	-	-	-	-
H1	-	-	-	-	-	-	-	-	-	-
PVM	-	-	-	-	-	-	-	-	-	-
RCV/SDAV	-	-	-	-	-	-	-	-	-	-
REO3	-	-	-	-	-	-	-	-	-	-
RTV	-	-	-	-	-	-	-	-	-	-
Sendai	-	-	-	-	-	-	-	-	-	-
Clostridium piliforme	-	-	-	-	-	-	-	-	-	-
Rat IgG	N	N	N	N	N	N	N	N	N	N

Legend: + = positive - = negative blank = test not performed EQ = equivocal HE = hemolysis precluded testing I = insufficient W = weak positive WB = Western Blot confirmatory analysis pending NS = non-specific reactivity N = normal IgG L = less than normal IgG

PCR EVALUATION

oral swab	No. 1	No. 2	No. 3	No. 4	No. 5	No. 6	No. 7	No. 8	No. 9	No. 10
Mycoplasma pulmonis	-	-	-	-	-	-	-	-	-	-
Streptobacillus moniliformis	-	-	-	-	-	-	-	-	-	-

feces	No. 1	No. 2	No. 3	No. 4	No. 5	No. 6	No. 7	No. 8	No. 9	No. 10
Helicobacter spp.	-	-	-	-	-	-	-	-	-	-
Helicobacter bilis	-	-	-	-	-	-	-	-	-	-
Helicobacter ganmani	-	-	-	-	-	-	-	-	-	-
Helicobacter hepaticus	-	-	-	-	-	-	-	-	-	-
Helicobacter mastomyrinus	-	-	-	-	-	-	-	-	-	-
Helicobacter rodentium	-	-	-	-	-	-	-	-	-	-
Helicobacter typhlonius	-	-	-	-	-	-	-	-	-	-

Legend: + = positive - = negative id:id = pooled sample range id+id+id = non-range pooled sample NT or blank = no test performed wps = weak positive

Page 2 of 3 IDEXX BioResearch Case # 1555-2018

(CONTINUED)

ANNEX 2. ANIMAL HEALTH MONITORING REPORT

Comments: All samples are first tested by the Helicobacter spp. PCR assay which is a generic Helicobacter PCR assay that detects all bacteria in the Helicobacter genus. Samples testing negative on this assay are reported negative for Helicobacter spp. and all species-specific helicobacters. Samples testing positive on the Helicobacter spp. assay are retested with the Helicobacter spp. assay and tested with the species-specific helicobacter PCR assays.

PARASITOLOGY

	No. 1	No. 2	No. 3	No. 4	No. 5	No. 6	No. 7	No. 8	No. 9	No. 10
parasites	-	-	-	-	-	-	-	-	-	-

MICROBIOLOGY

cecum	No. 1	No. 2	No. 3	No. 4	No. 5	No. 6	No. 7	No. 8	No. 9	No. 10
Klebsiella pneumoniae	-	-	-	-	-	-	-	-	-	-
Pseudomonas aeruginosa	-	-	-	-	-	-	-	-	-	-
Salmonella enterica	-	-	-	-	-	-	-	-	-	-

nasopharynx	No. 1	No. 2	No. 3	No. 4	No. 5	No. 6	No. 7	No. 8	No. 9	No. 10
Bordetella bronchiseptica	-	-	-	-	-	-	-	-	-	-
Corynebacterium kutscheri	-	-	-	-	-	-	-	-	-	-
Pasteurella multocida	-	-	-	-	-	-	-	-	-	-
Pasteurella pneumotropica	-	-	-	-	-	-	-	-	-	-
Pasteurella pneumotropica biotype Heyl	-	-	-	-	-	-	-	-	-	-
Pasteurella pneumotropica biotype Jawetz	-	-	-	-	-	-	-	-	-	-
Staphylococcus pneumoniae	-	-	-	-	-	-	-	-	-	-

Legend: + = agent recovered - = agent not recovered blank = test not performed n = no growth p = Proteus overgrowth, which may interfere with the identification of other bacteria

Page 3 of 3 IDEXX BioResearch Case # 1555-2018

(END)

ANNEX 3. CERTIFICATE OF BEDDING GAMMA IRRADIATION

GREENPIA TECHNOLOGY

ISO
ISO9001/ISO13485/ISO11137
US FDA
Registered Contract Sterilizer
일본 후생노동성(**MHLW**)
Accreditation of Contract Sterilizer
ILAC-KOLAS 국제공인시험기관 인정

GREENPIA TECHNOLOGY

Certificate
Of Gamma Irradiation

We hereby certify that the goods specified as follow have been duly irradiated by gamma-ray.
(하기에 명시된 제품은 정히 조사되었음을 확인합니다.)

Certificate No. : GR-2018-08142

General Information

Customer : 계룡과학 주식회사

Product	Product Lot No.
깔짚(톱밥)	

Quantity : 60 C/T

Irradiation Result

Irradiation date : 2018-07-07
Date Issued : 2018-07-09
Specified Dose : 25 kGy

Approved by :
Quality Assurance Department

GREENPIA TECHNOLOGY

· A certificate with the exception of this certificate shall not be recognized and this certificate should not be reproduced except in full without the written approval of the Q.A dept.
(본 확인서 외에 어떤 확인서도 인정되지 않으며, 품질보증부의 서면 승인없이 일부라도 복사할 수 없음)
· Lot No. and quantity are based on those created with customer request form and Packing List.
(롯트번호와 수량은 의뢰자의 의뢰서와 Packing List에 명시한 것에 근거함)

GREENPIA TECHNOLOGY | 132 Majang-ro, Neungseo-myeon, Yeoju-si, Gyeonggi-do, 12641, Korea
TEL. 82-31-882-5366 FAX. 82-31-883-5403

(QI-120101-01) (Rev : 10/01.05.17)

(END)

ANNEX 4. BEDDING ANALYSIS REPORT

KRIAT 시험성적서

- 주소 : 대전광역시 유성구 용산동 테크노2로 125-7 ㈜한국분석기술연구소
- Tel : 042-823-7241 ▪ Fax : 042-823-6263

- 성적서 번호 : T170425
- 페이지 : (1) / (총 2)

1. 의뢰자
   - 기관명 : (주)샘타코 BIOKOREA
   - 의뢰자 : 최재영
   - 전화/팩스 : 031-372-3902 / -- ▪ 의뢰일자 : 2017.08.04
   - 주소 : 경기도 오산시 서랑로 105(서랑동 77-1)

2. 시험성적의 용도 : 참고용

3. 시료명(수량) : Aspen Shaving실험동물용 깔짚(Lot No. Sam-GB-L/M/S-201701-001~201712-300) 포함 총 1건

4. 시험기간 : 2017.08.04 ~ 2017.08.10

5. 분석장비 및 시험방법 :

6. 시험결과 : 별첨

확인	작성자		승인자	
	직위	연구원	기술책임자 직위	분석팀장
	성명	이지나	성명	서민정

2017년 8월 10일

(주) 한국분석 기술연구소 대표이사

- 본 성적서는 고객이 제공한 시료를 시험한 결과로써 전체 제품에 대한 품질을 보증하지 않음.
- 본 성적서는 상기 용도 이외 사용을 할 수 없으며 광고, 전단, 홍보 및 법적 쟁송의 수단으로 사용할 수 없음.
- 본 결과는 의뢰자가 제공한 시료로부터 얻은 것으로 유사 대상 시료에 적용할 수 없음.

(CONTINUED)

ANNEX 4. BEDDING ANALYSIS REPORT

시험결과

성적서 번호<br>Test No.	T170425	(2) 페이지 중 (2) 페이지<br>Page of Page

시료	원소	분석결과	단위	정량한계	시험방법
Aspen Shaving실험동물용 깔짚(Lot No. Sam-GB-L/M/S-201701-001~201712-300)	Cd	불검출	mg/kg	0.2	ICP-MS
	Pb	불검출	mg/kg	0.2	ICP-MS
	As	불검출	mg/kg	0.2	ICP-MS
	Hg	불검출	mg/kg	0.2	ICP-MS
	수분	11.9	%	*	수분측정기

이하여백

비고

(END)

ANNEX 5. CERTIFICATE OF FEED GAMMA IRRADIATION

Certificate
of gamma irradiation

Technology for better life! SOYA SOYAGREENTEC Co., Ltd.

**Certificate No.**	:	S 180713		0833	
**Customer**	:	샘타코바이오코리아			
**Irradiation Batch No.**	:	180711-29			
**Irradiation Container**	:	24	~	28	
**Dosimetry Results**	:	-	~	25.55	kGy
**Irradiated Date**	:	12-Jul-18	~	13-Jul-18	

Item Specification	QTY(Kg)	Lot No.	Specified Dose(kGy)	
			Dmin	Dmax
퓨리나 마우스 사료	400	–	-	26
Total	400			

*** Affirmation**

Date : 13-Jul-18

Approved : Kyou Young, Lee (Q.M.R / Director)

We hereby certify that the above specified goods have been duly irradiated by gamma-ray.

(상기에 명시된 제품은 정히 조사되었음을 확인합니다.)

EN / ISO
ISO 9001 & ISO 13485
ISO 11137-1 Certified

Registered : Contract sterilizer

厚生勞働省
Ministry of Health, Labour and Welfare

Technology for better life!
SOYAGREENTEC

Address : 34-26, Jeyakgongdan 2-gil, Hyangnam-eup, Hwaseong-si, Gyeonggi-do, 18622, Korea
TEL : +82-31-353-6999
FAX : +82-31-353-6979

(Form : PQ-101-10) (Rev : 15.12.02.)

(END)

ANNEX 6. FEED ANALYSIS RESULT

Cargill Agri Purina Inc.
Analysis Service of Central Laboratory

45, Pyeongtaek-Hang an-ro, Poseung-Eup
Pyengtaek-Si Gyonggi-do, Korea
Tel (031)8046-9577 Fax (031)684-5315

ANALYSIS RESULT

LAB NO. 330151 ENTERED Jan. 15, 2018 REPORTED Feb. 18, 2018

38057	LAB RODENT CHOW
PRODUCTION	01/09/18 KSN Plant
LOT NO.	2018-01-09

ASSAY	ANALYSIS	UNIT
NUTRIENTS		
MOISTURE(OVEN)	8.27	%
PROTEIN(PROTEIN ANALYZER)	21.71	%
FAT(ACID HYDROLYSIS)	5.45	%
FIBER(ANKOM)	6.03	%
ASH(FURNACE)	6.59	%
CALCIUM(ICP)	1.37	%
PHOSPHORUS(ICP)	0.67	%
HEAVY METALS		
As(ICP)	Not Detected	ppm
Cd(ICP)	0.06	ppm
Hg(MERCURY ANALYZER)	0.007	ppb
Pb(ICP)	Not Detected	ppm
Cr(ICP)	1.03	ppm
F(ION METER)	16.25	ppm
AFLATOXIN(HPLC)		
B1, B2, G1, G2	3.76	ppb
OCHRATOXIN(HPLC)	Not Detected	ppb
CHLORINATED HYDROCARBON		
DDT(GC)	Not Detected	ppm
ORGANOPHOSPHATES		
MALATHION(GC)	Not Detected	ppm
Microbial Tests		
Salmonella	Not Detected	cfu/g
Total Bacteria	1,000	cfu/g
E. Coli	Not Detected	cfu/g
PHYSICAL PROPERTIES		
PELLET SIZE(DxL)	18.73*14.37	mm
PELLET COLOR	Brown	-
HARDNESS	15.2	kg/cm2

THE LETTER CODE LOCATED BELOW EACH ASSAY IS A METHOD REFERENCE CODE. FOR ADDITIONAL INFORMATION CONTACT Jaeyeon Koo, CENTRAL LABORATORY MANAGER. (031-8046-9577)

PREPARED BY Yoojin Hong CONFIRMED BY Jae Yeon, Koo

The Term "Less Than" is used to signify the lower limit of quantitation of the procedure under the conditions employed. The use of the term "Less Than" does not imply that traces of analyte were present. Samples submitted to Central Laboratory Services for routine analysis will be retained for a minimum of thirty(30) days after the report of analysis is issued. Extended storage requirements must be brought to the attention of Central Laboratory services prior to or at the time of sample submission.

(END)

ANNEX 7. TAP WATER ANALYSIS RESULT

수 질 검 사 성 적 서

접 수 번 호 : W201707-02-0048
발 신 일 자 : 2017-07-20
발 신 : 워터스생활환경연구소
받 음 : 서울특별시 관악구 관악로 1 (신림동, 서울대학교)
책 임 자 : 소 재 호
제 목 : **수질검사성적서 교부**

「먹는물 수질기준 및 검사 등에 관한 규칙」 제3조2항에 따라 아래와 같이 먹는물 수질검사성적서를 통보합니다.

1. 시료내용

시 료 명	수도수(58항목)	관 련 법 령		입 회 자	신성묵 / 워터스생활환경연구소 /
상 호 명	치의과대학원	검 사 목 적	자체관리	채 수 일 자	2017-07-06
시 설 명	동물실험실수도수	신 고 번 호		접 수 일 자	2017-07-06
채 수 장 소	서울특별시 관악구 관악로 1 B106호				

2. 수질검사 결과

가. 검사결과 종합

판정	적합
수질기준 초과항목	

나. 항목별 검사결과

검사항목	기 준	결 과	검사항목	기 준	결 과
일반세균	100 CFU/mL 이하	0	크실렌	0.5 mg/L이하	불검출
총대장균군	불검출/100 mL	불검출	1,1-디클로로에틸렌	0.03 mg/L이하	불검출
분원성대장균군/대장균	불검출/100mL	불검출	사염화탄소	0.002 mg/L이하	불검출
납	0.01 mg/L이하	불검출	유리잔류염소	4.0 mg/L이하	0.07
불소	1.5 mg/L이하	불검출	클로랄하이드레이트	0.03 mg/L이하	0.0017
비소	0.01 mg/L이하	불검출	디브로모아세토니트릴	0.1 mg/L이하	0.0018
셀레늄	0.01 mg/L이하	불검출	디클로로아세토니트릴	0.09 mg/L이하	불검출
수은	0.001 mg/L 이하	불검출	트리클로로아세토니트릴	0.004 mg/L이하	불검출
시안	0.01 mg/L이하	불검출	1,2-디브로모-3-클로로프로판	0.003 mg/L이하	불검출
크롬	0.05 mg/L이하	불검출	할로아세틱에시드	0.1 mg/L이하	0.002
암모니아성질소	0.5 mg/L이하	불검출	1,4-다이옥산	0.05 mg/L이하	불검출
질산성질소	10 mg/L이하	1.2	경도	300 mg/L이하	66
카드뮴	0.005 mg/L이하	불검출	과망간산칼륨소비량	10 mg/L이하	0.6
보론	1.0 mg/L이하	불검출	냄새	소독냄새 이외의 무취	없음
페놀	0.005 mg/L이하	불검출	맛	소독맛 이외의 무미	없음
다이아지논	0.02 mg/L이하	불검출	구리	1 mg/L 이하	불검출
파라티온	0.06 mg/L이하	불검출	색도	5 도이하	1
페니트로티온	0.04 mg/L이하	불검출	세제(음이온계면활성제)	0.5 mg/L이하	불검출
카바릴	0.07 mg/L이하	불검출	수소이온농도	5.8~8.5	7.1
총트리할로메탄	0.1 mg/L이하	0.041	아연	3 mg/L이하	불검출
클로로포름	0.08 mg/L이하	0.026	염소이온	250 mg/L이하	18.6
1,1,1-트리클로로에탄	0.1 mg/L이하	불검출	증발잔류물	500 mg/L이하	98
브로모디클로로메탄	0.03 mg/L이하	0.010	철	0.3 mg/L 이하	불검출
디브로모클로로메탄	0.1 mg/L이하	0.004	망간	0.05 mg/L이하	불검출
테트라클로로에틸렌	0.01 mg/L이하	불검출	탁도	0.5 NTU이하	0.18
트리클로로에틸렌	0.03 mg/L이하	불검출	황산이온	200 mg/L이하	12
디클로로메탄	0.02 mg/L이하	불검출	알루미늄	0.2 mg/L이하	0.03
벤젠	0.01 mg/L이하	불검출	포름알데히드	0.5 mg/L 이하	불검출
톨루엔	0.7 mg/L이하	불검출			
에틸벤젠	0.3 mg/L이하	불검출			

본 성적서는 시험의뢰 목적 이외의 광고, 상업적인 용도나 법적인 해결의 용도로 사용할수 없습니다.

워 터 스 생 활 환 경 연 구 소
국가공인 먹는물 수질검사기관 제38호

15850 경기도 군포시 고산로 148번길 17, A동 1204호 (당정동, 군포IT밸리) T.031)689-3231 F.031)689-3235

(END)

FINAL REPORT

SLC-D011:
A Maximum Tolerated Dose Study in Beagle Dogs following Escalated-Dose Oral Administration

Study Number: QT18142

Nonclinical Research Center
QuBEST BIO Co., Ltd.

www.QuBEST.co.kr TEL 031-706-2995 FAX 031-706-2996 E-mail info@qubest.co.kr
The BEST Partner for Your Successful Drug Development

STUDY DIRECTOR STATEMENT

STUDY TITLE: SLC-D011: A Maximum Tolerated Dose Study in Beagle Dogs following Escalated-Dose Oral Administration

STUDY NUMBER: QT18142

I, the undersigned, hereby declare that the work was performed under my supervision and that the report represents a true and accurate record of the results obtained.

This study was performed in accordance with the agreed protocol and with Standard Operating Procedures, unless otherwise stated, and the study objectives were achieved.
This study is not within the scope of regulations governing the conduct of nonclinical laboratory studies and is not intended to comply with such regulations.

TEST FACILITY 1304, U-TOWER, 120 Heungdeokjungang-ro, Giheung-gu, Yongin-si
Gyeonggi-do, 16950, Republic of Korea
TEL +82-31-706-2995, FAX +82-31-706-2996, Homepage: www.qubest.co.kr

Nonclinical Research Center, QuBEST BIO Co., Ltd.
#301, Daewoo Frontier Valley I, 16-25, Dongbaekjungang-ro 16beon-gil, Giheung-gu, Yongin-si, Gyeonggi-do, 17015, Republic of Korea

STUDY DIRECTOR SANGBUM WON, MS ________ NOV / 10 / 2018

MANAGEMENT SOOHYEON KIM, MS ________ NOV / 10 / 2018

TABLE OF CONTENTS

STUDY DIRECTOR STATEMENT 2

TABLE OF CONTENTS 3

1. SUMMARY 5

2. STUDY INTRODUCTION 6

2.1 STUDY TITLE 6

2.2 STUDY OBJECTIVE 6

2.3 REGULATORY GUIDELINES 6

2.4 SPONSOR REPRESENTATIVE 6

2.5 STUDY SCHEDULE 6

3. TEST ARTICLE AND VEHICLE 6

3.1 TEST ARTICLE 6

3.2 VEHICLE 7

4. ANIMALS AND HUSBANDRY 7

5. EXPERIMENTAL PROCEDURES 8

5.1 PREPARATION OF DOSE FORMULATIONS 8

5.2 DOSE FORMULATION ANALYSIS 8

5.3 JUSTIFICATION OF ADMINISTRATION ROUTE 8

5.4 GROUP ASSIGNMENT AND DOSE LEVEL 9

5.5 TREATMENT REGIMEN 9

5.6 ADMINISTRATION METHOD 9

6. OBSERVATIONS 9

6.1 MORTALITY AND CLINICAL OBSERVATIONS 9

6.2 BODY WEIGHTS 9

6.3 HEMATOLOGY EXAMINATION 9

6.4 SERUM CHEMISTRY EXAMINATION 10

6.5 TERMINAL PROCEDURES 10

7. RESULTS AND DISCUSSION 11
7.1 MORTALITY AND CLINICAL OBSERVATIONS 11
7.2 BODY WEIGHTS 11
7.3 HEMATOLOGY AND SERUM CHEMISTRY 11
8. CONCLUSION 11
9. APPENDICES 12
APPENDIX 1. MORTALITY AND CLINICAL OBSERVATIONS IN MALE DOGS (INDIVIDUAL) 13
APPENDIX 2. MORTALITY AND CLINICAL OBSERVATIONS IN FEMALE DOGS (INDIVIDUAL) 14
APPENDIX 3. BODY WEIGHTS IN MALE DOGS (INDIVIDUAL) 15
APPENDIX 4. BODY WEIGHTS IN FEMALE DOGS (INDIVIDUAL) 16
APPENDIX 5. HEMATOLOGICAL VALUES IN MALE DOGS (INDIVIDUAL) 17
APPENDIX 6. HEMATOLOGICAL VALUES IN FEMALE DOGS (INDIVIDUAL) 18
APPENDIX 7. WBC DIFFERENTIAL LEUKOCYTE COUNTS IN MALE DOGS (INDIVIDUAL) 19
APPENDIX 8. WBC DIFFERENTIAL LEUKOCYTE COUNTS IN FEMALE DOGS (INDIVIDUAL) 20
APPENDIX 9. SERUM CHEMISTRY VALUES IN MALE DOGS (INDIVIDUAL) 21
APPENDIX 11. SERUM CHEMISTRY VALUES IN FEMALE DOGS (INDIVIDUAL) 22
APPENDIX 10. CERTIFICATE OF ANALYSIS 23

1. SUMMARY

Objective

The objective of this study was to determine the maximum tolerated dose (MTD) of SLC-D011 following dose escalation by oral gavage to Beagle dogs.

Study Group

The study group was detailed in Text Table 1.

Text Table 1. Study Group

Group	Treatment	Dose Level (mg/kg)	Dose Conc. (mg/mL)	No. of Animals†	
				Males	Females
G1	SLC-D011	100	20	1	1
		400	80		

†: Animals were re-used for each dose with at least 4-day wash-out period.

Parameters Evaluated:

The following parameters were evaluated in this study: mortality, clinical signs, body weights and clinical pathology parameters (hematology and serum biochemistry).

Mortality and Clinical Observations

All animals survived until end of the study. Test article-related clinical observations were limited to oily soft stool in the 100 mg/kg/day group animals and vomitus (frothy) and soft stool in the 400 mg/kg/day group animals.

Body Weights

Test article-related lower body weights were temporarily noted in the 100 mg/kg/day group animals by Day 4.

Clinical Pathology Parameters (Hematology and Serum Chemistry)

There were no toxicologically significant changes in hematology and serum chemistry parameters in the 100 and 400 mg/kg group animals on approximately 24 hours post each dose, compared to pre dose values.

Based on the results of this study, the maximum tolerated dose (MTD) of SLC-D011 was considered to be > 400 mg/kg.

2. STUDY INTRODUCTION

2.1 STUDY TITLE

SLC-D011: A Maximum Tolerated Dose Study in Beagle Dogs following Escalated-Dose Oral Administration

2.2 STUDY OBJECTIVE

The objective of this study was to determine the maximum tolerated dose (MTD) of SLC-D011 following dose escalation by oral gavage to Beagle dogs.

2.3 REGULATORY GUIDELINES

No specific regulatory guidelines. This study was conducted according to study-specific protocol after consultation with the Sponsor.

2.4 SPONSOR REPRESENTATIVE

PRG S&T Co., Ltd. / Minju Kim, PhD / Phone: 051-510-7562 / E-mail: rlaals09@prgst.com / Room 306, Hyowon Industry-Cooperation Building, Pusan National University, 2, Busandaehak-ro 63beon-gil, Geumjeong-gu, Busan, 46241, Republic of Korea

2.5 STUDY SCHEDULE

Animal arrival:	Aug 31, 2018	
Administration:	Sep 6, 2018	(1st dosing)
	Sep 11, 2018	(2nd dosing)
Clinical observations:	Sep 6, 2018	~ Sep 10, 2018 (1st dosing)
	Sep 11, 2018	~ Sep 18, 2018 (2nd dosing)
Body weight measurement:	Sep 6 and 10 2018 (1st dosing)	
	Sep 11 and 18 2018 (2nd dosing)	
Blood collection for hematology	Sep 6, 2018	~ Sep 7, 2018 (1st dosing)
and chemistry:	Sep 11, 2018	~ Sep 12, 2018 (2nd dosing)
Final report:	Nov 10, 2018	

3. TEST ARTICLE AND VEHICLE

3.1 TEST ARTICLE

Identity:	SLC-D011
Batch/Lot Number:	A05064-013S2
Appearance:	White powder
Purity:	99.2% (by HPLC)

Storage conditions: Keep container tightly closed to avoid light in deep freezer (-70℃)
Handling precautions: Routine protection procedures with gloves, goggles etc. and avoid contact with skin, eye and inhalation of vapor/mist

The test article was provided by the Sponsor (Manufacturer: Pharmaron (Ningbo), Inc. under non-GLP/non-GMP condition). Additional information related to the test articles involved was provided by the Sponsor. The remaining test article was retained at test facility for future related studies.

3.2 VEHICLE

Identity: Oil based solution (Monoolein : Tricaprylin = 2 : 1)
Supplier: Nonclinical Research Center, QuBEST BIO Co., Ltd.
Appearance: Clear colorless liquid
Storage conditions: Room temperature

1) Vehicle Component 1

Identity: Monoolein
Batch/Lot number: RRNFJAT
Supplier: TCI (Tokyo Chemical Industry Co., LTD.)
Appearance: Clear colorless liquid
Storage conditions: Room temperature

2) Vehicle Component 2

Identity: Tricaprylin
Batch/Lot number: KEWXE-AL
Supplier: TCI (Tokyo Chemical Industry Co., LTD.)
Appearance: Clear colorless liquid
Storage conditions: Room temperature

4. ANIMALS AND HUSBANDRY

Non-naïve one male and one female Beagle dogs (approximately 16-24 months old, original supplier: ORIENTBIO Co., Ltd., Republic of Korea) were selected from the stock colony and assigned for the study.

The animals were individually housed in stainless steel cages [800W x 900L x 800H (mm)] equipped with a mesh floor and an automatic watering valve. All animals were housed throughout the acclimation period and during the study in an environmentally controlled room. The room temperature and humidity controls were set to maintain environmental conditions of 22±3℃ and 50±20%, respectively. Fluorescent lighting provided illumination for a 12-hour light (06:00 hours to 18:00

hours)/12-hour dark photoperiod. The 12-hour light/12-hour dark photoperiod was interrupted as necessary to allow the performance of protocol-specified activities. Air control units were set to provide a minimum of 10 fresh air changes per hour. During the study, animal room environment conditions were controlled within the target ranges.

A dog diet (Purina Canine Chow 38070, Cargill Inc., Republic of Korea, approximately 300 g once in the morning) and tap water via auto watering systems were provided *ad libitum*, except during designated procedures. Prior to each dosing, animals were fasted overnight and food was provided to animals approximately 4 hours post dose.

During the study, each animal was identified by cage label card displaying the study number, group, animal number and tattoo number.

5. EXPERIMENTAL PROCEDURES

5.1 PREPARATION OF DOSE FORMULATIONS

The dose formulations were freshly prepared in clean bench prior to dosing according to Sponsor's provided mixing procedure.

Monoolein based solution (Monoolein : Tricaprylin = 2 : 1) for PO

Pre-heated the monoolein based solution to a temperature above 80℃. Added pre-heating the monoolein based solution and performed sonication and vortexing until becomes a well suspension. And then warmed in hot water bath (80 - 100℃) until becomes a well suspension.

No purity correction was applied. During the dosing, formulations were handled at room temperature, the remaining was discarded.

5.2 DOSE FORMULATION ANALYSIS

Analysis of dose formulations such as stability, homogeneity and concentration verification was not performed in the test facility.

5.3 JUSTIFICATION OF ADMINISTRATION ROUTE

The route of administration was the oral (by gavage) route, which is the anticipated clinical route of exposure.

5.4 Group Assignment and Dose Level

Group	Treatment	Dose Level (mg/kg)	Dose Conc. (mg/mL)	No. of Animals†	
				Males	Females
G1	SLC-D011	100	20	1	1
		400	80		

†: Animals were re-used for each dose with at least 4-day wash-out period.

5.5 Treatment Regimen

Test article formulations were administered once via oral gavage. The day of 1st dosing was designated as Day 1.

5.6 Administration Method

Each dose was administered via a syringe attached with 12-french feeding tube. A dose volume of 5 mL/kg was used and individual dose volumes were based on the most recent body weight. Each dose was followed by a distilled water of 5 mL.

6. OBSERVATIONS

6.1 Mortality and Clinical Observations

Each animal was observed twice daily (a.m. and p.m.) for mortality and moribundity; findings were recorded as they were observed. Cage side observations were made for each animal once daily; abnormal findings were recorded. Detailed observations were made for each animal once prior to treatment; abnormal findings (ranked/graded, if appropriate) or an indication the animal appears normal was recorded.

6.2 Body Weights

Body weights were measured prior to each dosing.

6.3 Hematology Examination

Prior to each dosing and approximately 24 hours post each dose, blood samples for hematology examination were collected from the via cephalic vein into tubes with K_2EDTA anticoagulant and following parameters were examined (ADVIA®2120, Germany).

Parameters	Unit	Measurement Method
ⓐ RBC (Red blood cell count)	10^6/μL	Electric Impedance
ⓑ HCT (Hematocrit)	%	(RBC×MCV)÷10
ⓒ HGB (Hemoglobin conc.)	g/dL	Cyanmethemoglobin

ⓓ MCV (Mean corpuscular volume)	10^{-15} L	Histogram
ⓔ MCH (Mean corpuscular Hb)	10^{-12} g	10 x (Hb ÷ RBC)
ⓕ MCHC (Mean corpusc. Hb conc.)	g/dL	100 x (Hb ÷ Hct)
ⓖ PLT (Platelet)	10^3/μL	Electric Impedance
ⓗ Reti (Reticulocyte)	%	Flowcytometry, Isovolumetry
ⓘ WBC (White blood cell count)	10^3/μL	Electric Impedance
ⓙ NEU (Neutrophil)	%	Electric Impedance
ⓚ LYM (Lymphocyte)	%	Electric Impedance
ⓛ MONO (Monocyte)	%	Electric Impedance
ⓜ EOS (Eosinophil)	%	Electric Impedance
ⓝ BASO (Basophil)	%	Electric Impedance

6.4 Serum Chemistry Examination

Prior to dosing and approximately 24 hours post each dose, blood was collected as the same method and frequency with hematology analysis using no anticoagulant and then serum was separated by centrifugation and stored at freezer until analyze (AU 400, Olympus, Japan, RAPIDCHEM 744 Na^+/K^+/Cl^- Analyzer, SIEMENS, Germany).

Parameters	Unit	Measurement Method
ⓐ AST (Aspartate aminotransferase)	U/L	IFCC
ⓑ ALT (Alanine aminotransferase)	U/L	IFCC
ⓒ ALP (Alkaline phosphatase)	U/L	P-NPP
ⓓ BIL (Total bilirubin)	mg/dL	Evelyn-Malloy
ⓔ GLU (Glucose)	mg/dL	UV
ⓕ CHO (Total cholesterol)	mg/dL	Enzyme
ⓖ TG (Triglyceride)	mg/dL	Enzyme
ⓗ PRO (Total protein)	g/dL	Biuret
ⓘ ALB (Albumin)	g/dL	BCG
ⓙ A/G ratio (Albumin/Globulin ratio)	ratio	PRO/ALB
ⓚ BUN (Blood urea nitrogen)	mg/dL	Urease-UV
ⓛ CRE (Creatinine)	mg/dL	Jaffe
ⓜ Na^+ (Sodium ion)	mmol/L	Electrode method
ⓝ K^+ (Potassium ion)	mmol/L	Electrode method
ⓞ Cl^- (Chloride ion)	mmol/L	Electrode method

ⓐ-ⓛ: Measured by Blood chemistry analyzer (AU 400, Olympus, Japan)

ⓜ-ⓞ: Measured by blood electrolyte analyzer (RAPIDCHEM 744 Na^+/K^+/Cl^- Analyzer, SIEMENS, USA)

6.5 Terminal Procedures

All surviving animals were returned to the stock colony without necropsy.

7. RESULTS AND DISCUSSION

7.1 MORTALITY AND CLINICAL OBSERVATIONS

All animals survived until end of the study. Test article-related clinical observations of oily soft stool were noted in the 100 mg/kg/day group male and female, vomitus (frothy) and soft stool were noted in the 400 mg/kg/day group male and female.

7.2 BODY WEIGHTS

Test article-related lower body weights were noted in the 100 mg/kg/day group male and female by Day 4.

7.3 HEMATOLOGY AND SERUM CHEMISTRY

There were no toxicologically significant changes in hematology and serum chemistry parameters in the 100 and 400 mg/kg group male and female on approximately 24 hours post each dose, compared to pre dose values.

8. CONCLUSION

Based on the results of this study, single oral administration of SLC-D011 to Beagle dogs at dose levels of 100 and 400 mg/kg was well tolerated.

In conclusion, the maximum tolerated dose (MTD) of SLC-D011 was considered to be > 400 mg/kg.

9. APPENDICES

(Individual)

APPENDIX 1. MORTALITY AND CLINICAL OBSERVATIONS IN MALE DOGS (INDIVIDUAL)

Group/ Treatment	Dose Level (mg/kg)	No. of Animals	Clinical Observations				Mortality (%)
			Day 1	Day 2	Day 3	Day 4	
G1 SLC-D011	100	1	Brown soft stool (Oily) (about 7 hours post dose)	Appears normal	Appears normal	Appears normal	0/1 (0)
	400	1	Vomitius (Frothy) (about 1 hours post dose)	Appears normal	Appears normal	Appears normal	0/1 (0)

(END)

APPENDIX 2. MORTALITY AND CLINICAL OBSERVATIONS IN FEMALE DOGS (INDIVIDUAL)

Group/ Treatment	Dose Level (mg/kg)	No. of Animals	Clinical Observations				Mortality (%)
			Day 1	Day 2	Day 3	Day 4	
G1 SLC-D011	100	1	Soft stool (Oily) (about 7 hours post dose)	Appears normal	Appears normal	Appears normal	0/1 (0)
	400	1	Black soft stool (about 6 hours post dose)	Appears normal	Appears normal	Appears normal	0/1 (0)

(END)

APPENDIX 3. BODY WEIGHTS IN MALE DOGS (INDIVIDUAL)

Group/ Treatment	Dose Level (mg/kg)	No. of Animals	Body Weights (kg)	
			Day 1	Day 4
G1 SLC-D011	100	1	8.4	8.2
	400	1	8.2	-

-: Not applicable

(END)

APPENDIX 4. BODY WEIGHTS IN FEMALE DOGS (INDIVIDUAL)

Group/ Treatment	Dose Level (mg/kg)	No. of Animals	Body Weights (kg)	
			Day 1	Day 4
G1 SLC-D011	100	1	8.5	8.3
	400	1	8.2	-

-: Not applicable

(END)

APPENDIX 5. HEMATOLOGICAL VALUES IN MALE DOGS (INDIVIDUAL)

Group/ Treatment	Dose Level (mg/kg)	Animal No.	Hematological Values								
			WBC K/uL	RBC M/uL	HGB g/uL	HCT %	MCV fL	MCH pg	MCHC g/dL	PLT K/uL	Reti %
G1 SLC-D011	Prior to Dosing	M1	8.46	6.77	15.1	46.9	69.3	22.3	32.2	361	0.22
	100	M1	8.83	6.69	15.4	46.8	69.9	23.0	33.0	354	0.15
	Prior to Dosing	M1	8.72	7.44	17.1	52.9	71.1	22.9	32.3	354	0.60
	400	M1	9.43	6.90	15.2	50.5	73.2	22.1	30.1	347	0.34

(END)

APPENDIX 6. HEMATOLOGICAL VALUES IN FEMALE DOGS (INDIVIDUAL)

Group/ Treatment	Dose Level (mg/kg)	Animal No.	Hematological Values								
			WBC K/uL	RBC M/uL	HGB g/uL	HCT %	MCV fL	MCH pg	MCHC g/dL	PLT K/uL	Reti %
G1 SLC-D011	Prior to Dosing	F1	7.85	6.76	15.4	46.4	68.7	22.8	33.2	386	0.54
	100	F1	7.80	6.93	15.6	47.8	68.9	22.5	32.6	357	0.61
	Prior to Dosing	F1	10.02	6.85	15.7	47.6	69.4	22.9	33.0	389	0.62
	400	F1	10.99	7.08	15.7	50.8	71.7	22.2	30.9	378	0.95

(END)

APPENDIX 7. WBC DIFFERENTIAL LEUKOCYTE COUNTS IN MALE DOGS (INDIVIDUAL)

Group/ Treatment	Dose Level (mg/kg)	Animal No.	WBC Differential Leukocyte Counts				
			NEU	LYM	MONO	EOS	BASO
			%	%	%	%	%
G1 SLC-D011	Prior to Dosing	M1	57.2	28.9	6.3	7.0	0.4
	100	M1	55.7	32.4	7.1	4.3	0.2
	Prior to Dosing	M1	60.4	27.7	6.9	4.5	0.4
	400	M1	61.7	25.1	7.9	4.4	0.6

(END)

APPENDIX 8. WBC DIFFERENTIAL LEUKOCYTE COUNTS IN FEMALE DOGS (INDIVIDUAL)

Group/ Treatment	Dose Level (mg/kg)	Animal No.	WBC Differential Leukocyte Counts				
			NEU %	LYM %	MONO %	EOS %	BASO %
G1 SLC-D011	Prior to Dosing	F1	61.3	31.6	4.6	1.4	0.7
	100	F1	52.3	39.9	5.6	1.2	0.6
	Prior to Dosing	F1	61.4	30.0	6.0	1.8	0.5
	400	F1	59.6	31.2	6.5	1.4	0.8

(END)

APPENDIX 9. SERUM CHEMISTRY VALUES IN MALE DOGS (INDIVIDUAL)

Group/ Treatment	Dose Level (mg/kg)	Animal No.	Serum Biochemistry Values														
			AST U/L	ALT U/L	ALP U/L	T-BIL mg/dL	GLU mg/dL	CHO mg/dL	TG mg/dL	PRO g/dL	ALB g/dL	A/G	BUN mg/dL	CRE mg/dL	Na^+ mmol/L	K^+ mmol/L	Cl^- mmol/L
G1 SLC-D011	Prior to Dosing	M1	26.8	46.3	65.2	0.13	103.31	151.8	27.7	103.31	2.45	0.60	14.2	0.77	144	5	114
	100	M1	28	43.1	83.4	0.1	95.73	161	28.9	95.73	2.41	0.58	16.83	0.7	144	4.8	117
	Prior to Dosing	M1	51.3	90.4	85.3	0.15	99.22	139.1	21.9	99.22	2.47	0.62	13.16	0.75	146	5.2	117
	400	M1	27.2	74.9	94.3	0.12	98.14	142	34.4	98.14	2.37	0.59	16.17	0.79	146	5.1	116

(END)

APPENDIX 11. SERUM CHEMISTRY VALUES IN FEMALE DOGS (INDIVIDUAL)

Group/ Treatment	Dose Level (mg/kg)	Animal No.	Serum Biochemistry Values														
			AST U/L	ALT U/L	ALP U/L	T-BIL mg/dL	GLU mg/dL	CHO mg/dL	TG mg/dL	PRO g/dL	ALB g/dL	A/G	BUN mg/dL	CRE mg/dL	Na^+ mmol/L	K^+ mmol/L	Cl^- mmol/L
G1 SLC-D011	Prior to Dosing	F1	34.3	33.7	103.3	0.12	107.46	187.7	21.9	6.22	2.66	0.75	14.14	0.77	144	4.7	114
	100	F1	41.7	34.3	106.7	0.18	96.4	189.4	21.3	6.27	2.62	0.72	17.7	0.82	141	4.5	115
	Prior to Dosing	F1	45.2	38.6	174.4	0.13	93.11	172.5	23.1	5.96	2.62	0.78	12.41	0.84	142	4.7	113
	400	F1	34.5	39.1	174.7	0.15	104.39	175.1	29.3	5.76	2.53	0.78	12.08	0.85	143	4.9	117

(END)

APPENDIX 10. CERTIFICATE OF ANALYSIS

康龙化成 PHARMARON

Report of Analysis

Project ID	PH-QBC-SLC-D011	Version	V01
Batch Number	A05064-013S2	Client ID	SLC-D011
Manufacture Date	8Aug2018	Batch Size	23.5 g
Test Date	13Aug2018	Retest Date	12Aug2019
Storage Condition	Stored in well closed container at ambient temperature		
Note	All tests were performed in non-GMP analytical laboratories.		

Tested Items	Method	Specification	Result
Appearance	Visual	Report	White powder
^{1}H-NMR	NMR	Consistent with structure	Consistent with structure
LCMS	LCMS	Consistent with exact mass	Consistent with exact mass
Purity (area%)	HPLC	≥ 97.5%	99.2%
Chiral Purity (area%)	Chiral HPLC	≥99%	100%

Conclusion: All results meet the acceptance criteria in the specification

Revision Log:

Author	Description	Effective Date
Quanyin Guan	Original Issue	17Aug2018

Prepared by/Date : Quanyin Guan 17Aug2018

Reviewed by/Date : Xiaotong [illegible] 17Aug2018

Address: Pharmaron (Ningbo), Inc. No.800 Bin-Hai 4th Road, Hangzhou Bay New Zone, Ningbo, China315336. Tel: 0574-23450698

(END)
