# Supplementary material for: Progerinin, an optimized progerin-lamin A binding inhibitor, ameliorates premature senescence phenotypes of Hutchinson-Gilford progeria syndrome
Source: Commun Biol. 2021 Jan 4;4:5. doi: 10.1038/s42003-020-01540-w (PMC7782499; doi:10.1038/s42003-020-01540-w)

Chemical synthesis

Figure 1. Synthetic Scheme for the Manufacturing Process of SLC-D011

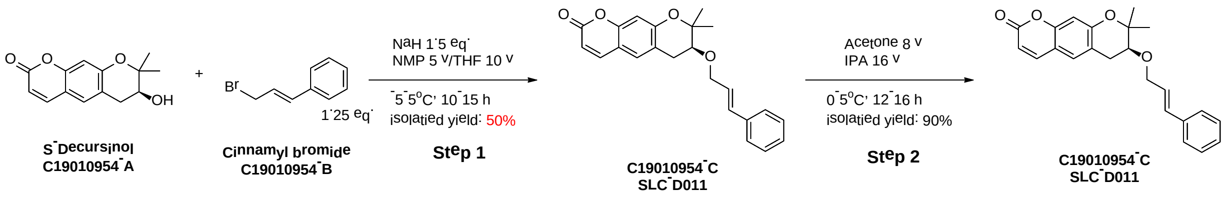

^{1}H-NMR Spectroscopy

The $_1$H-NMR spectrum of SLC-D011 was analyzed on a JNM-AL 400 spectrometer (400MHz, JEOL, Japan).

Table 1. ^{1}H-NMR Chemical Shift and Assignment of SLC-D011 (400 MHz, $CDCl_3$)

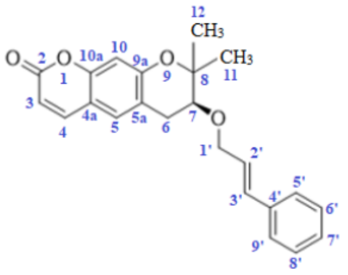

Chemical Shift (ppm)	Number of Protons	Multiplicity	Assignment
1.46	3	s	CH_3 (11)
1.52	3	s	CH_3 (12)
2.96	1	dd, J = 7.26, 16.5Hz	-CH_2-, (6)
3.18	1	d, J = 4.91, 16.48Hz	-CH_2-, (6)
3.69	1	dd, J = 5.04, 7.16Hz	-CH, (7)
4.32	1	dd, J = 6.33, 12.90Hz	Vinyl-CH_2-, (1’)
4.44	1	dd, J = 5.83, 12.87Hz	Vinyl-CH_2-, (1’)
6.31	1	d, J = 9.48Hz	=CH-CO- (3)
6.37	1	m	C=CH-R (2’)
6.69	1	d, J = 15.98Hz	C=CH-Ar (3’)
6.87	1	s	Ar-H (10)
7.26	1	s	Ar-H (5)
7.36	1	dd, J=3.02, 3.91Hz	Ar-H (7’)
7.41	2	d, J=7.69Hz	Ar-H (5’, 9’)
7.47	2	t, J=6.46Hz	Ar-H (6’, 8’)
7.67	1	d, J = 9.49Hz	C=CH-Ar (4)

s: singlet, d: doublet, dd : doublet of doublet, t : triplet, m : multiplet

Figure 2. ^{1}H-NMR Spectrum of SLC-D011

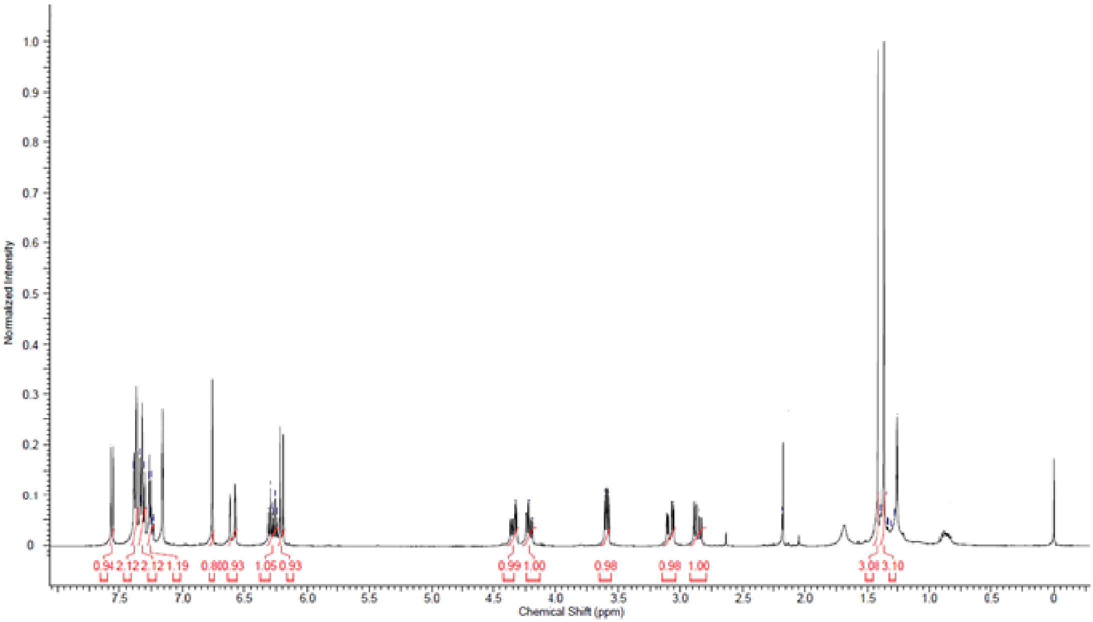

^{1}H NMR(400MHz, $CDCl_3$): δ_H 7.56(1H, d, *J*= 9.6Hz, H-4), 7.38-7.23(5H, m, H-5’, H-6’, H-7’, H-8’, H-9’), 7.15(1H, s, H-5), 6.76(1H, s, H-10), 6.59(1H, d, *J*= 16.0Hz, H-3’), 6.30-6.23(1H, m, H-2’), 6.20(1H, d, *J*= 9.6Hz, H-3), 4.34(1H, dd, *J*= 6.0, 12.8Hz, H-1a’), 4.21(1H, dd, *J*= 6.0, 12.4Hz, H-1b’), 3.59(1H, dd, *J*= 5.2, 7.6Hz, H-7), 3.07(1H, dd, *J*= 4.8, 16.0Hz, H-6a), 2.85(1H, dd, *J*= 7.2, 16.4Hz, H-6b), 1.41(3H, s CH_3-8), 1.36(3H, s, CH_3-8)

^{13}C-NMR Spectroscopy

Table 2. ^{13}C-NMR Chemical Shift and Assignment of SLC-D011 (100 MHz, $CDCl_3$)

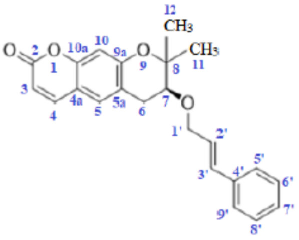

Chemical Shift (ppm)	Assignment
161.5	C-2
156.8	C-9a
154.3	C-10a
143.3	C-4
136.5	C-4’
132.8	C-3’
128.9	C-5

128.7	C-6’, C-8’
128.0	C-7’
126.6	C-5’, C-9’
126.0	C-2’
117.2	C-5a
112.8	C-4a
113.2	C-3
104.8	C-10
78.1	C-8
75.7	C-7
70.7	C-1’
27.7	C-6
26.0	C-12
21.7	C-11

Figure 3. ^{13}C-NMR Spectrum of SLC-D011

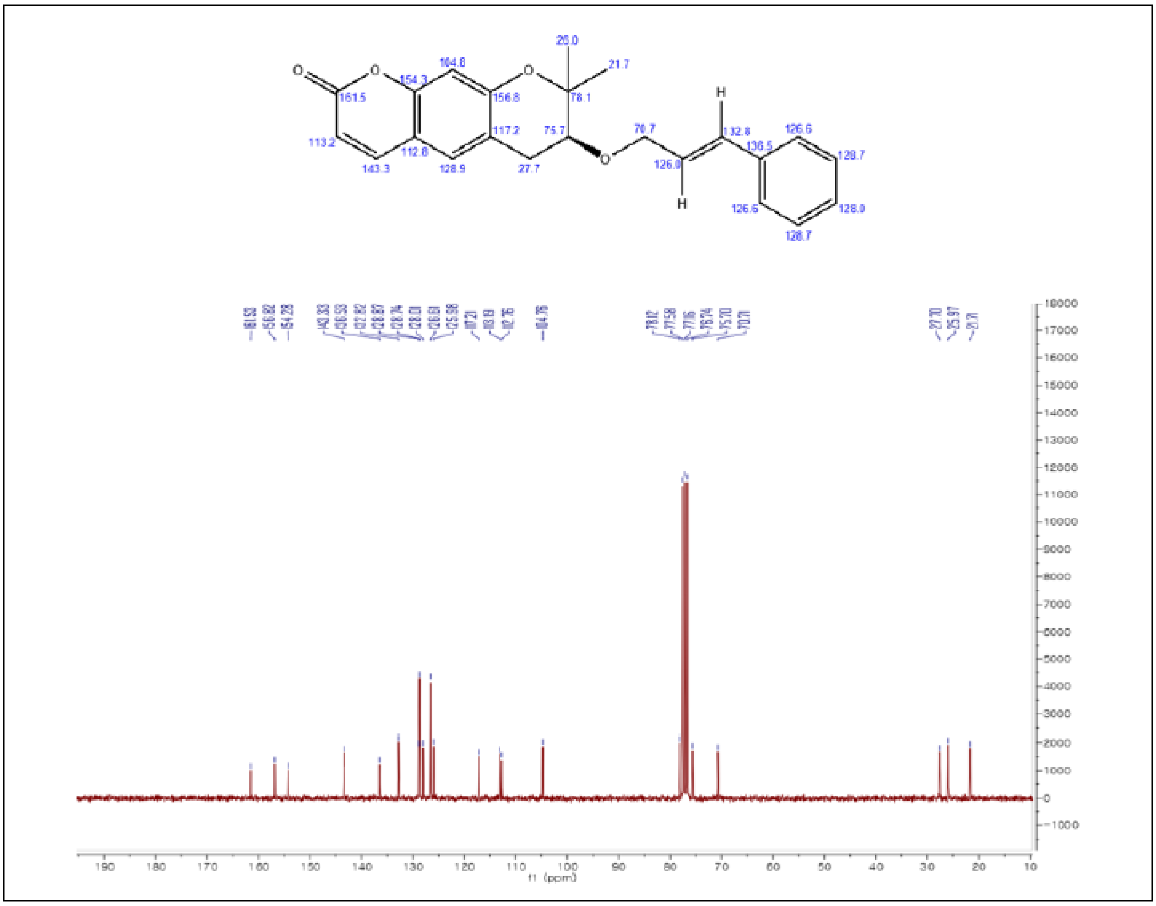

Mass Spectrometry

A mass spectrum analysis was performed on an API 2000 LC/MS/MS spectrometer (PE Sciex, Canada). In the mass spectrum, the molecular ion peak was observed at m/z=363 $[M+H]^+$ (exact mass: 362.43), consistent with the proposed chemical structure of SLC-D011.

Figure 4. Mass Spectrum of SLC-D011

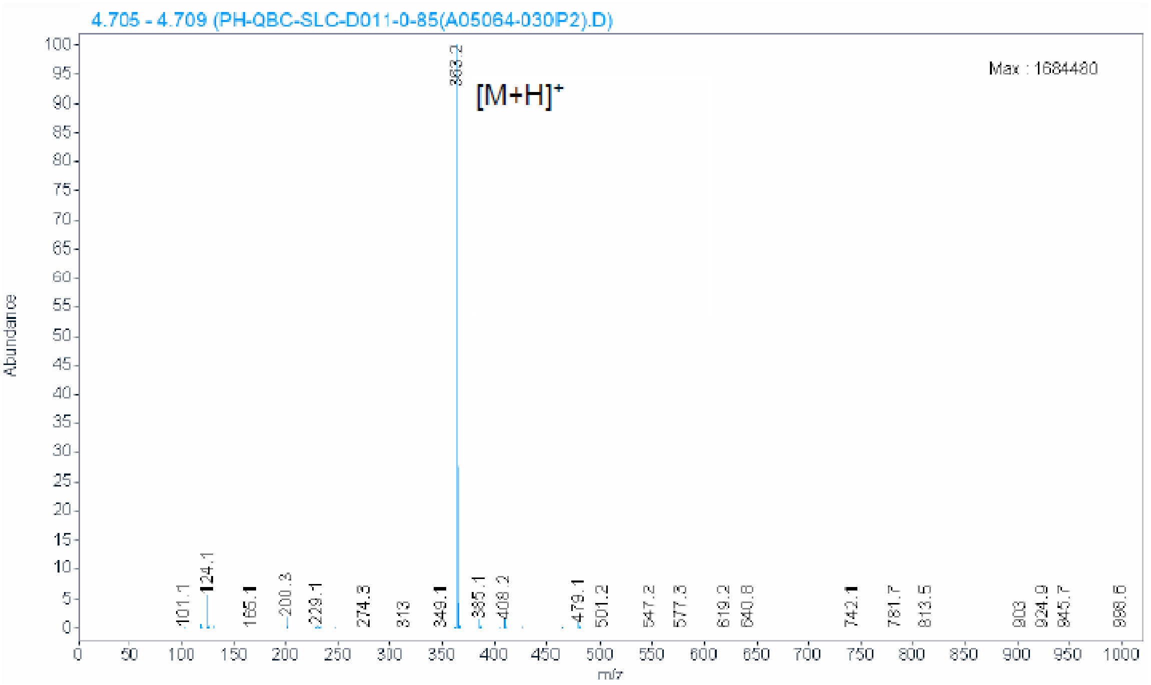

X-ray Diffractometry

Polymorph screening was performed on SLC-D011 using Bruker D8 ADVANCE X-ray Powder Diffractometer. The diffractometer was equipped with LynxEye (ID mode) detector. In XRPD analysis, the samples were scanned from 4 to 40o at a step size of 0.02o. The tube voltage and current were 40 KV and 40 mA, respectively.

Figure 5. X-Ray Diffractogram of SLC-D011

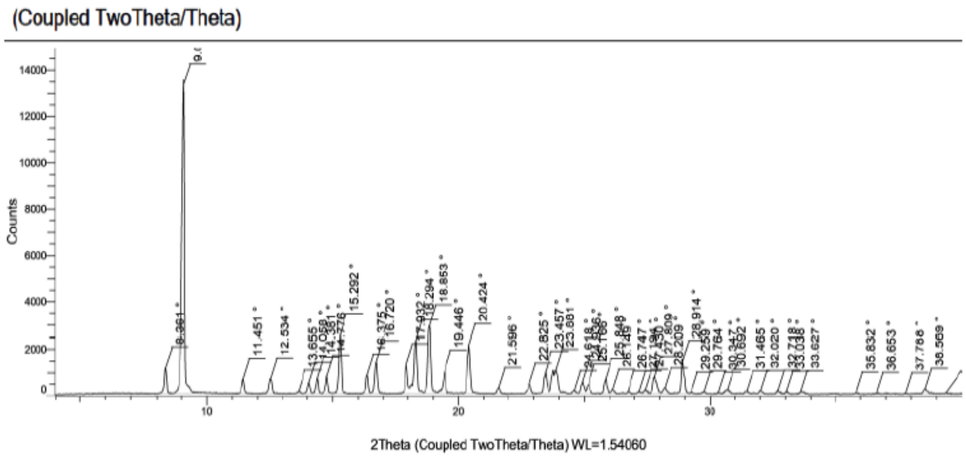

Supplement: Supplementary file 5 — Supplementary Data 2 [file 42003_2020_1540_MOESM5_ESM.pdf]
