# Supplementary material for: Progerinin, an optimized progerin-lamin A binding inhibitor, ameliorates premature senescence phenotypes of Hutchinson-Gilford progeria syndrome
Source: Commun Biol. 2021 Jan 4;4:5. doi: 10.1038/s42003-020-01540-w (PMC7782499; doi:10.1038/s42003-020-01540-w)
Supplement: Supplementary file 7 — Reporting Summary [file 42003_2020_1540_MOESM7_ESM.pdf]

nature research

Corresponding author(s): Bum-Joon Park

Last updated by author(s): Oct 31, 2020

Reporting Summary

Nature Research wishes to improve the reproducibility of the work that we publish. This form provides structure for consistency and transparency in reporting. For further information on Nature Research policies, see our Editorial Policies and the Editorial Policy Checklist.

Statistics

For all statistical analyses, confirm that the following items are present in the figure legend, table legend, main text, or Methods section.

n/a	Confirmed	
☐	☒	The exact sample size (n) for each experimental group/condition, given as a discrete number and unit of measurement
☐	☒	A statement on whether measurements were taken from distinct samples or whether the same sample was measured repeatedly
☐	☒	The statistical test(s) used AND whether they are one- or two-sided<br>*Only common tests should be described solely by name; describe more complex techniques in the Methods section.*
☐	☒	A description of all covariates tested
☐	☒	A description of any assumptions or corrections, such as tests of normality and adjustment for multiple comparisons
☐	☒	A full description of the statistical parameters including central tendency (e.g. means) or other basic estimates (e.g. regression coefficient) AND variation (e.g. standard deviation) or associated estimates of uncertainty (e.g. confidence intervals)
☐	☒	For null hypothesis testing, the test statistic (e.g. F, t, r) with confidence intervals, effect sizes, degrees of freedom and P value noted<br>*Give P values as exact values whenever suitable.*
☒	☐	For Bayesian analysis, information on the choice of priors and Markov chain Monte Carlo settings
☒	☐	For hierarchical and complex designs, identification of the appropriate level for tests and full reporting of outcomes
☒	☐	Estimates of effect sizes (e.g. Cohen's d, Pearson's r), indicating how they were calculated

Our web collection on statistics for biologists contains articles on many of the points above.

Software and code

Policy information about availability of computer code

Data collection	No software was used.
Data analysis	Image J was used for quantification of WB and IF images.<br>TOMTEC system was used for analyzing heart beat of mice.<br>RadiAnt DICOM system was used for analyzing X-ray data and kyposis index of mice.

For manuscripts utilizing custom algorithms or software that are central to the research but not yet described in published literature, software must be made available to editors and reviewers. We strongly encourage code deposition in a community repository (e.g. GitHub). See the Nature Research guidelines for submitting code & software for further information.

Data

Policy information about availability of data

All manuscripts must include a data availability statement. This statement should provide the following information, where applicable:

- Accession codes, unique identifiers, or web links for publicly available datasets
- A list of figures that have associated raw data
- A description of any restrictions on data availability

The datasets generated during and/or analysed during the current study are available from the corresponding author on reasonable request.

Field-specific reporting

Please select the one below that is the best fit for your research. If you are not sure, read the appropriate sections before making your selection.

- [x] Life sciences
- [ ] Behavioural & social sciences
- [ ] Ecological, evolutionary & environmental sciences

For a reference copy of the document with all sections, see nature.com/documents/nr-reporting-summary-flat.pdf

Life sciences study design

All studies must disclose on these points even when the disclosure is negative.

Sample size	Sample size was determined based on our previous reports or similar studies in this field.
Data exclusions	No data was excluded.
Replication	All replication attempt were successful in western blot, RT-PCR, IF, and in vivo experiments. For final quantification, all samples were quantified for a minimum of three samples.
Randomization	Cell counting images were selected randomly. However western blot analysis was performed 3 times individually and quantified, there was no requirement for randomization.
Blinding	The investigators were not blinded during data collection, because experimental conditions should be equal in every experiment.

Reporting for specific materials, systems and methods

We require information from authors about some types of materials, experimental systems and methods used in many studies. Here, indicate whether each material, system or method listed is relevant to your study. If you are not sure if a list item applies to your research, read the appropriate section before selecting a response.

Materials & experimental systems

n/a	Involved in the study	
[ ]	[x]	Antibodies
[ ]	[x]	Eukaryotic cell lines
[x]	[ ]	Palaeontology and archaeology
[ ]	[x]	Animals and other organisms
[x]	[ ]	Human research participants
[x]	[ ]	Clinical data
[x]	[ ]	Dual use research of concern

Methods

n/a	Involved in the study	
[x]	[ ]	ChIP-seq
[x]	[ ]	Flow cytometry
[x]	[ ]	MRI-based neuroimaging

Antibodies

Antibodies used	Antibodies used for experiments included anti-GFP (1:1000; sc-9996; Santa Cruz Biotechnology, Dallas, TX, USA); anti-GST (1:5000; sc-138; Santa Cruz Biotechnology), anti-His (1:1000; 66005-1-lg; Proteintech, Rosemont, IL, USA), anti-Actin (1:10000; sc-47778; Santa Cruz Biotechnology), anti-Lamin A/C (1:10000; sc-376248; Santa Cruz Biotechnology), anti-lamin A/C (1:500; MANLAC1; Developmental Studies Hybridoma Bank), anti-Progerin (1:100; sc-81611; Santa Cruz Biotechnology), anti-Progerin (1:300; ab66587; Abcam, Cambridge, UK), anti-Ki67 (1:200; Ab15580; Abcam), anti-cyclin B1 (1:100; sc-594; Santa Cruz Biotechnology), and anti-p16-INK4A (1:500; 10883-1-AP; Proteintech).
Validation	Anti-lamin A/C (MANLAC1; Developmental Studies Hybridoma Bank) antibody has been validated by DSHB by demonstrating immunoblotthing on mouse tissue extract. This antibody was also validated in a previous report (O Santiago-Fernández et al. 2019).

Eukaryotic cell lines

Policy information about cell lines

Cell line source(s)	HEK293 cells (ATCC)<br>AG03198, AG03199, AG11513, AG11498 (Coriell Cell Repositories)<br>N9 (GM00038, Coriell Cell Repositories)
Authentication	None of the cell lines have been authenticated.
Mycoplasma contamination	Cell lines were not tested for mycoplasma contamination but no indication of contamination was observed.

Commonly misidentified lines (See ICLAC register)	No commonly misidentified cell lines were used.

Animals and other organisms

Policy information about studies involving animals; ARRIVE guidelines recommended for reporting animal research

Laboratory animals	Wild-type, LmnaG609G progeroid mice (male and female) were used for in vivo experiments.
Wild animals	No wild animals were used in this study.
Field-collected samples	No field-collected samples were used in this study.
Ethics oversight	Animal experiments were performed in a facility certified by the Association for Assessment and Accreditation of Laboratory Animal Care in compliance with animal policies approved by Pusan National University. The mouse work was performed under the study protocol PNU-2019-2181, as approved by the Institutional Animal Care and Use Committee.

Note that full information on the approval of the study protocol must also be provided in the manuscript.
